# Supplementary material for: Genome-wide spatial expression profiling in formalin-fixed tissues
Source: Cell Genom. 2021 Dec 8;1(3):100065. doi: 10.1016/j.xgen.2021.100065 (PMC9903805; doi:10.1016/j.xgen.2021.100065)
Supplement: Document S1. Figures S1–S20 [file mmc1.pdf]

Cell Genomics, Volume *1*

Supplemental information

Genome-wide spatial expression profiling in formalin-fixed tissues

Eva Gracia Villacampa, Ludvig Larsson, Reza Mirzazadeh, Linda Kvastad, Alma Andersson, Annelie Mollbrink, Georgia Kokaraki, Vanessa Monteil, Niklas Schultz, Karin Sofia Appelberg, Nuria Montserrat, Haibo Zhang, Josef M. Penninger, Wolfgang Miesbach, Ali Mirazimi, Joseph Carlson, and Joakim Lundeberg

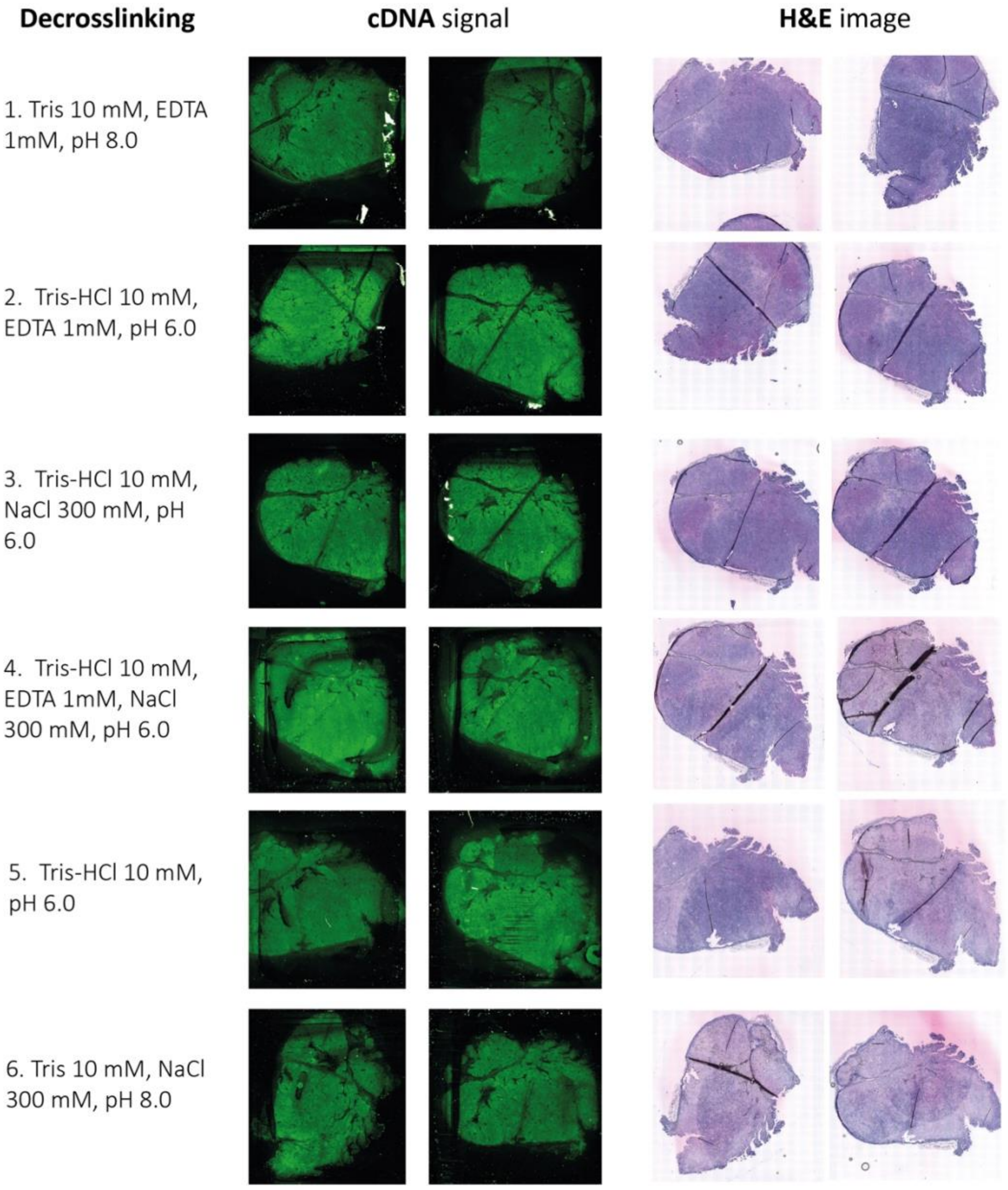

Figure S1. Evaluation of decrosslinking buffer conditions, related to STAR Methods.

Six different buffers with variable pH, EDTA and NaCl concentrations were evaluated using a tissue optimization (TO) assay on FFPE tissue sections. In summary, cDNA footprints are generated through incorporation of Cy3 fluorescently labelled nucleotides by second strand synthesis, where signal intensity is approximately proportional to cDNA concentration on the surface (middle column). No apparent difference was observed in the cDNA signal generated using the six different buffers. H&E images of the tissue sections are shown in the right column.

Occasional variation in the Cy3 signal, for example the darker bands visible in condition 4, are likely produced in the array printing process of the TO slides.

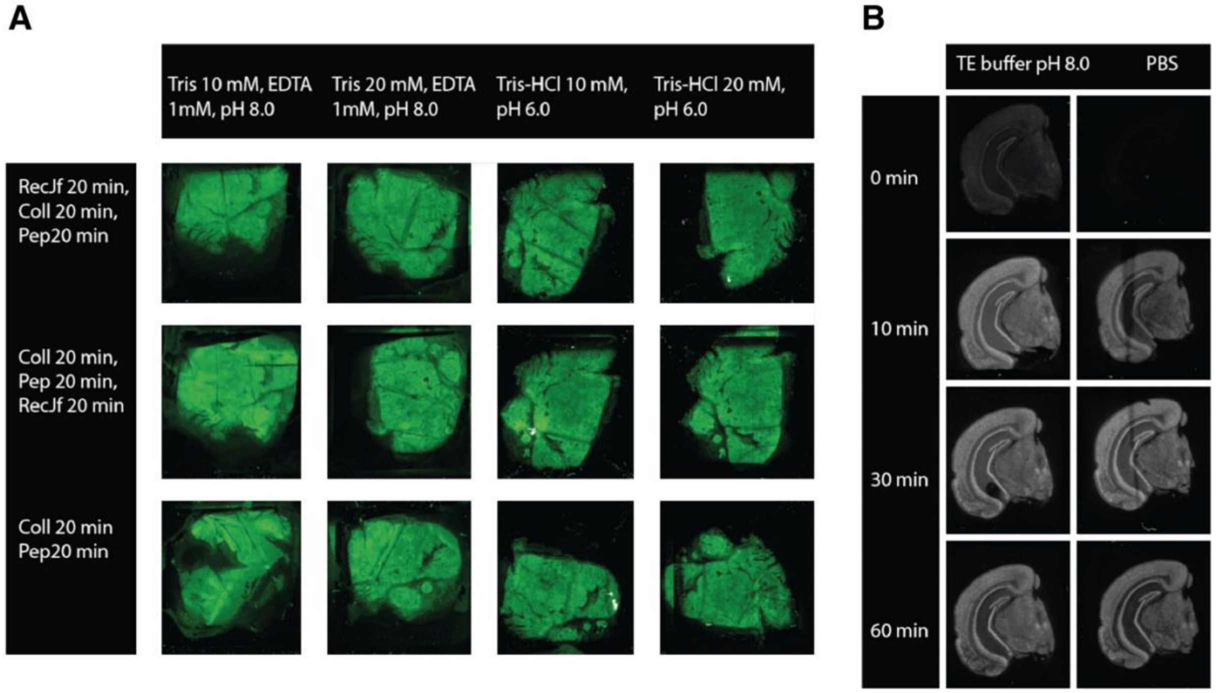

Figure S2. Evaluation of decrosslinking buffers and permeabilization conditions, related to STAR Methods.

cDNA footprints are generated through incorporation of Cy3 fluorescently labelled nucleotides by second strand synthesis. Signal intensity is approximately proportional to cDNA concentration on the surface.

(A) Evaluation of crosslink buffers for cDNA synthesis on FFPE tissue sections. Four crosslink buffers were tested (columns) in combination with three permeabilization conditions (rows). No apparent difference in cDNA signal was observed between the conditions.

(B) Pepsin treatment optimization on Mouse Brain tissue sections. TE buffer pH 8.0 (left column) was compared with PBS buffer (right column) at four different permeabilization times. Variation in the Cy3 signal observed in PBS 10 min and PBS 30 min (dark band), is likely an artefact produced in the array printing process of the TO slides.

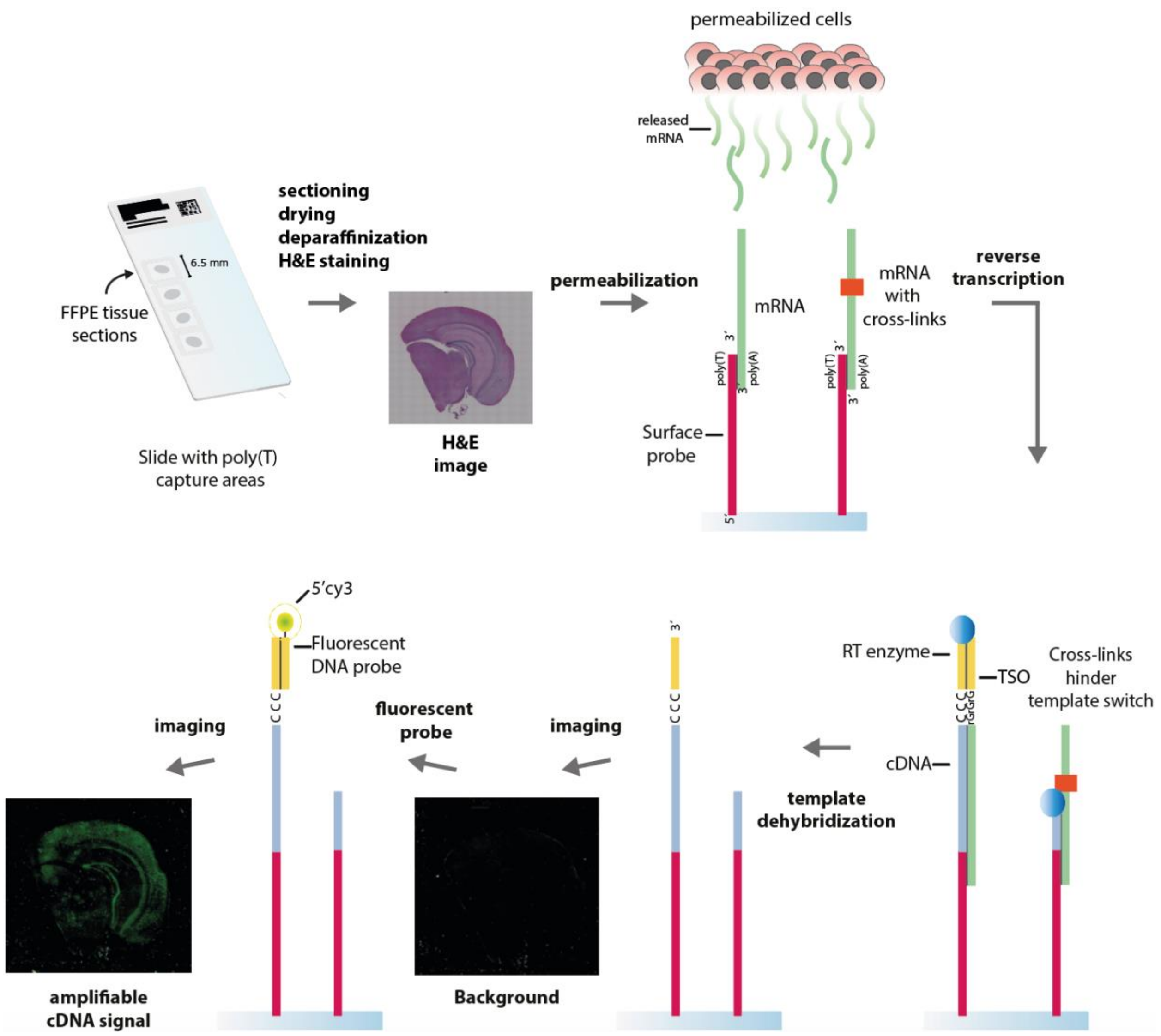

Figure S3. TSO-based QC assay for FFPE samples, related to STAR Methods.

A slide with polyT probes is used, sections of tissue are placed on the capture areas. The FFPE sections are dried, deparaffinized, stained with H&E and imaged under a high-resolution microscope. During permeabilization, the mRNA molecules diffuse outside the cell and their poly(A) tails hybridize the poly(T) of the surface probes. During reverse transcription a cDNA strand is synthesized and a complementary to TSO sequence added in the 3´end. Then the tissue is removed and the mRNA templates are de-hybridized. Slides are imaged to ensure there is no

tissue left over the slide. Finally, a fluorescent cy3-TSOprobe is hybridized and the cDNA signal image is taken.

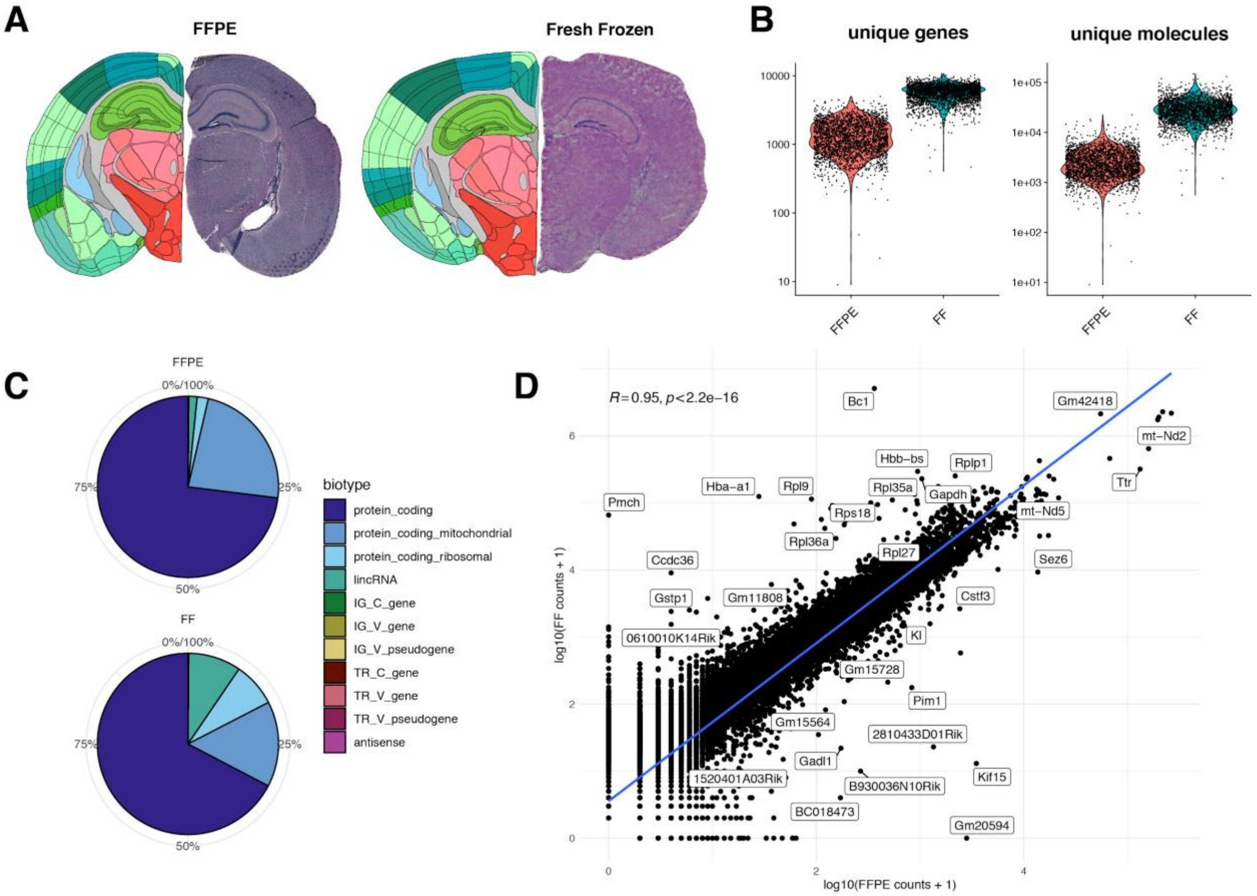

Figure S4. Image registration, quality control and correlation of FFPE and FF tissue sections, related to Figure 2 and STAR Methods.

(A) H&E images of the FFPE and Fresh Frozen (FF) tissue sections (right hemisphere) next to the registered anatomical reference from the Allen Mouse Brain Atlas (left hemisphere). The registration was done using the *wholebrain* framework with the stereotactic coordinate set to -2.2 from bregma along the anterior-posterior axis for both tissue sections.

(B) Quality metrics shown as violin plots for the FFPE and FF sections (y-axis in log-scale). The median number of unique genes per spot was ~1200 and ~6000 respectively. The median number of unique molecules (UMIs) per spot was ~2200 for the FFPE section and ~27200 for the FF section.

(C) Composition of RNA biotypes in the two tissue sections. Notably, the FFPE section contained a higher fraction of mitochondrial protein coding genes and a lower fraction of both ribosomal protein coding genes as well as lncRNA.

(D) Scatter plot showing the correlation between log10-transformed UMI counts per gene for two tissue sections with a Pearson correlation coefficient of 0.95. Highlighted outlier genes indicate that hemoglobin genes *Hba-a1* and *Hbb-bs* and certain ribosomal protein genes are more highly expressed in the fresh frozen tissue section.

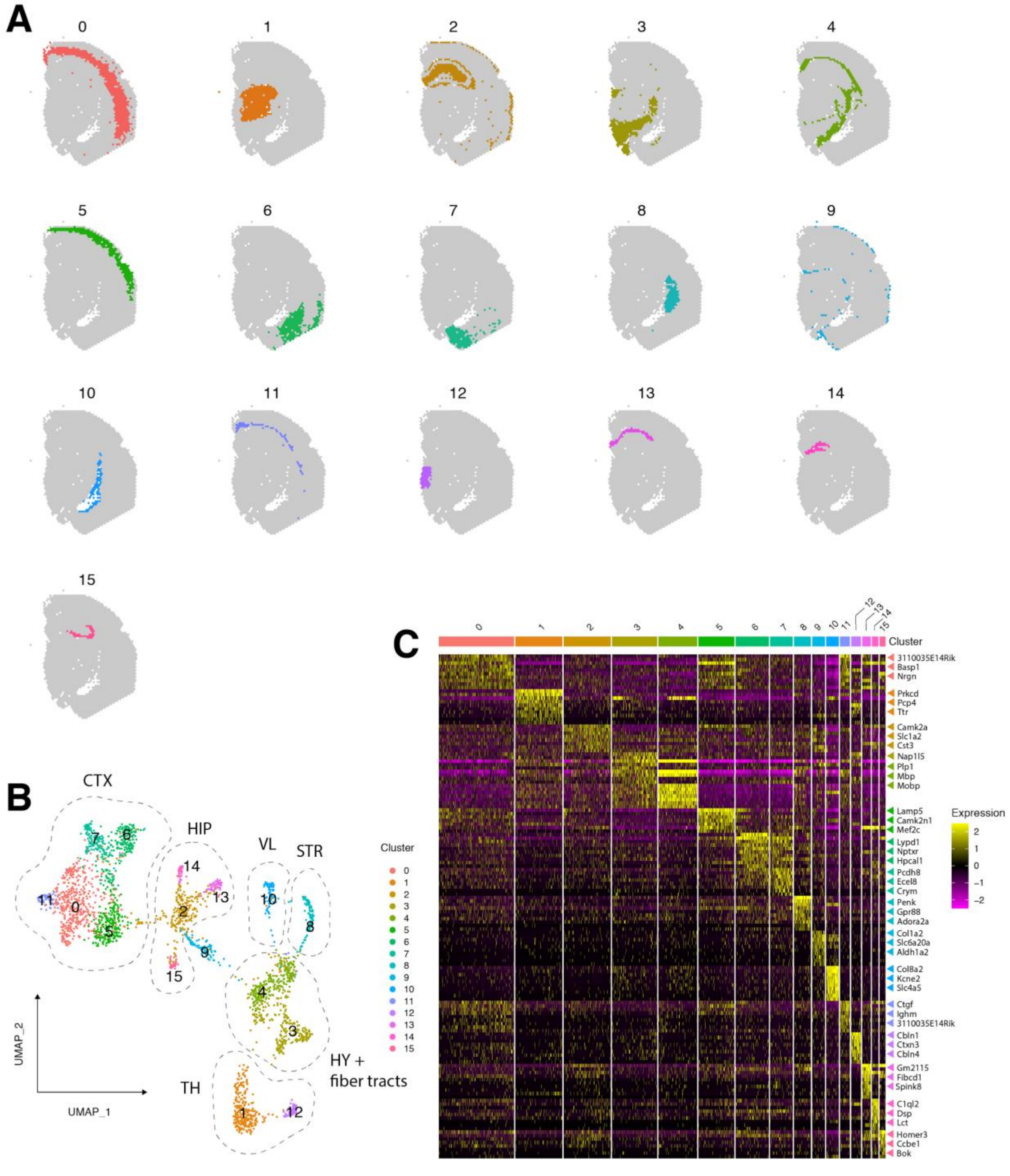

Figure S5. Clustering and marker detection in a coronal section from FFPE mouse brain tissue, related to Figure 2 and STAR Methods.

(A) Spatial mapping of 16 clusters on tissue section spot coordinates.

(B) 2D UMAP embedding of spatial gene expression data colored by cluster. Clusters 2, 13, 14 and 15 form the hippocampal region (HIP) where cluster 13 maps to field 1 of the pyramidal layer (CA1sp), cluster 14 maps to the dentate gyrus (DG-sg) region and cluster 15 maps to field 3 of

the pyramidal layer (CA3sp) region. Cluster 2 shows a less specific pattern in **A** where it makes up the majority of the HIP and also part of the outermost layer of the cerebral cortex (CTX). This can also be observed in the UMAP where cluster 2 connects the HIP to CTX. Clusters 0, 5, 6, 7 and 11 form the CTX which can be further divided into cortical subplate and olfactory areas (6, 7) and the isocortex (0, 5, 11). Other examples include: hypothalamus (HY) – cluster 3; fiber tracts – cluster 4; thalamus (TH) – clusters 1 and 12; striatum (STR) – cluster 8; lateral ventricle (VL) – cluster 10. Cluster 9 maps mostly to the edges of the CTX and is enriched for genes expressed primarily in vascular tissue.

(C) Heatmap of differentially expressed genes within each cluster, represented by normalized and scaled expression values. Three selected genes per cluster with a high significance (adjusted p-value < 0.01) are highlighted for each cluster on the right side of the heatmap.

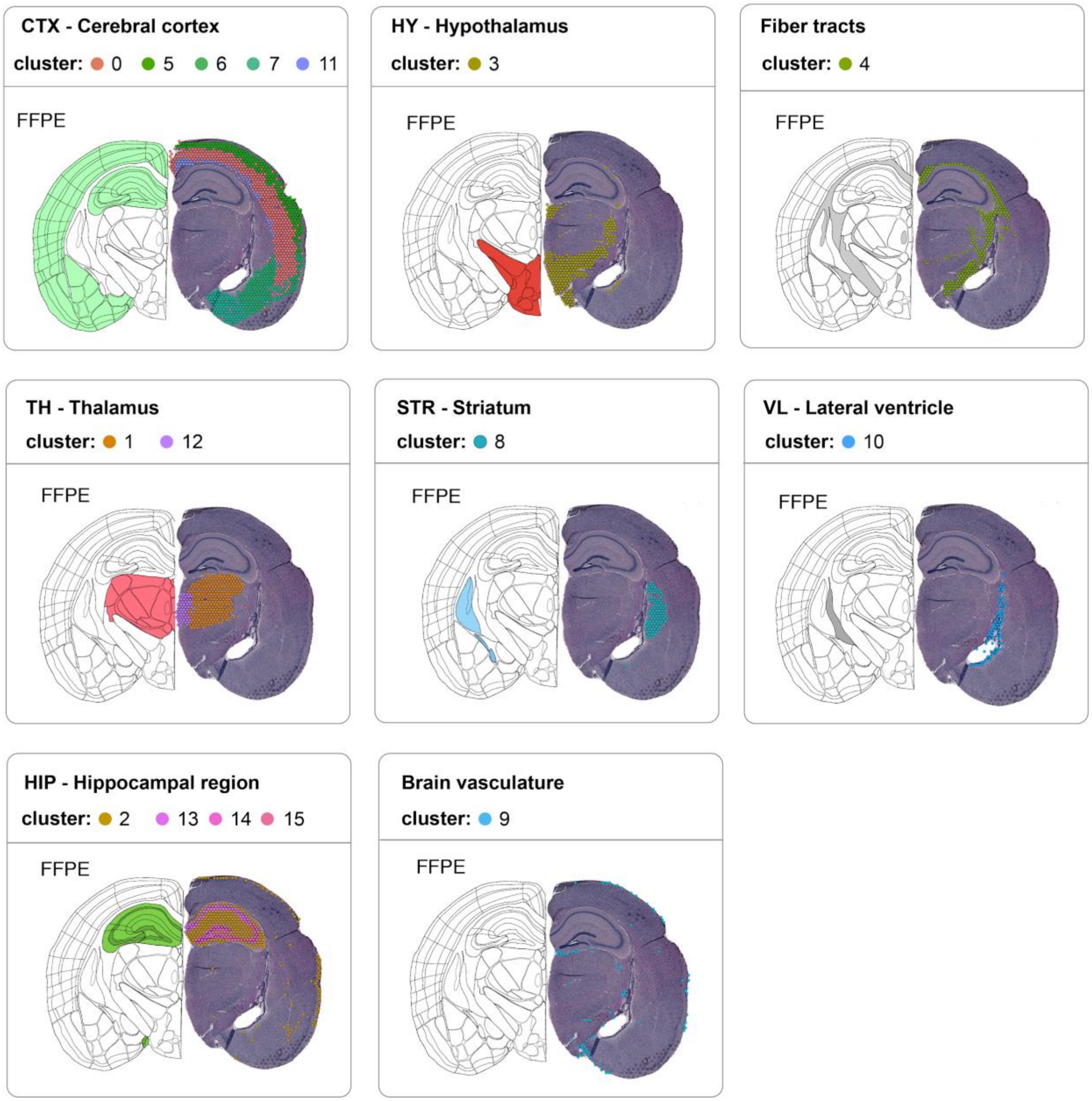

Figure S6. FFPE coronal section clusters agreement with anatomical regions, related to Figure 2 and STAR Methods.

Clusters have been grouped together based on their co-localization with anatomical landmarks from the Allen Mouse Brain Atlas.

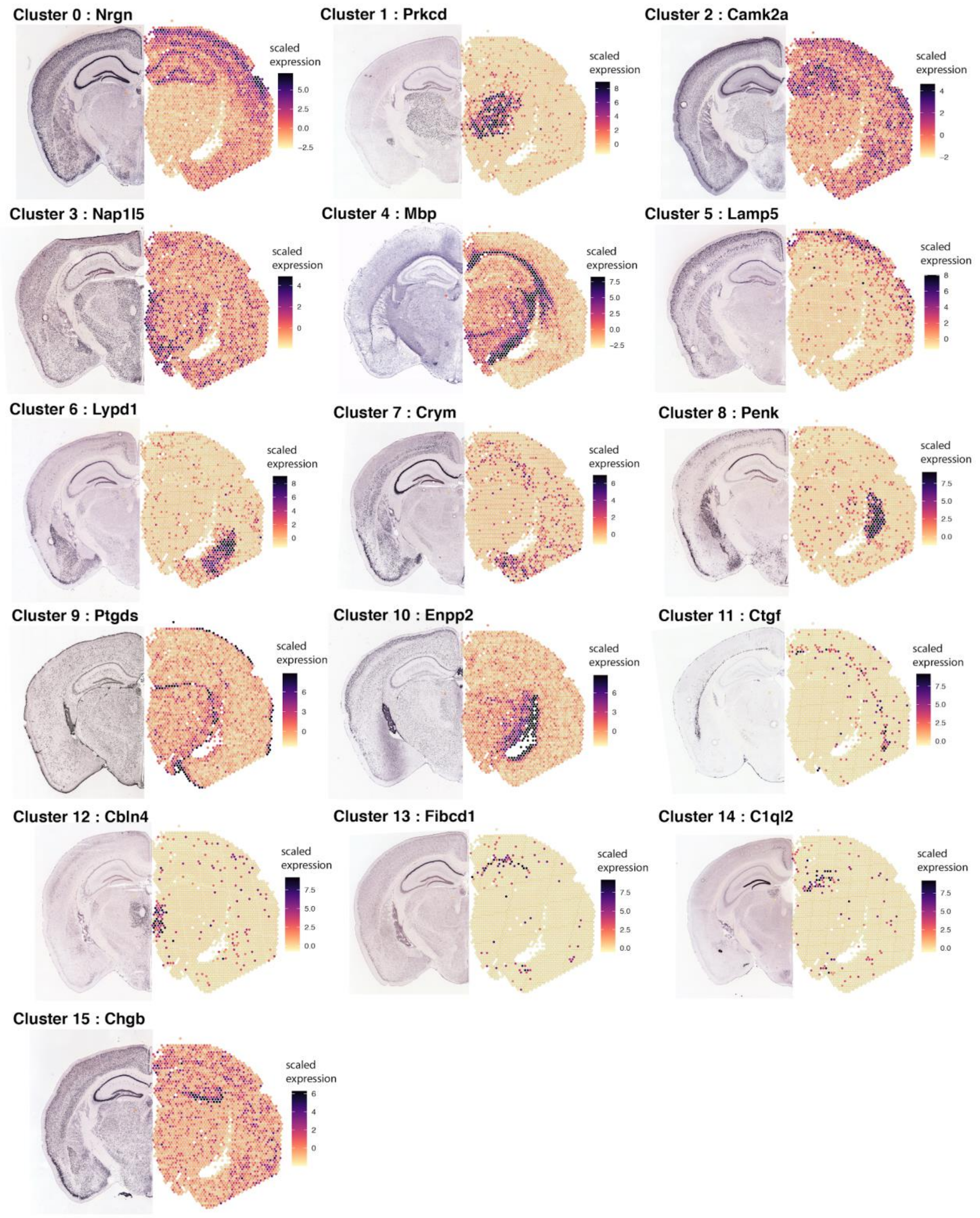
Cluster 0 : Nrgn
scaled expression
Cluster 1 : Prkcd
scaled expression
Cluster 2 : Camk2a
scaled expression
Cluster 3 : Nap1l5
scaled expression
Cluster 4 : Mbp
scaled expression
Cluster 5 : Lamp5
scaled expression
Cluster 6 : Lypd1
scaled expression
Cluster 7 : Crym
scaled expression
Cluster 8 : Penk
scaled expression
Cluster 9 : Ptgds
scaled expression
Cluster 10 : Enpp2
scaled expression
Cluster 11 : Ctgf
scaled expression
Cluster 12 : Cbln4
scaled expression
Cluster 13 : Fibcd1
scaled expression
Cluster 14 : C1ql2
scaled expression
Cluster 15 : Chgb
scaled expression

Figure S7. Validation of marker genes by ISH, related to Figure 2 and STAR Methods. Selected marker genes visualized by *in situ* hybridization (ISH) from the Allen Mouse Brain Atlas (left hemisphere) versus scaled expression values (scaled pearson residuals) from our FFPE data (right hemisphere). Marker genes were selected based on significance (adjusted p-values) and average log-fold change from the differential expression analysis.

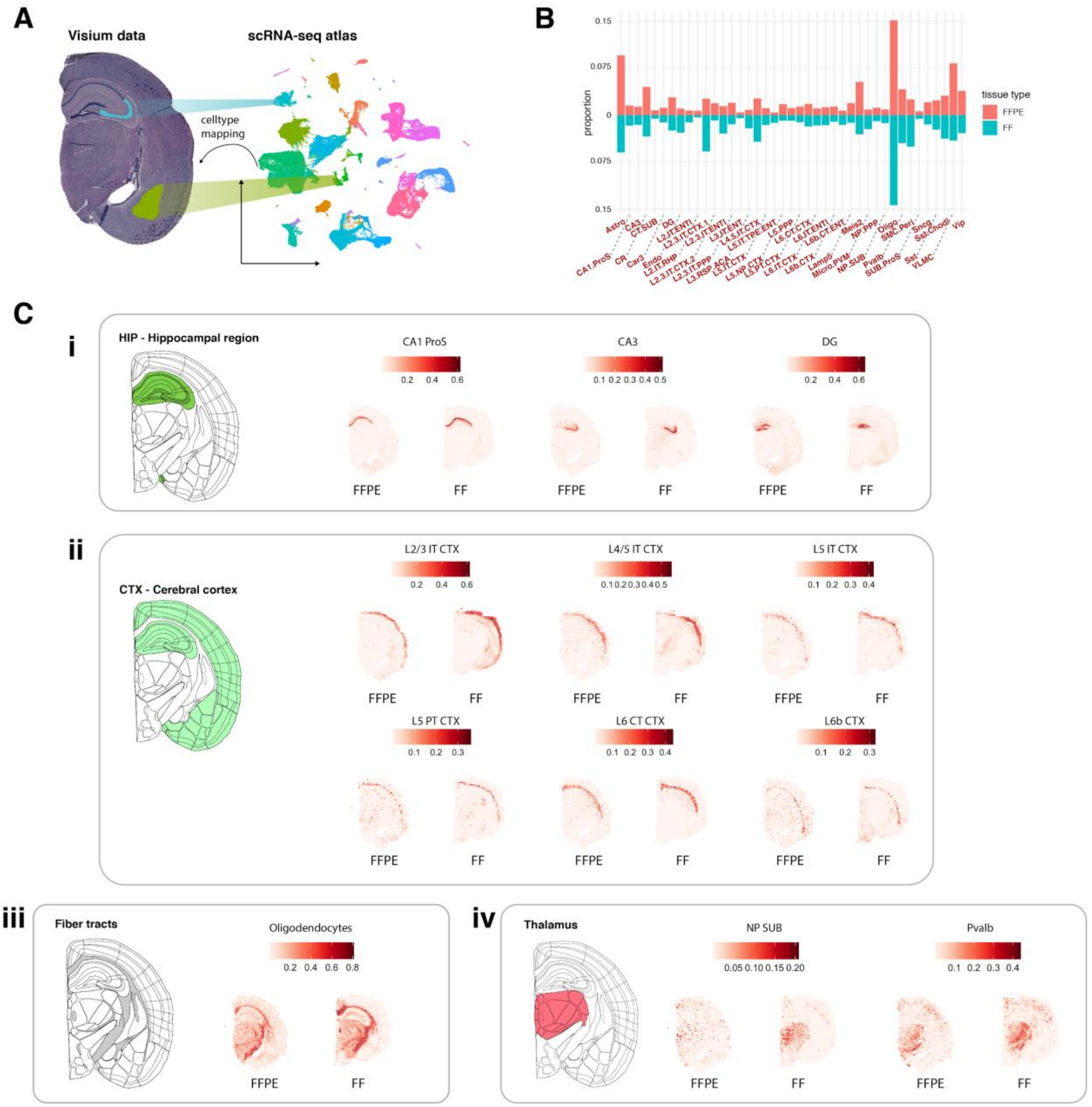

Figure S8. Cell type subclass mapping with stereoscope, related to Figure 2 and STAR Methods.

(**A**) Illustration of cell type mapping using spatially resolved transcriptomics data from a FFPE and FF coronal mouse brain section (left) and a SMART-seq scRNA-seq data from the Allen Brain Atlas (right), with a total of 41 subclasses. Cell type proportions are estimated from the mixed gene expression profiles of each spot in the spatially resolved transcriptomics dataset and can be visualized on the H&E image to define the spatial location of each cell type. **(B)** Summed proportion estimates for the FFPE and FF datasets. Red bars correspond to the summed cell type

proportions in the FFPE data and blue bars correspond to the summed cell type proportions in the FF data. Cell type subclass abbreviations are listed on the x axis. **(C)** A selected subset of cell types visualized by their relative proportions which co-localize with the hippocampal region (**i**), cerebral cortex (**ii**), fiber tracts (**iii**) and the Thalamus (**iv**). Color bars represent cell type proportions. **i)** Three distinct cell types co-localized with the HIP; CA1 ProS mapped to field 1 of the pyramidal layer (CA1-sp); CA3 mapped to field 3 of the pyramidal layer (CA3-sp) and DG mapped to the dentate gyrus (DG-sg). **ii)** Six cell types mapped to layers of the CTX. **iii)** Oligodendrocytes mapped to the fiber tracts. **iv)** Pvalv (parvalbumin) subclass and near-projecting subiculum (NP-SUB) subclass mapped to the thalamus.

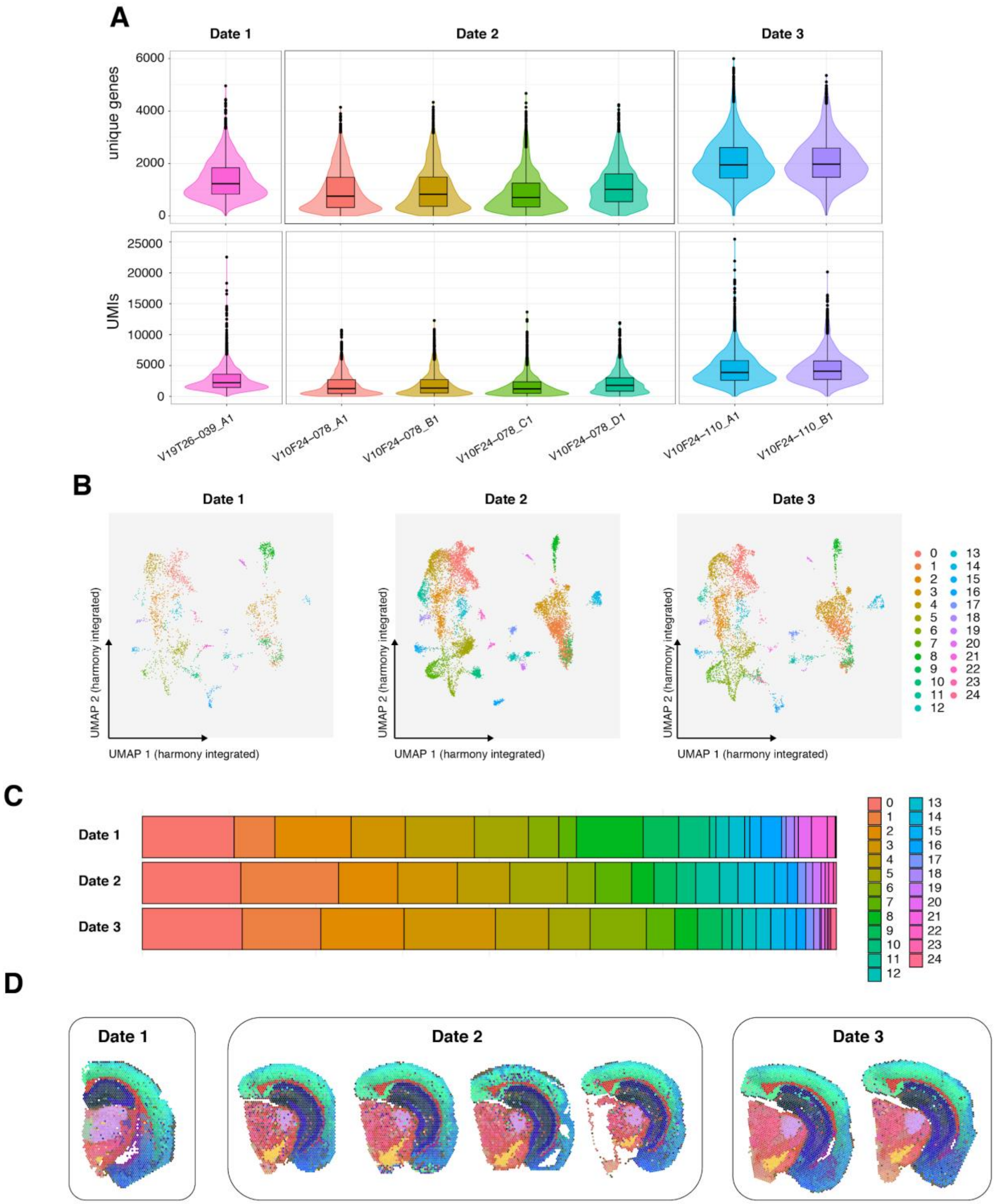

Figure S9. Spatial transcriptomics across multiple coronal mouse brain tissue sections, related to Figure 2 and STAR Methods.

(A) Quality metrics summarized for 7 coronal mouse brain datasets obtained at three different time points; unique genes (top panel) per spot and number of UMIs (bottom panel) per spot.

(B) UMAP embedding and clustering results (datasets integrated with harmony) of spot expression profiles split by time point.

(C) relative composition of clusters in each time point.

(D) Spatial visualization of expression profiles encoded as RGB colors.

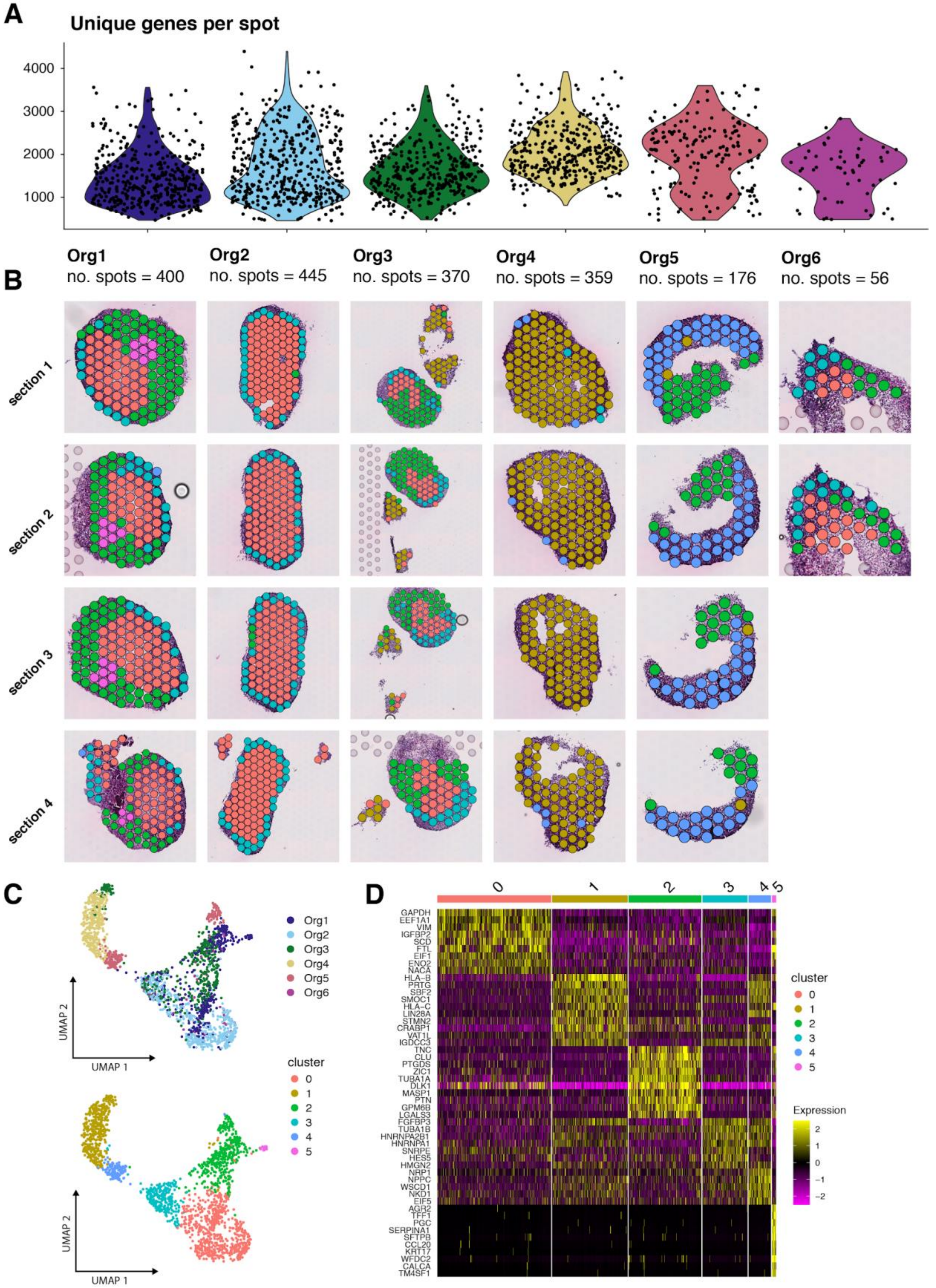
A
Unique genes per spot
4000
3000
2000
1000
B
Org1
no. spots = 400
Org2
no. spots = 445
Org3
no. spots = 370
Org4
no. spots = 359
Org5
no. spots = 176
Org6
no. spots = 56
section 1
section 2
section 3
section 4
C
Org1
Org2
Org3
Org4
Org5
Org6
UMAP 2
UMAP 1
cluster
0
1
2
3
4
5
D
GAPDH
EEF1A1
VIM
IGFBP2
SCD
FTL
EIF1
ENO2
NACA
HLA−B
PRTG
SBF2
SMOC1
HLA−C
LIN28A
STMN2
CRABP1
VAT1L
IGDCC3
TNC
CLU
PTGDS
ZIC1
TUBA1A
DLK1
MASP1
PTN
GPM6B
LGALS3
FGFBP3
TUBA1B
HNRNPA2B1
HNRNPA1
SNRPE
HES5
HMGN2
NRP1
NPPC
WSCD1
NKD1
EIF5
AGR2
TFF1
PGC
SERPINA1
SFTPB
CCL20
KRT17
WFDC2
CALCA
TM4SF1
Expression
2
1
0
−1
−2

Figure S10. Lung organoids overview, related to STAR Methods.

(A) Violin plot of unique genes per spot distributions across 6 lung organoids.

(B) Clusters overlaid on H&E images of replicate tissue sections for each of the 6 organoids (4 replicate sections for organoids 1-5 and 2 replicate sections for organoid 6).

(C) UMAP embedding of spot transcriptomic data colored by organoid (top) and clusters (bottom).

(D) DE heatmap showing the top 10 markers per cluster.

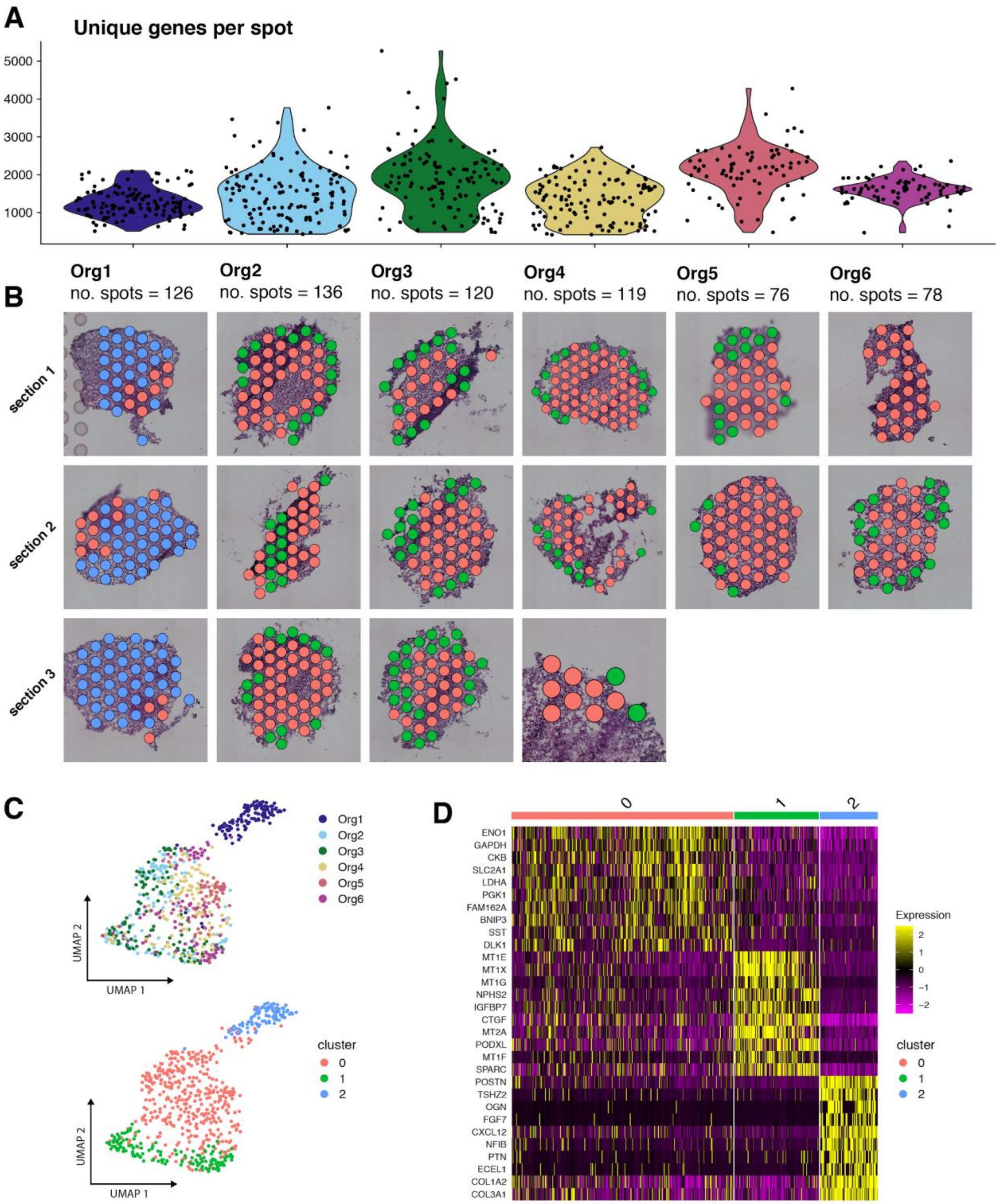

Figure S11. Kidney organoids overview, related to STAR Methods.

(A) Unique genes per spot distributions across kidney 6 organoids.

(**B)** Clusters overlaid on H&E images of replicate tissue sections for each of the 6 organoids (3 replicate directions for organoids 1-4 and 2 replicate sections for organoids 5-6).

(**C)** UMAP embedding of spot transcriptomic data colored by organoid (top) and clusters (bottom).
(D) DE heatmap showing the top 10 markers per cluster.

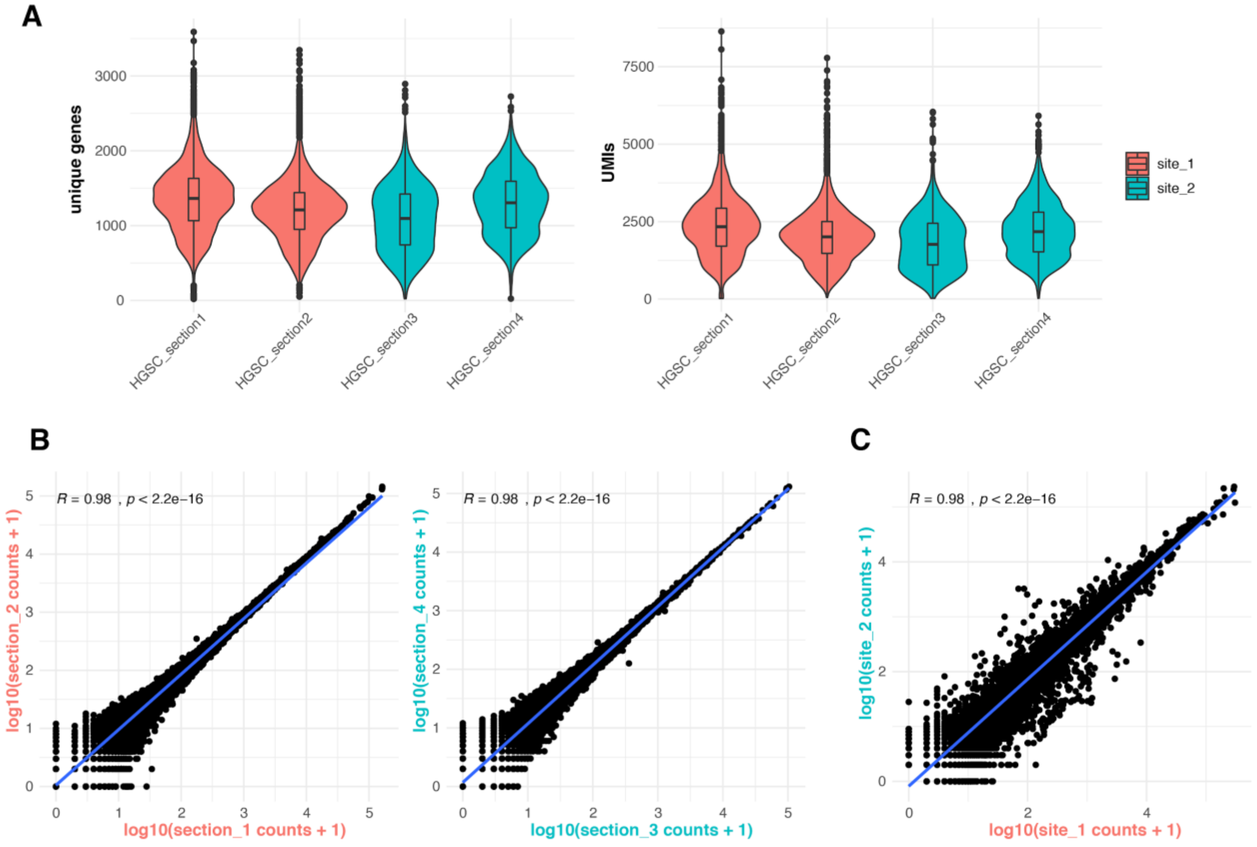

Figure S12. Ovarian carcinosarcoma data, related to Figure 3 and STAR Methods.

(A) Violin plots representing the number of genes per tissue-covered spot and the number of UMIs per tissue-covered spot respectively. Plots with similar color are technical replicates from the same FFPE block

(B) Gene-gene correlation plots between adjacent sections corresponding to each FFPE block showing R = 0.98 in both, indicating low technical variance.

(C) Gene-gene correlation plot between the two FFPE blocks.

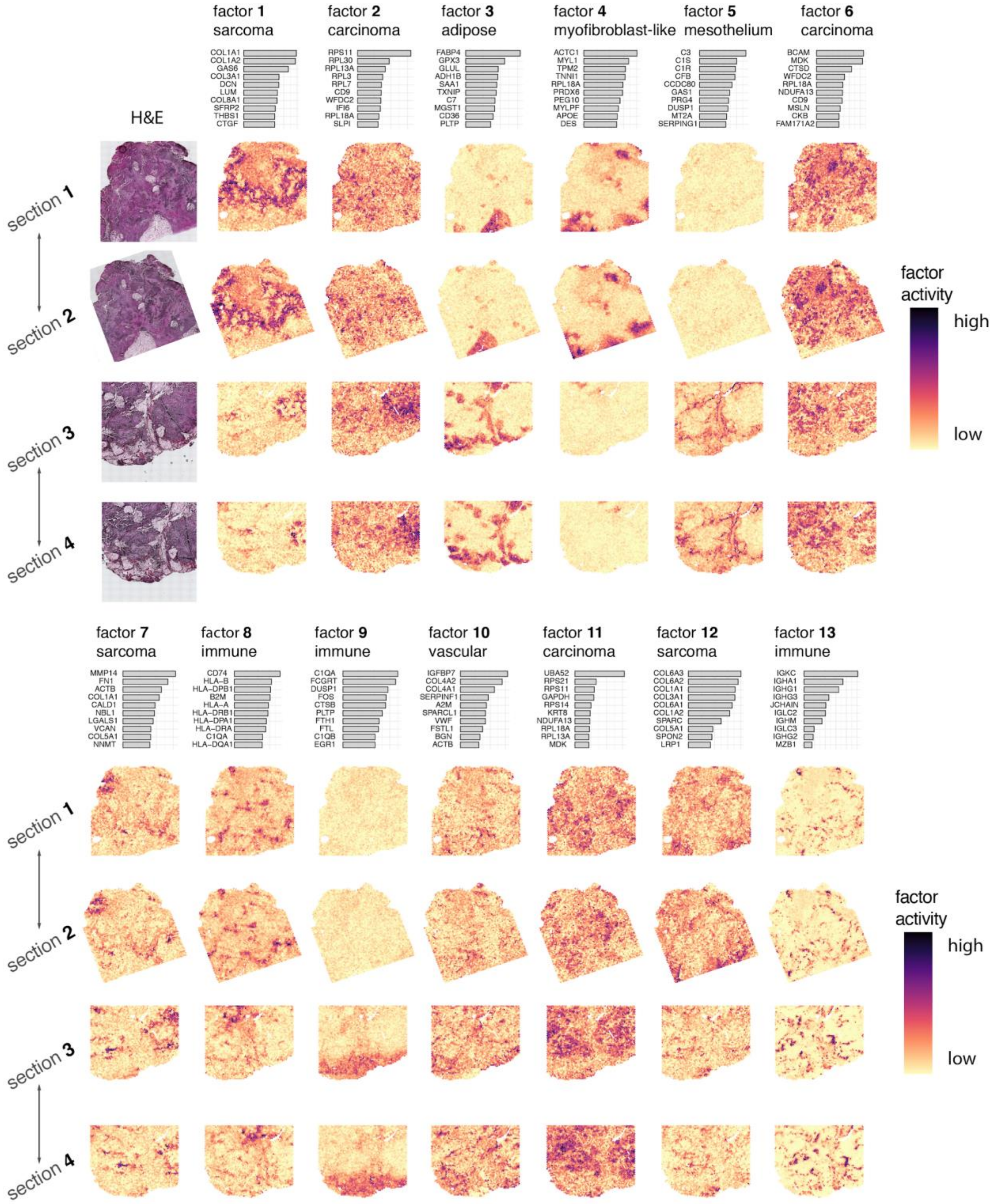

Figure S13. Spatial factor activity maps of four carcinosarcoma tissue sections, related to Figure 3 and STAR Methods.

The first column shows the H&E images linked to each spatial activity map. The remaining columns represent factor activity maps and consist of four rows, with one row for each tissue section. Adjacent sections are indicated by double headed arrows. The bar charts on top of each activity map shows the top 10 contributing genes for each factor. Factors 14 and 15 were driven mainly by ribosomal protein coding genes and were therefore omitted.

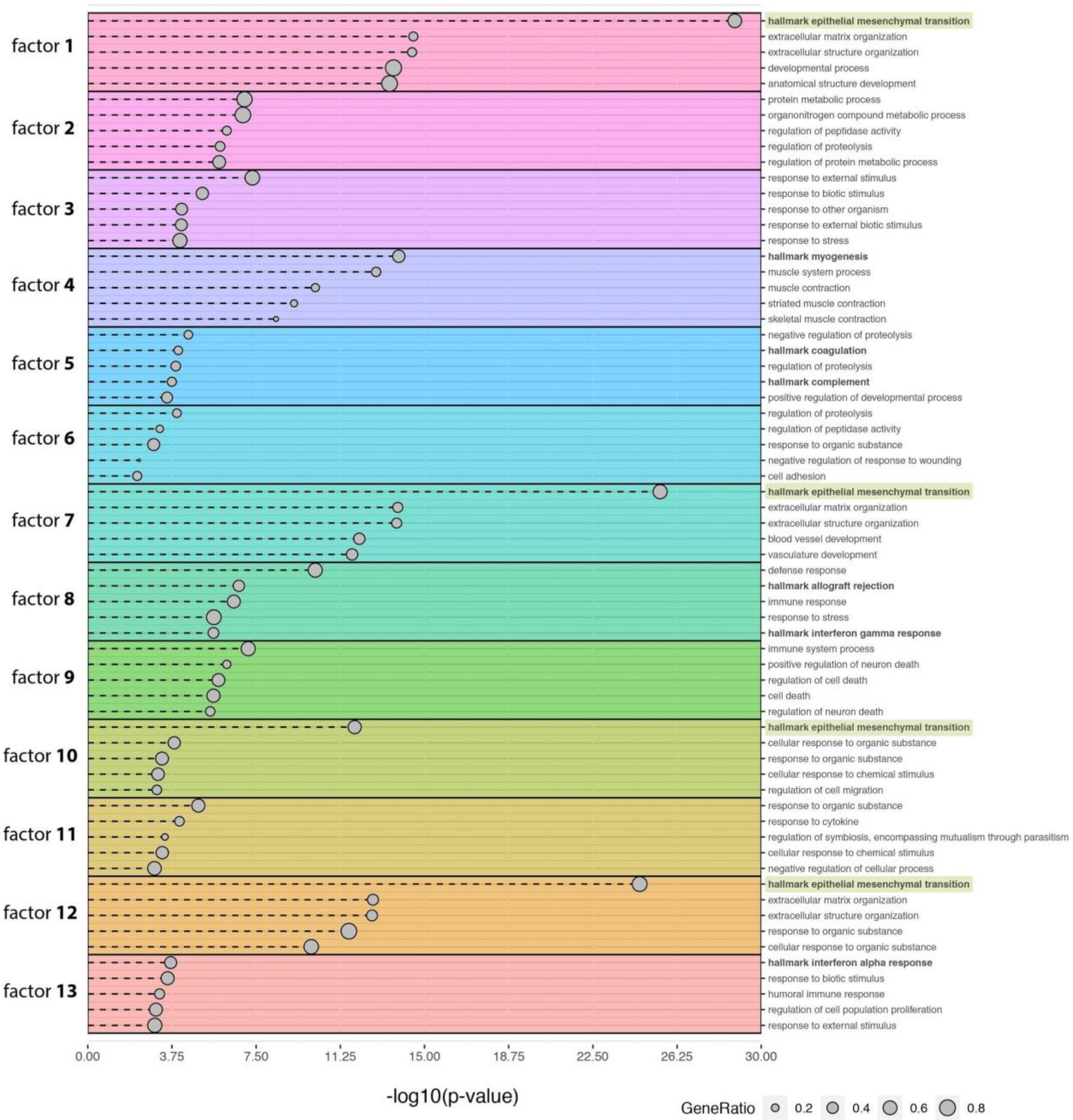

Figure S14. Pathway analysis of factors in carcinosarcoma tissue, related to Figure 3 and STAR Methods.

Top 5 most significant pathways per factor based on gene ontology (biological processes) and the cancer hallmark gene set collection from MsigDB. Terms from the hallmark gene set collection are highlighted with bold text with the epithelial to mesenchymal transition (EMT) term highlighted with green boxes. The GeneRatio is defined as the number of genes shared between the top

contributing genes per factor and the term gene set divided by the total number of top contributing genes.

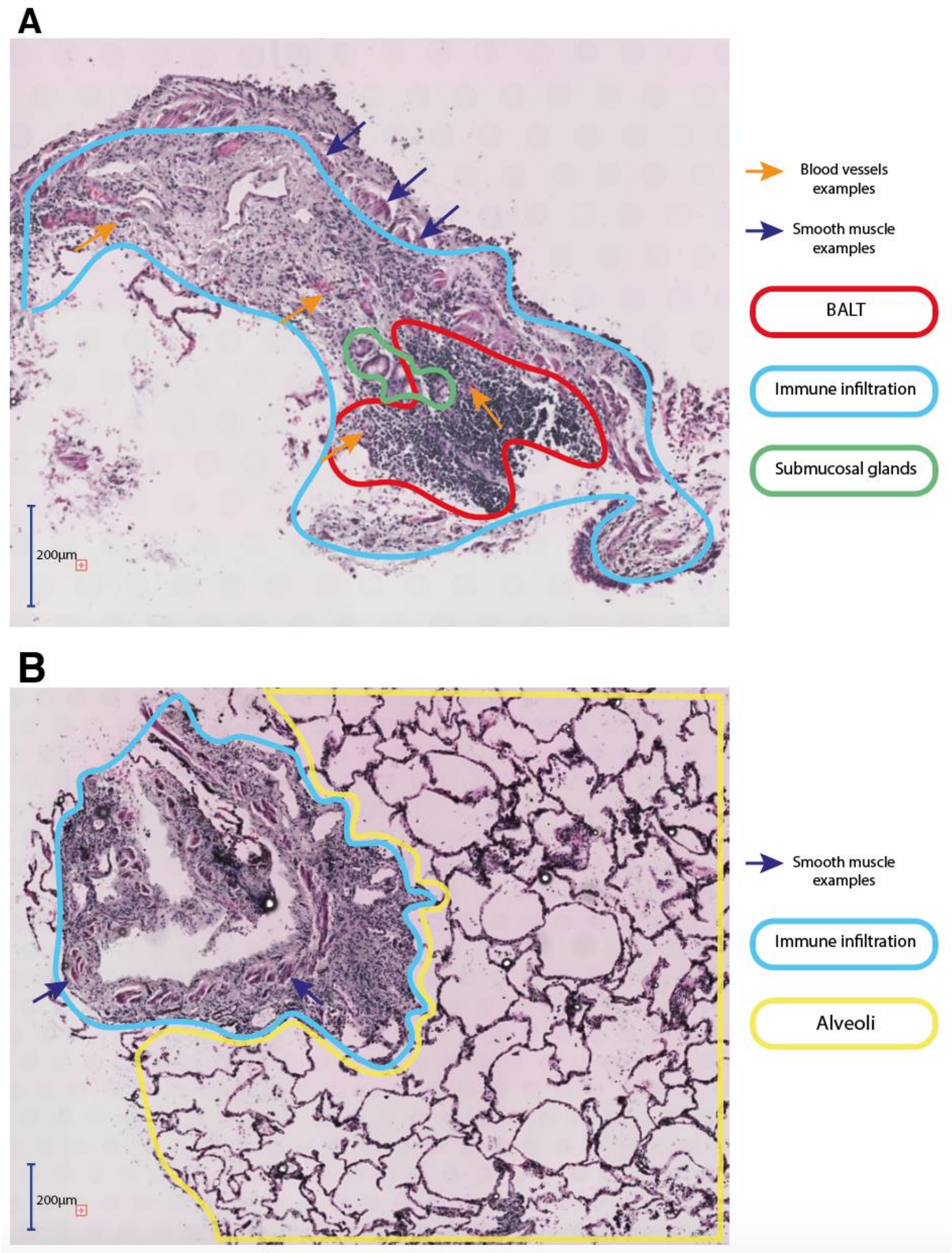
A
200µm
Blood vessels examples
Smooth muscle examples
BALT
Immune infiltration
Submucosal glands
B
200µm
Smooth muscle examples
Immune infiltration
Alveoli

Figure S15. Annotation of H&E stained Lung from Covid-19 patient biopsy, related to Figure 4 and STAR Methods.

(A) A fragment from bronchus with morphological damage (most likely a technical artifact not associated with the disease). Bronchus Associated Lymphoid Tissue (BALT) is observed, a few submucosal glands, vessels and a densely packed lymphoid follicle are found in the BALT area.

(**B**) Alveolar region and partial bronchioles with damaged morphology presenting extensive lymphocytic infiltration around the area.

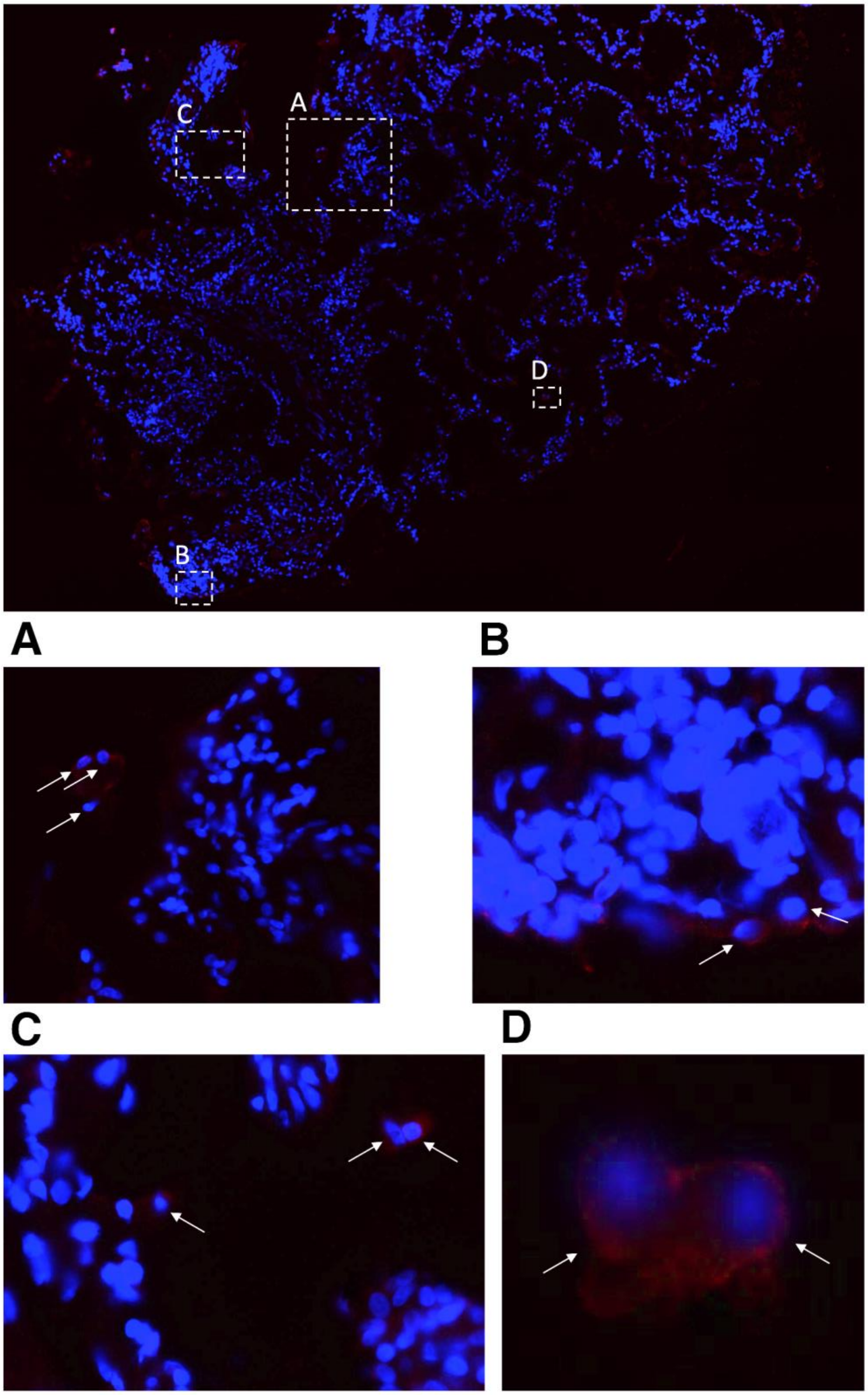
C
A
D
B
A
B
C
D

Figure S16. Immunofluorescent staining of SARS-CoV-1 spike protein, related to Figure 4 and STAR Methods.

Background has been removed by altering color balance (see methods).

Zoomed in areas (A)-(D), Examples of positive cells (red) marked by white arrows.

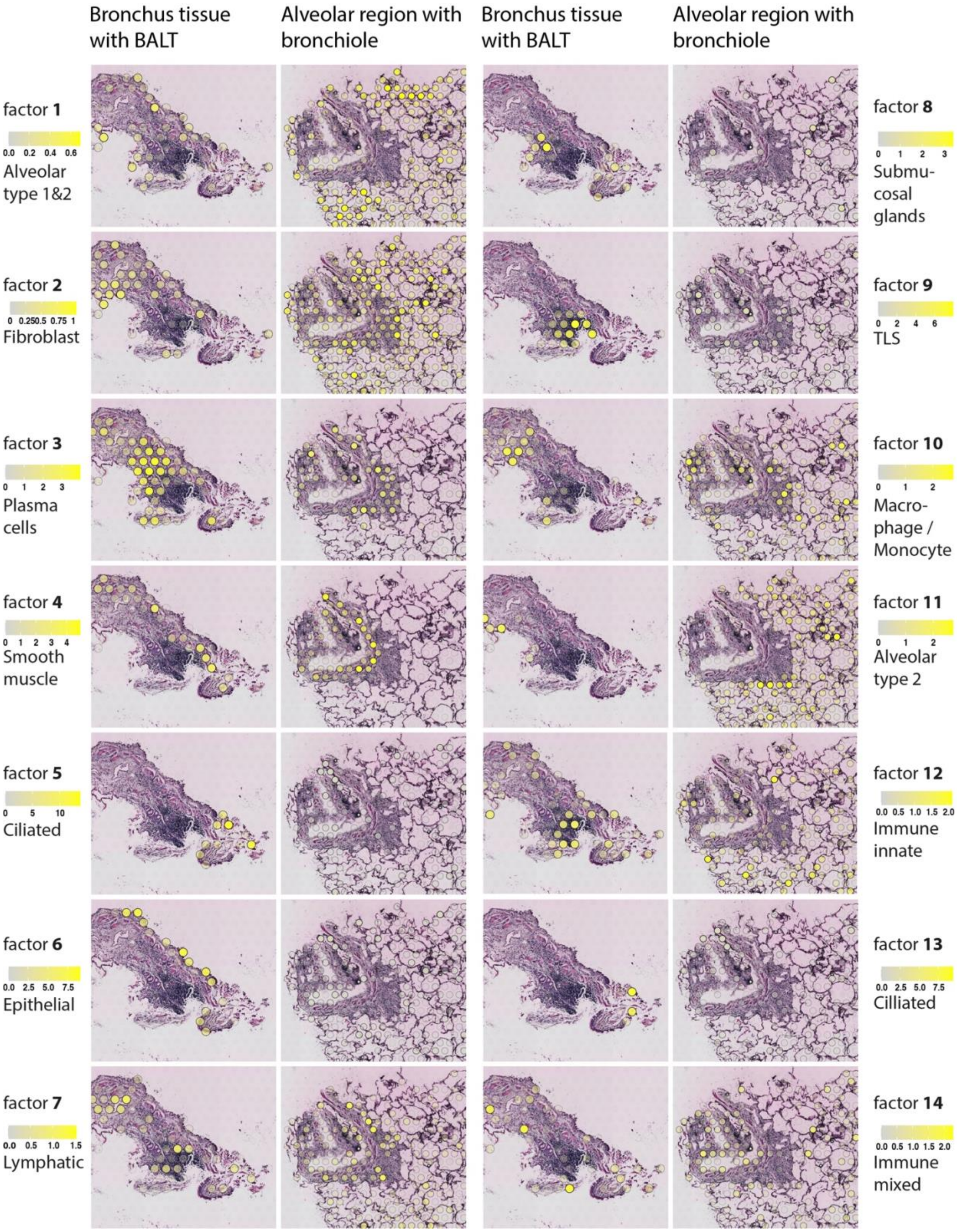

Figure S17. Spatial factor activity maps overlaid on H&E images of lung from COVID-19 PCR-positive patient, related to Figure 4 and STAR Methods.

Two main tissue regions are shown; bronchus tissue with a BALT structure and an alveolar region with a bronchiole. Each factor is visualized as a spatial activity map (yellow dots) and has been scaled to make the spots gradually opaque, i.e. spots with a value of 0 are 100% transparent while spots with a maximum factor activity value are opaque.

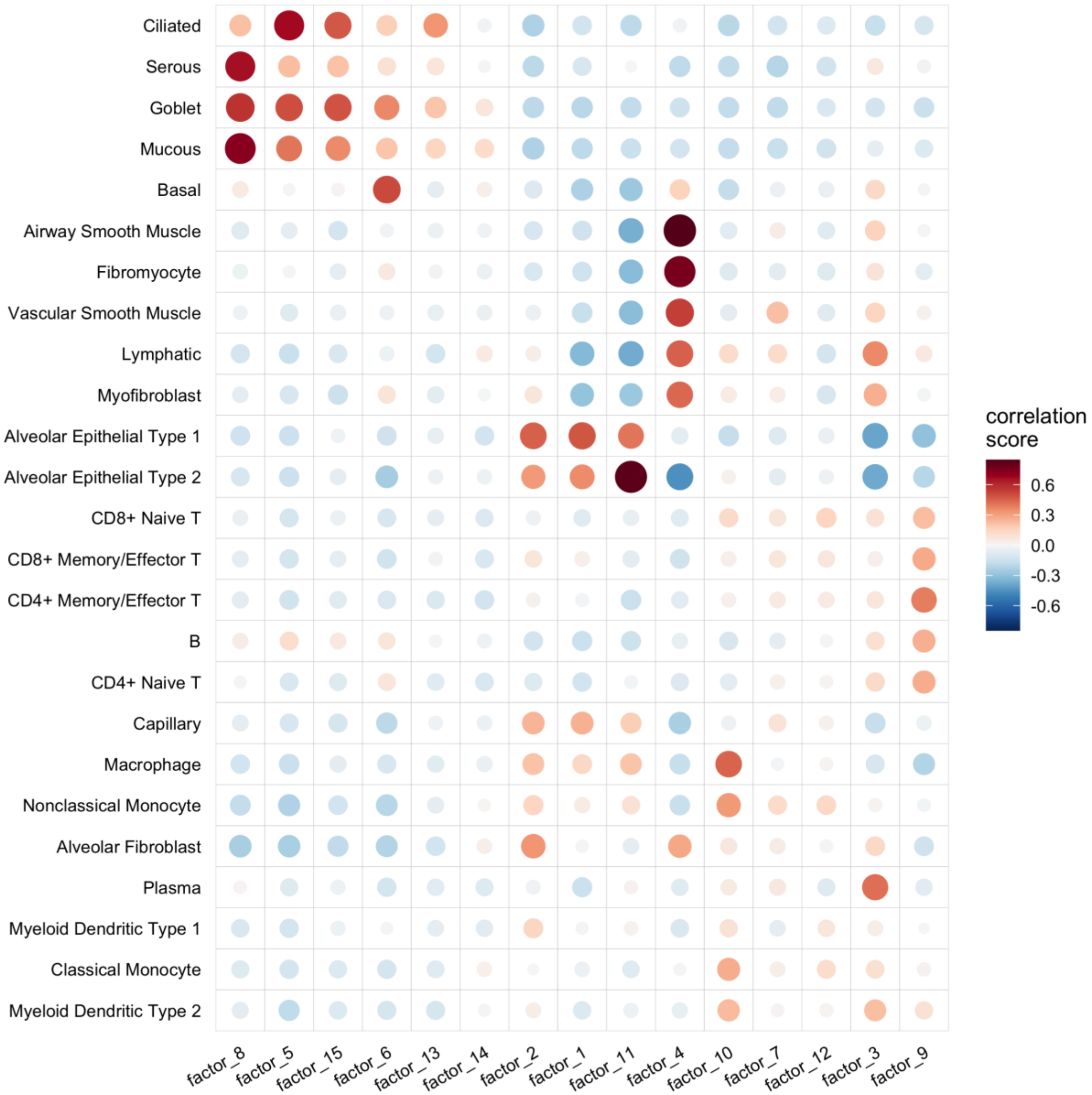

Figure S18. Correlation between cell type enrichment scores and factors in lung tissue infected with Covid-19, related to Figure 4 and STAR Methods.

The x axis represents 25 cell types from a molecular cell atlas of the human lung. The y axis represents factors computed using Non-negative Matrix Factorization of the spatially resolved gene expression data. The color and size of each dot represents the strength of the Pearson correlation coefficient between log-transformed cell type enrichment scores (AUC) and log-transformed factor activity scores.

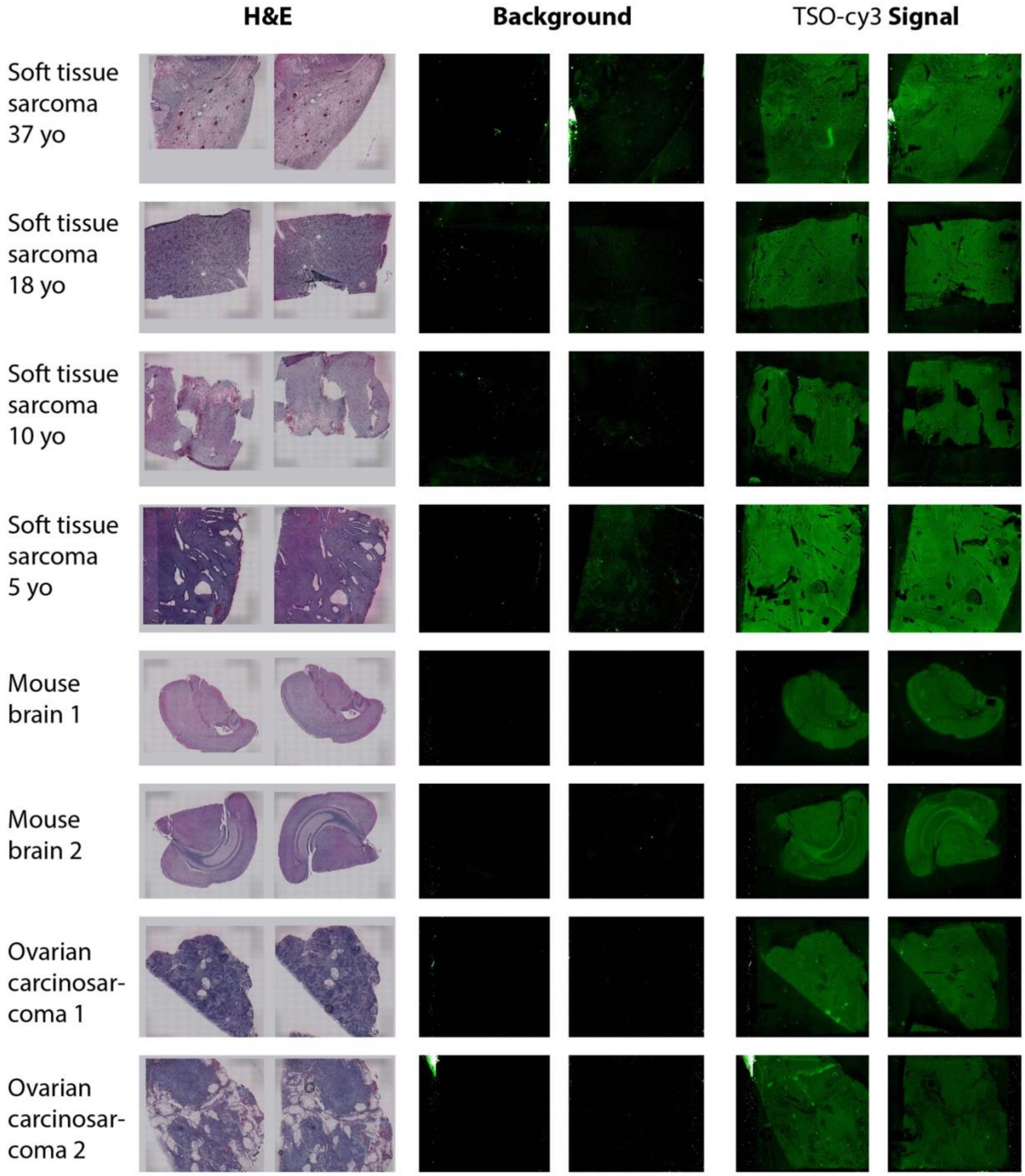

Supplemental Figure 19 | cy3-TSO QC assay applied on different FFPE tissue types, related to STAR Methods.

Each row shows duplicate sections collected from a different tissue block. H&E images of the duplicate tissue section are shown in the left column. During reverse transcription of mRNA, a

cDNA strand is synthesized and a complementary to TSO sequence added in the 3´end. Then, the tissue is removed and the mRNA templates are de-hybridized. Slides are imaged to ensure there is no tissue left over the slide. Fluorescence scans of the negative control/autofluorescence after enzymatic tissue removal, i.e. without cy3-TSO added, are shown in the middle column (background). Finally, a fluorescent cy3-TSOprobe is hybridized and the cDNA signal image is taken. Fluorescence scans of the cy3-TSO cDNA footprints are shown in the right column (TSO-Cy3-signal).

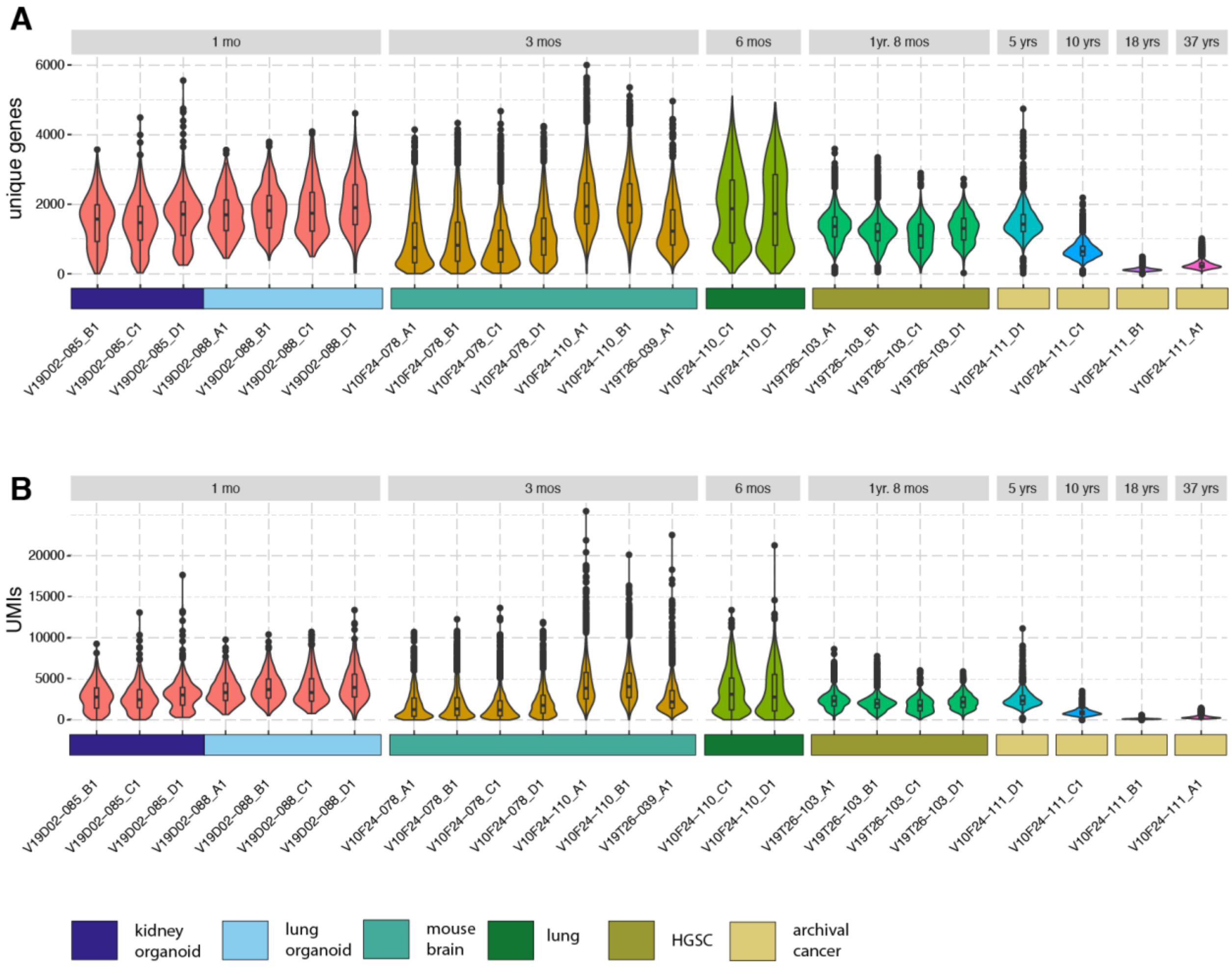

Figure S20 | Quality metrics by storage time, related to STAR Methods.

Quality metrics for 6 tissue types stored between 1 month and 37 years

(A) Unique genes per spot distributions.

(B) UMIs per spot distributions.
