# Supplementary material for: Genome-wide spatial expression profiling in formalin-fixed tissues
Source: Cell Genom. 2021 Dec 8;1(3):100065. doi: 10.1016/j.xgen.2021.100065 (PMC9903805; doi:10.1016/j.xgen.2021.100065)
Supplement: Document S2. Article plus supplemental information [file mmc9.pdf]

Cell Genomics

Technology

Genome-wide spatial expression profiling in formalin-fixed tissues

Graphical abstract

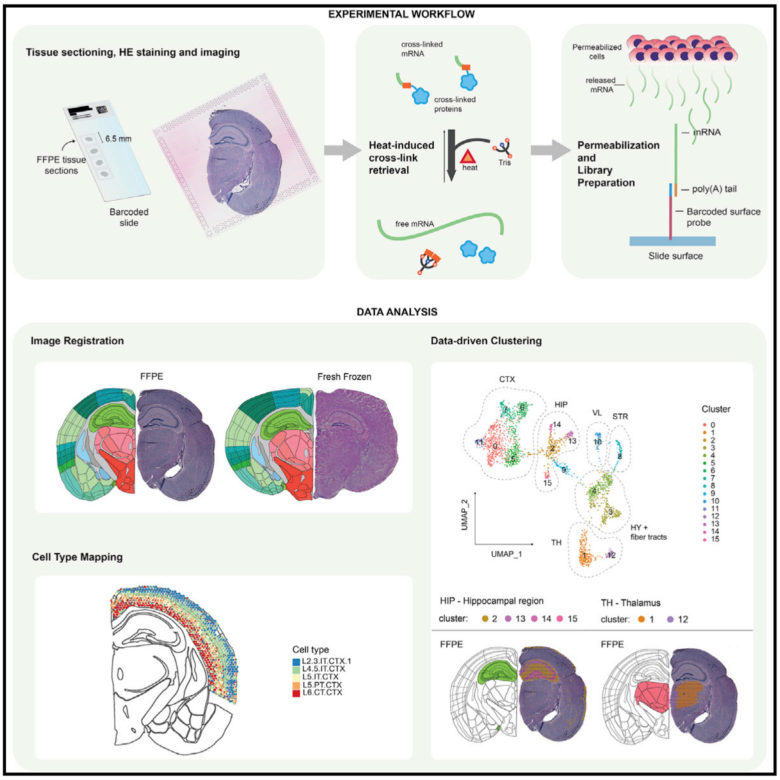

Highlights

- Decrosslinking of formalin-fixed tissues enables transcriptome-wide mRNA analysis
- Molecular profiling of FFPE mouse brain data recapitulates known anatomical structures
- Molecular signatures are associated with histopahological features in ovarian cancer
- Analysis of SARS-CoV-2-infected lung reveals post-infection immunological responses

Authors

Eva Gracia Villacampa, Ludvig Larsson, Reza Mirzazadeh, ..., Ali Mirazimi, Joseph Carlson, Joakim Lundeberg

Correspondence

joakim.lundeberg@scilifelab.se

In brief

Gracia Villacampa et al. develop a method to perform spatial analysis of mRNA in FFPE- and PFA-fixed tissue sections using spatially barcoded slides and based on oligo(dT) mRNA capture.

Check for updates

Gracia Villacampa et al., 2021, Cell Genomics *1*, 100065
December 8, 2021 © 2021 The Authors.
https://doi.org/10.1016/j.xgen.2021.100065

CellPress

Cell Genomics

CellPress
OPEN ACCESS

Technology

Genome-wide spatial expression profiling in formalin-fixed tissues

Eva Gracia Villacampa,[1,13] Ludvig Larsson,[1,13] Reza Mirzazadeh,[1,14] Linda Kvastad,[1,14] Alma Andersson,[1] Annelie Mollbrink,[1] Georgia Kokaraki,[2] Vanessa Monteil,[3] Niklas Schultz,[2,4] Karin Sofia Appelberg,[5] Nuria Montserrat,[6,7] Haibo Zhang,[8] Josef M. Penninger,[9,10] Wolfgang Miesbach,[11] Ali Mirazimi,[3,5,12] Joseph Carlson,[2] and Joakim Lundeberg[1,15,*]

[1]Department of Gene Technology, School of Engineering Sciences in Chemistry, Biotechnology and Health, KTH Royal Institute of Technology, Science for Life Laboratory, 17165 Stockholm, Sweden
[2]Department of Oncology-Pathology, Karolinska Institute, 17176 Stockholm, Sweden
[3]Department of Laboratory Medicine, Unit of Clinical Microbiology Karolinska Institutet and Karolinska University Hospital, 17177 Stockholm, Sweden
[4]Department of Pathology and Cytology, Karolinska University Hospital, 17176 Stockholm, Sweden
[5]Public Health Agency of Sweden, 17182, Solna, Sweden
[6]Pluripotency for Organ Regeneration, Institute for Bioengineering of Catalonia (IBEC), The Barcelona Institute of Technology (BIST), Barcelona, Spain
[7]Catalan Institution for Research and Advanced Studies (ICREA), Barcelona, Spain
[8]Departments of Anesthesia, Medicine, and Physiology, University of Toronto, Keenan Research Centre for Biomedical Science, Unity Health Toronto, Toronto, ON, Canada
[9]Department of Medical Genetics, Life Sciences Institute, University of British Columbia, V6T 1Z3 Vancouver, BC, Canada
[10]Institute of Molecular Biotechnology of the Austrian Academy of Sciences, Dr. Bohr-Gasse 3, 1030 Vienna, Austria
[11]Department of Haemostaseology and Haemophilia Center, Institute of Transfusion Medicine, Medical Clinic 2, University Hospital Frankfurt, 60590 Frankfurt am Main, Germany
[12]National Veterinary Institute, 751 89 Uppsala, Sweden
[13]These authors contributed equally
[14]These authors contributed equally
[15]Lead contact
*Correspondence: joakim.lundeberg@scilifelab.se
https://doi.org/10.1016/j.xgen.2021.100065

SUMMARY

Formalin-fixed paraffin embedding (FFPE) is the most widespread long-term tissue preservation approach. Here, we report a procedure to perform genome-wide spatial analysis of mRNA in FFPE-fixed tissue sections, using well-established, commercially available methods for imaging and spatial barcoding using slides spotted with barcoded oligo(dT) probes to capture the 3′ end of mRNA molecules in tissue sections. We applied this method for expression profiling and cell type mapping in coronal sections from the mouse brain to demonstrate the method's capability to delineate anatomical regions from a molecular perspective. We also profiled the spatial composition of transcriptomic signatures in two ovarian carcinosarcoma samples, exemplifying the method's potential to elucidate molecular mechanisms in heterogeneous clinical samples. Finally, we demonstrate the applicability of the assay to characterize human lung and kidney organoids and a human lung biopsy specimen infected with SARS-CoV-2. We anticipate that genome-wide spatial gene expression profiling in FFPE biospecimens will be used for retrospective analysis of biobank samples, which will facilitate longitudinal studies of biological processes and biomarker discovery.

INTRODUCTION

For decades, formalin-fixing and paraffin embedding (FFPE) has been the preferred method for tissue preservation of clinical biospecimens. FFPE is less expensive and easier to use than freezing-based methods and offers a high degree of preservation of morphological detail.[1] As a consequence, there are vast numbers of FFPE specimens in biobanks readily available for genomics research, which could be used for extensive longitudinal studies on large patient cohorts. However, formalin fixation negatively affects nucleic acid integrity and accessibility due to formalin-mediated strand cleavage and the formation of cross-linked adducts between RNA and other biomolecules.[2]

Lately, several methods have been developed for spatial analysis of tissue sections (recently reviewed by Asp et al.[3]) and can broadly be characterized into: (1) hybridization-based approaches, which require pre-existing knowledge of the targets for probe design and (2) sequencing-based approaches, which

This is an open access article under the CC BY license (http://creativecommons.org/licenses/by/4.0/).

allow for unbiased mRNA poly(A) capture in the tissue being analyzed spatially. One of the pioneering methods for sequencing-based analysis was spatial transcriptomics,[4] which has repeatedly demonstrated its value when applied to fresh frozen (FF) tissue sections for the exploration and profiling of transcriptomic landscapes in organ development and disease.[5–8] Today, there are commercially available platforms for spatial transcriptomics, including the Visium platform from 10x Genomics.

The development of methods for sensitive sequencing-based spatial transcriptomics of FFPE samples[9,10] has been hampered by the technical challenges caused by the formalin-induced cross-linking and degradation of mRNA molecules. Genome-wide quantification strategies have been developed for application on bulk FFPE samples,[11,12] which typically involve ribosomal depletion or targeted capture using oligonucleotide probe hybridization for enrichment of fragmented mRNA; however, there are currently no methods available for unbiased spatial mRNA-profiling of FFPE tissue samples. In this study, we present a protocol based on commercially available platforms, adapted to recover spatially resolved mRNA profiles from FFPE tissue sections. We apply this protocol to characterize the transcriptomic landscape of FFPE tissues from mouse brain, human organoids, clinical cancer samples, and lung tissue from a patient infected with severe acute respiratory syndrome (SARS)-CoV-2. Our results suggest that the proposed protocol is versatile and can recover genome-wide information from distinct tissue types preserved under variable conditions. FFPE has been the gold standard for storage of clinical samples in biobanks, and we anticipate that our findings will promote research on archived biospecimens to better characterize the molecular underpinnings of key events in disease progression, immunological responses, and organ development.

DESIGN

Recovery of fixed mRNA for spatial FFPE analysis is achieved by removing paraffin and cross-links *in situ* with the tissue section placed on a barcoded slide (Figure 1). The protocol used with FFPE samples differs from the established FF workflow[13] in several aspects to allow for cross-link reversal as well as changes to the size selection and fragmentation process. Certain steps were introduced to adjust to the particularities of FFPE samples, while other changes pertained to data-yield optimization (see STAR Methods).

First, floating FFPE tissue sections were attached to a spatially barcoded slide, dried in an oven, and then deparaffinized by successive immersions in xylene and ethanol, a deparaffinization strategy commonly used in immunohistochemistry (IHC). Given that FFPE tissues are already fixed with a cross-linking agent for an extended period of time (approximately 24 h), we reasoned that the morphology would be preserved and therefore omitted the fixation step. Tissue sections were stained with hematoxylin and eosin (H&E) and imaged under a high-resolution microscope. Next, the tissue sections were pre-permeabilized with collagenase followed by cross-link reversal. Collagenase was included in early spatial transcriptomics protocols to boost tissue removal and mRNA capture since it is known to aid in the disruption of the extracellular matrix structure.[14]

Cross-link reversal was performed by heat-induced retrieval[15] at 70°C with Tris-EDTA buffer at pH 8.0. A pH of 8.0 has been previously reported to prevent unwanted side reactions, such as pH-dependent RNA hydrolysis.[16] Moreover, a quenching mechanism has been proposed between Tris and formaldehyde molecules.[2] Decrosslinking was optimized by investigating variations of the Tris-EDTA (TE) buffer composition and pH using colon cancer FFPE tissue stored at 6°C (Figure S1). We found that none of the compositions presented any drastic variation in fluorescently labeled cDNA signals and concluded that all compositions yielded an acceptable signal. The two conditions that we deemed to have slightly better cDNA signals were evaluated through an additional tissue optimization (TO) experiment (Figure S2A). Next, in order to obtain quantitative data, cDNA libraries were prepared using these conditions. Once again, the sequencing data did not show any major differences, with the seemingly best composition being TE buffer pH 8.0 (Table S1).

After cross-link reversal, the tissue sections were enzymatically permeabilized. It is recommended to optimize permeabilization times based on the tissue type, since specific features that may affect mRNA accessibility vary across tissues. This is usually determined through a TO assay, as described elsewhere.[17] In our experiment, we additionally evaluated PBS as a possible decrosslinking agent (Figure S2B). In mouse brain tissue, the optimal signal was determined at a permeabilization duration of 30 min (Figure S2B), and PBS did not outperform TE buffer pH 8.0.

Next, sequencing libraries from different tissues were prepared, as described in STAR Methods, with experimental conditions and quality metrics given in Table S2.

TSO-based quality control (QC) assay

Within this work, we have developed an assay to evaluate the spatial accessibility of non-crosslinked polyadenylated RNA molecules of a sample prior to library preparation (Figure S3). In the beginning of our method design process, we relied on the RNA integrity number (RIN) and distribution value 200 (DV200) calaculated for total RNA extractions to gauge whether an FFPE block had sufficient RNA quality to be analyzed. However, these metrics held less predictive power of sequencing data quality than desired. A few discrepancies were identified: one set of clinical carcinosarcoma samples had relatively low RIN values (observed: 2.3–2.5, preferred: >4.0; see STAR Methods) but still provided good transcriptome coverage upon sequencing, while another set with similar RIN values rendered sequencing data with high duplicate content (Table S3). The first FFPE mouse brain sample we studied, from which we obtained high transcriptome coverage for our analysis, had a DV200 value of 65%. Nevertheless, an FFPE block with a DV200 of as high as 68%, which was later shown to have been fixed for an extremely long time (5 days, usually $\leq$ 24 h), resulted in a library of low complexity (Table S3). These quality measurements are either based on ribosomal RNA integrity or total RNA and do not account for the possible loss of poly(A) tail of mRNA caused by formaldehyde treatment. Additionally, these are in-bulk quality

CellPress
OPEN ACCESS

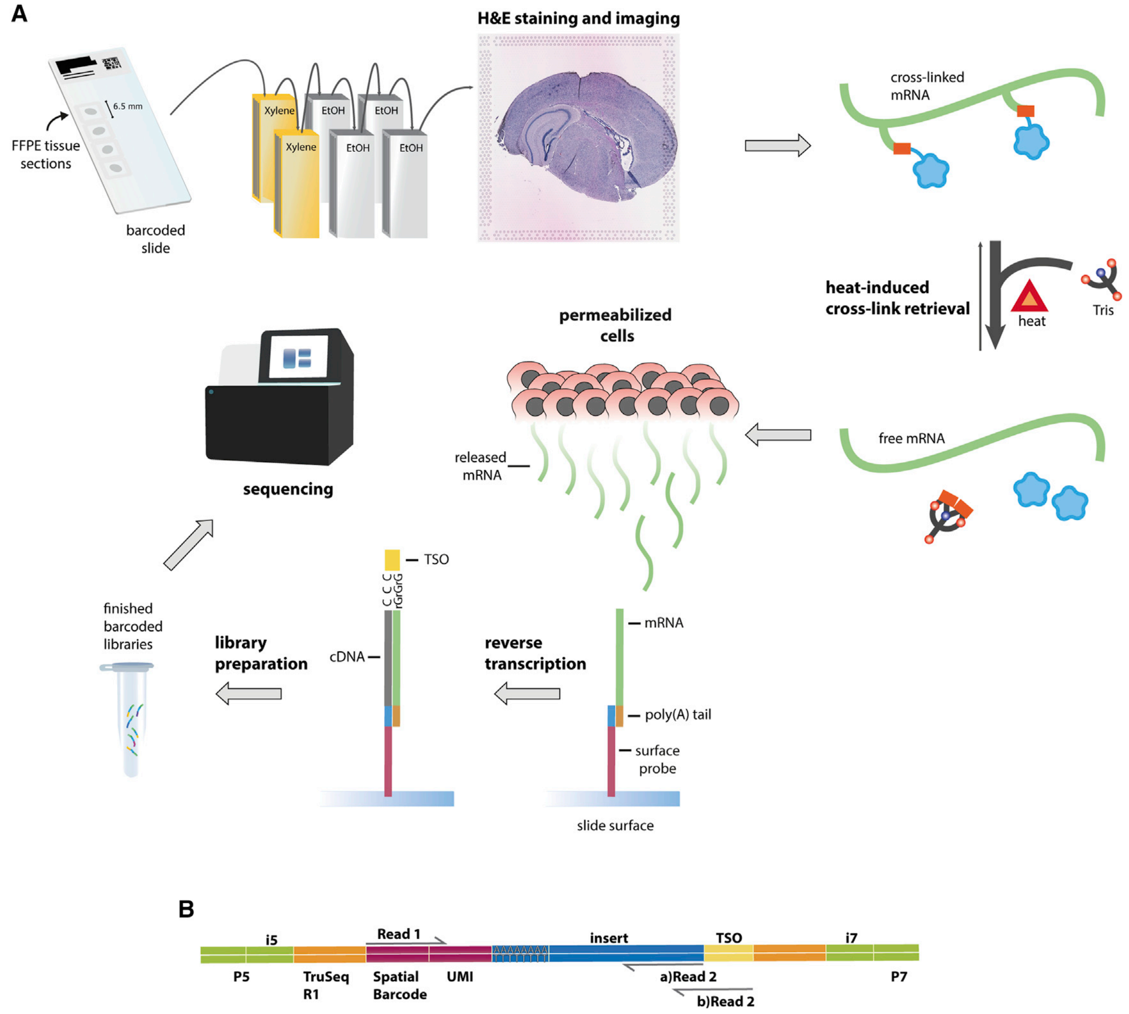

Figure 1. Visualization of mRNA expression across FFPE tissue sections with adapted protocol

(A) Tissue sections are placed on spatial gene expression slides. The slides are deparaffinized following a standard protocol (see STAR Methods). H&E-stained sections are imaged under a high-resolution microscope. Cross-links are reversed by heat and in the presence of Tris-EDTA buffer at pH 8.0. Tissues are permeabilized so that mRNA diffuses to the barcoded surface probes and hybridizes with its oligo(dT) capture region. A cDNA strand is synthesized by reverse transcription. Finished libraries are sequenced either with custom primer for read 2 reverse complementary to template switch oligo (TSO) sequence or with conventional Truseq R2 primer.

(B) Final construct overview. Read 2 can begin in 2 different positions depending on whether the sequencing has been performed utilizing a custom primer (see STAR Methods).

assessment methods that fail to account for spatial variance in mRNA abundance, accessibility, and degradation. Performing TO assays, where the fluorescent signal corresponds to the amount and length of cDNA strands synthesized by reverse transcription, directly accounts for these.

We hypothesized that, in the case of FFPE samples, the presence of cross-links in mRNA strands could potentially obstruct elongation of cDNA in the reverse transcription step and consequently hinder the addition of the template-switch oligo (TSO) sequence. Therefore, cDNA signals observed in a TO assay do not necessarily reflect the quantity of amplifiable fragments for FFPE library preparation. Another common consideration of TO assays is the difficulty removing tissue from the array surface. Tissue leftovers produce autofluorescence, and since reverse transcription occurs prior to tissue removal in a standard TO protocol, there is no possibility to scan a separate background image for tissue autofluorescence. Therefore, to confirm the absence of tissue-generated autofluorescence, the slide should be examined under a light microscope for the presence of cellular debris.

To address these issues, we designed a QC assay to only target amplifiable cDNA, i.e., cDNA molecules with a TSO present (Figure S3). A fluorescently labeled DNA oligo was designed to hybridize with the TSO sequence on surface-bound cDNA, where the fluorescence signal serves as a proxy to detect amplifiable fragments. This assay, hereafter referred to as the TSO-based QC assay, was used to assess the spatial accessibility of RNA of a subset of FFPE blocks.

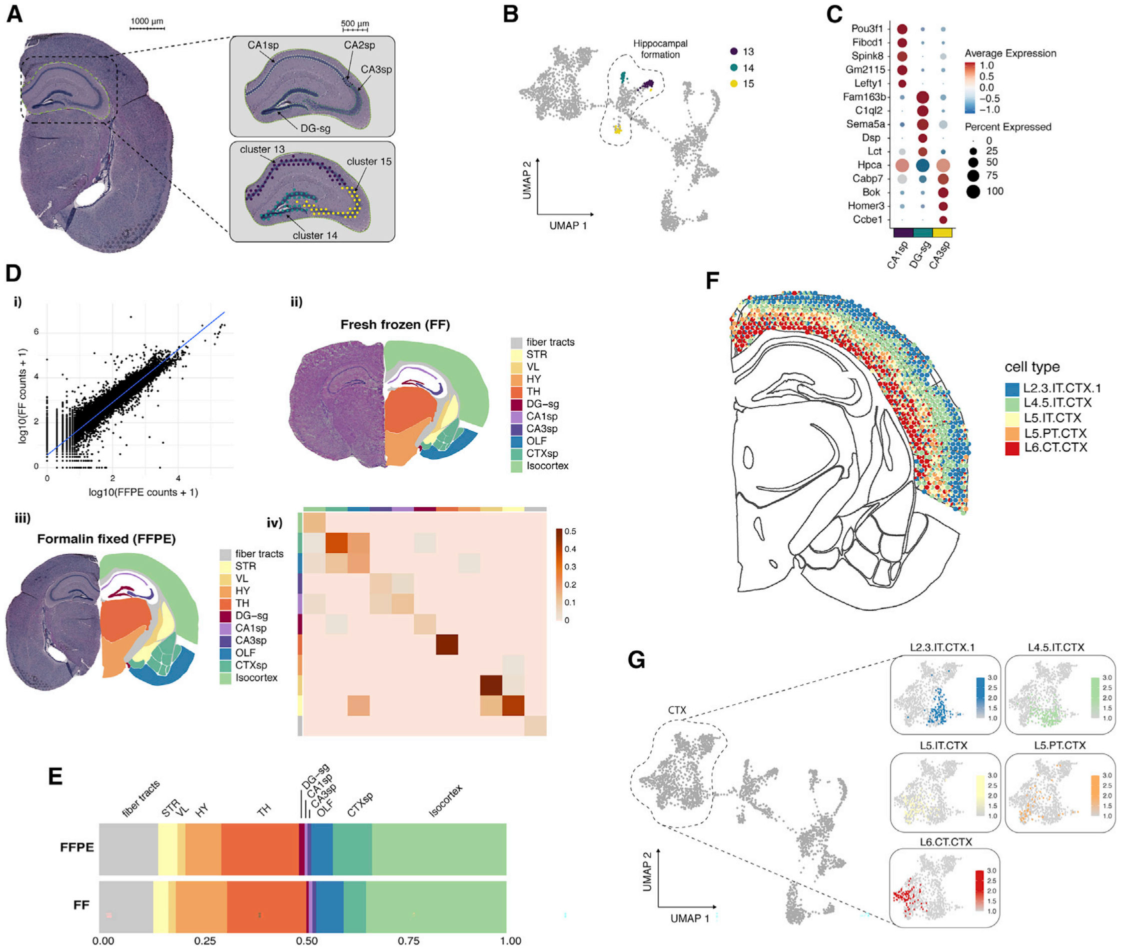

Figure 2. Analysis of FFPE mouse brain tissue

(A) H&E image of a coronal section with the hippocampal region highlighted (left). Zoomed-in view of the hippocampal region (right). 3 anatomical structures are highlighted (top right): field 1 pyramidal layer (CA1sp), field 3 pyramidal layer (CA3sp), and the dentate gyrus (DG-sg). 3 selected clusters (13, 14, and 15) from the unsupervised analysis (bottom right) exemplify the correspondence between structures defined by molecular features and neuroanatomy.

(B) UMAP embedding with clusters 13, 14, and 15 shown in (A) highlighted.

(C) Dot plot showing the top 5 most significant marker genes for each hippocampal cluster in (A) and (B).

(D) (i) Gene-gene scatterplot displaying log10-transformed gene counts between FFPE data and a dataset obtained from fresh frozen tissue (Pearson correlation score of 0.95 with a p value of 2.2×10^{-16}). (ii and iii) H&E image of a coronal mouse brain tissue section manually registered to the Allen Brain Atlas. (ii) fresh frozen and (iii) FFPE tissue, respectively. The right lobe represents a map of 11 anatomical regions defined by the Allen Brain Atlas. (iv) Heatmap displaying the concordance between the FFPE and fresh frozen datasets computed for each of the 11 anatomical regions.

(E) Composition of spots across anatomical regions displayed as relative proportions.

(F) 5 neuronal cell type subclasses from the isocortex mapped to the FFPE coronal section using the cell type mapping method *stereoscope*. Each spot is illustrated by a pie chart showing the relative composition of the 5 cell type subclasses. Cell type subclass abbreviations: L2/3 IT CTX 1, cerebral cortex layer 2/3 intratelencephalic; L4/5 IT CTX, cerebral cortex layer 4/5 intratelencephalic; L5 IT CTX, cerebral cortex layer 5 intratelencephalic; L5 PT CTX, cerebral cortex layer 5 pyramidal tract; L6 CT CTX, cerebral cortex layer 6 corticothalamic.

(G) Visualization of proportion values for the 5 cell type subclasses (same as in F) shown in UMAP space. All 5 cell types were enriched in clusters localized in the cerebral cortex (CTX).

RESULTS

FFPE spatial transcriptomics data recapitulate anatomical structures of the mouse brain

The mouse brain has been extensively characterized using multiple genomics methods coupled with detailed neuroanatomy maps in various projects. Such efforts make the mouse brain a suitable model tissue to evaluate and explore the potential of spatial transcriptomics applied to FFPE tissue. Anatomical structures of the mouse brain have been annotated in detail based on histological features from H&E-stained tissue sections in the coronal reference of the Allen Brain Atlas,[18,19] where 132 tissue sections, collected at every 100 μm, span the whole mouse brain along the anterior-posterior axis. Using this anatomical reference, we could determine the position of collected tissue sections along the anterior-posterior axis of

the mouse brain and map out anatomical structures based on histology. Moreover, we reasoned that a comparison between spatial transcriptomics data obtained from FFPE tissue and data obtained from tissue embedded using conventional methods, analyzing FF tissue, would be valuable to evaluate quality aspects of the data such as gene recovery and complexity. Therefore, we downloaded a publicly available spatial transcriptomics dataset obtained from FF mouse brain tissue sectioned in the coronal plane (available from 10x Genomics[20]) to be used as a reference. Next, we collected a coronal tissue section from an FFPE mouse brain at approximately the same distance along the anterior-posterior axis as the FF section by matching tissue histology. From this tissue section, we generated spatial transcriptomics data using our spatial FFPE protocol. The H&E images of the FF and FFPE coronal mouse brain tissue sections were registered to the coronal reference atlas using the *wholebrain* framework[21] (Figure S4A) to map out anatomical regions. At a sequencing depth of ~50 k reads per tissue-covered spot, a total of 2,533 barcoded capture locations (spots) were obtained, with an average of ~1,200 unique genes and ~2,200 unique molecules detected per spot from the FFPE tissue section (Table S2; Figure S4B). After normalization of the data, expression-based clustering identified a total of 16 clusters that clearly corresponded to established anatomical structures (Figures S5A–S5C). Clusters 2, 13, 14, and 15 formed the hippocampal (HIP) region (Figures S4A and S6), whereas clusters 13 and 15 mapped to fields 1 and 3 of the pyramidal layer of Ammon's horn and cluster 14 mapped to the granule cell layer of the dentate gyrus (DG-sg) (Figures 2A, 2B, and S6). The fine-grained mapping of these small structures exemplifies how FFPE spatial transcriptomics data can be used to, in an unbiased manner, resolve structures covered by only a few spots. Moreover, differential expression (DE) analysis showed upregulation of known marker genes *Fibcd1* and *Spink8* (excitatory neurons, hippocampus CA1) in the CA1sp region, *Cabp7* and *Bok* (excitatory neurons, hippocampus CA3) in the CA3sp region, and *C1ql2* (granule neurons, DG) in the DG-sg[22] (Figure 2C). Uniform manifold approximation and projection(UMAP) embedding of the FFPE data (Figure S5B) indicates that the spot transcription profiles cluster into structures that reflect larger anatomical regions of the mouse brain forming the cerebral cortex (CTX), hypothalamus (HY), fiber tracts, thalamus (TH), striatum (STR), lateral ventricle (VL), and the HIP region.

Next, we set out to explore the capability of our FFPE spatial transcriptomics dataset to define mRNA markers associated with anatomical regions. For each one of the 16 clusters, one representative marker gene was selected from the DE analysis (Table S4; Figure S5C), and its expression pattern was compared with published *in situ* hybridization (ISH)[18] images (Figure S7). The spatial distribution of marker expression values in the spatial transcriptomics data was in agreement with the ISH images, thus highlighting the potential to extract molecular landmarks in an unsupervised manner from FFPE material.

To systematically assess the quality of our FFPE-derived spatial transcriptome data, we compared our FFPE dataset with the publicly available FF dataset (2,698 spots) (Figure S4). At a sequencing depth ~115 k reads per tissue-covered spots, the FF dataset covered on average ~6,000 unique genes and ~27,200 unique molecules per spot (Figure S4B). Most of the captured molecules were annotated as protein coding in both datasets, although we did observe a higher relative abundance of large intergenic noncoding RNA (lincRNA), ribosomal protein coding genes, and mitochondrial protein coding genes in the FF dataset (Figure S4C). The Pearson r-score between the FFPE and FF datasets treated as bulk was 0.95 with a p value lower than 2.2×10^{-16} (Figures 2Di and S4D), indicating a strong correlation between the two data types at bulk level.

Moreover, we set out to measure the agreement between expression profiles recovered from the two datasets. We reasoned that although we could observe a substantial difference in capture efficiency between the two conditions, the gene expression signatures of anatomically defined regions should show high agreement at the genome-wide scale. To make this comparison, we used the histological information from the H&E images to define anatomical regions. The H&E images of the FFPE and FF tissue sections were first manually registered to the coronal reference atlas to generate a map of the brain anatomy using the *wholebrain* workflow. Based on the manual registration, spots were then grouped into 11 preselected anatomical regions (Figures 2Dii–2Diii), with comparable fractions of spots defined within each region (Figure 2E). Within each of the 11 regions, we computed enrichment scores for every gene by comparing the expression levels and detection rates relative to the background. These enrichment profiles were then used as a basis to compute a rank-biased overlap (rbo) between regions across the two datasets. A summary of overlap estimates is present in Figure 2Div, showing a high agreement in gene expression between matched anatomical regions between spatial transcriptomics data obtained from FFPE and FF tissue.

Several computational methods have been proposed to deconvolve the mixed transcription profiles of spots using single-cell RNA sequencing (scRNA-seq) data. Here, we used the probabilistic method *stereoscope*[23] to map out 41 different subclasses of cell types using a scRNA-seq SMART-seq dataset obtained from the Allen Mouse Brain Atlas initiative.[24] The single-cell mapping method allowed us to deconvolve the spot transcriptomes of our FFPE and FF datasets into spot-wise proportion estimates, meaning that the spatial distribution of each cell type could be assessed (Figure S8A). When looking at the total cell type proportions, i.e., the cell type proportions summed over all spots, we observed similar proportion estimates across all cell types in the two data types (Figure S8B).

To compare the distribution of cell types across the two data types, we visualized the estimated proportions for a subset of cell types that mapped to the HIP region, isocortex, and fiber tracts (Figure S8C). In the HIP region, we found a high enrichment of CA1, CA3, and DG cell types (Figure S8Ci), confirming our previous observations from the clustering analysis. 5 neuron cell type subclasses were clearly enriched in the isocortex compared to the other 10 anatomical regions (Figures 2F, 2G, and S8Cii), and the spatial distribution of these cell types recapitulate the layered organization previously described in literature.[25,26] In addition, we noted an enrichment of oligodendrocytes in the fiber tracts (Figure S8Ciii), where they play a role in myelination of longe-range fibers.[25] For a few cell types, we

observed a stronger signal in the FF dataset, especially for cell types mapping to the TH (Figure S8Civ). The most likely explanation for the increased signal strength is the higher mRNA capture efficiency in the FF data. However, we also expected to see some variability in cell type composition in the two tissue sections, as they were collected at slightly different distances along the anterior-posterior axis. In brief, we found an overall high agreement in the spatial distribution of cell type proportion estimates between FFPE and FF datasets despite a substantial difference in mRNA capture efficiency. These findings support the applicability of our protocol to generate spatially resolved transcriptomic data from FFPE tissues with sufficient complexity to detect cell-type-specific gene expression signatures. We have also created a public data browser for viewing all of the data generated in the current study (GitHub: https://github.com/ludvigla/FFPE_mouse_brain_explorer). Here, cell type proportion estimates for all 41 cell types can be visualized and compared between the two datasets.

Spatial transcriptomics methods have been successfully applied to a number of biological systems to uncover molecular processes that often require the collection of replicate tissue sections from multiple individuals to recover the full representation of the tissue heterogeneity. Therefore, to obtain a representative dataset of any tissue type, the reproducibility of the method is essential. We sought to address the reproducibility of our FFPE-adapted spatial transcriptomics protocol on replicate sections collected from different FFPE blocks. A total of 7 spatial transcriptomics libraries were obtained from coronal sections of FFPE mouse brain tissue, with an average of 900 to 2,077 unique genes per spot and an average of 1,208 to 4,064 unique molecular identifiers (UMIs) per spot (Table S2; Figure S9A). The libraries were obtained in three separate experiments, and we observed that the quality metrics showed little variation within each experimental batch but varied across batches. Such batch-specific effects are commonly observed in transcriptomics data as a consequence of, among other things, sample handling, tissue quality, variable sequencing depth, and variation of the experimental procedure. To combat these technical effects, we applied *harmony*,[27] a multi-dataset integration method developed to find a common embedding of gene expression profiles across multiple experimental batches. The *harmony* embedding was further compressed into a 2-dimensional representation using UMAP, followed by unsupervised clustering which generated 25 clusters (Figure S9B). Although the data quality differed substantially across the three batches, the 25 clusters could be recovered from each batch at comparable numbers (Figure S9C). Color coding of gene expression profiles (see STAR Methods) after integration with *harmony* highlighted similarities across gene expression profiles within the spatial domain across the 7 tissue sections (Figure S9D).

Spatially resolved transcriptomics delineates heterogeneity of paraformaldehyde-fixed organoids

Stem-cell-derived 3-dimensional culture systems known as organoids have emerged as an important model system to recreate the architecture and physiology of human organs *ex vivo* in striking detail.[28] These models are easy to manipulate and monitor over time, thus providing a valuable tool for researchers to study biological processes, toxicological effects of drugs, and the effects of perturbations. Some of the most prominent applications of human organoids include studies on infectious diseases, genetic disorders, cancers, and regenerative medicine,[29] as well as cell-based assays for pharmaceutical drug development and diagnostic purposes.[30] Most importantly, in line with the SARS-CoV-2 pandemic, organoids have been used to study mechanisms of entry and disease progression in kidney and liver.[31–33] In relation to this, we aimed to optimize a spatial protocol that is compatible with paraformaldehyde (PFA)-fixed organoid specimens. As a proof of principle, we used non-infected, PFA-fixed lung and kidney organoids embedded in optimal cutting temperature embedding medium (OCT). Fixation of organoids in PFA would allow researchers to be flexible in terms of storage, shipment, and the timing of embedding.

For the purpose of this study, between 6 and 8 organoids (<1 mm in diameter) were embedded together to fit within a single capture area (<42.25 mm^2), with each organoid covering between 50 and 150 spots (Figures S10B and S11B). 4 consecutive tissue sections were collected from the tissue block containing lung organoids and 3 tissue sections from the tissue block containing kidney organoids. From these datasets, 6 organoids were kept from each tissue type, excluding organoids covered by few spots. For the lung organoids, we detected an average number of unique genes per spot between 1,444 and 2,079 (Figure S10A), whereas for the kidney organoids we detected an average of 1,253 and 2,086 unique genes per spot (Figure S11A).

Unsupervised clustering of the lung organoids identified a large transcriptional variation across organoids (Figures S10C and S10D). In the major cluster 0, which was present in 4 out of 6 organoids, we found elevated expression of metabolic genes (*GAPDH*, *SCD*) together with the mesenchymal marker vimentin (*VIM*), indicating the presence of stem/progenitor cells. In addition, we observed an upregulation of *ENO2* in cluster 0, a known marker for pulmonary endocrine cells.[34] Cluster 1, which was only detected in organoid 4, displayed a distinct set of upregulated genes, including major histocompatibility complex (MHC) class I genes *HLA-B* and *HLA-C* as well as *LIN28A*, a marker for undifferentiated human embryonic stem cells.[35] Cluster 5 exemplified a region with a distinct expression profile that could only be detected in a single organoid (organoid 1), with an elevated expression of genes associated with mucosal tissues including surfactant protein B (*SFTPB*), anterior gradient protein 2 homolog (*AGR2*), and progastricsin (*PGC*).

In the kidney organoids, we observed an overall lower tissue heterogeneity, possibly reflecting a lower complexity of the model (Figures S11C and S11D). Cluster 2 co-localized with the organoid edge, expressing well-known markers for glomerular podocytes such as podocin (*NPHS2*) and podocalyxin-like protein 1 (*PODXL*).[36] In summary, these results indicate that spatially resolved transcriptomics data provide sufficient resolution to uncover heterogeneity in PFA organoids.

Spatial transcriptomics identifies molecular signatures associated with histopathological features in HGSC

To assess the suitability of our method on clinical FFPE samples, we generated spatially barcoded gene expression libraries from FFPE ovarian carcinosarcoma (high-grade serous carcinoma [HGSC] with a sarcomatous component). The samples had

CellPress
OPEN ACCESS

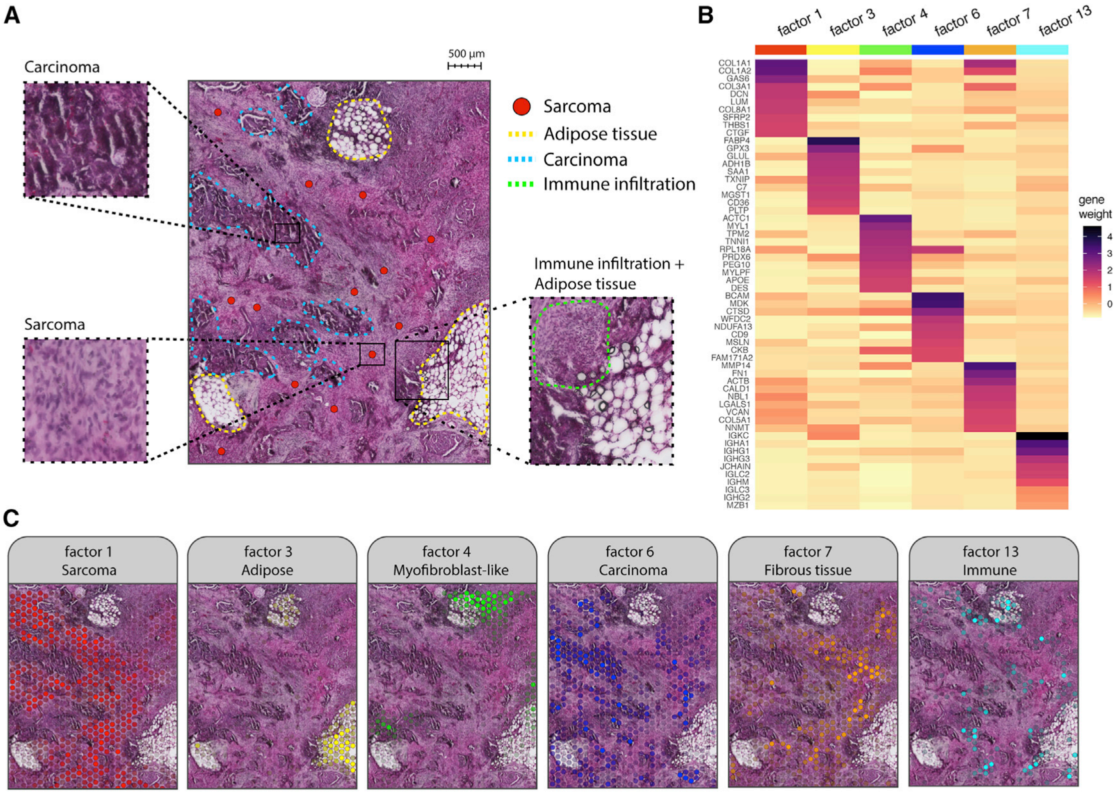

Figure 3. Exploratory analysis of FFPE ovarian carcinosarcoma tissue

(A) Histological image (H&E) of a representative area manually annotated based on morphological features. Blue dotted lines delimit areas with typical slit-like morphology associated with carcinoma. Yellow dotted lines mark fat lobules dispersed across the tissue. Sarcomatous areas (red dots) are mainly located in the spaces between the fat lobules and carcinoma, displaying various degrees of malignancy. Immune infiltration (green dotted area) is also visible in certain areas.
(B) Heatmap showing the top 10 most contributing genes for a selected panel of factors (values have been rescaled within each column).
(C) Spatial activity maps of a selected panel of factors (same as in B) showing the link between morphological features and expression-based patterns. The factor activity values have been rescaled to a range from 0 to 1. The spots with lower values are more transparent, whereas spots with higher values are less transparent.

been stored for 1 year and 8 months prior to the experiment. 2 adjacent sections were prepared, respectively, from two tissue blocks originating from one biopsy collected from the omentum. Bright-field images of the H&E-stained sections identified a heterogeneous mixture of mainly carcinoma and sarcoma cells interspersed by adipose lobules (Figure 3A) along with immune cell infiltration, vasculature, and the presence of a few scattered psammoma bodies. The carcinomatous element of the tumor was high-grade serous cancer, presenting a morphology of high cell density nests with numerous irregular slit-like spaces due to fusion of papillae (Figure 3A). The sarcomatous element was characterized by fibrous spindle cells with increased nuclear atypia (Figure 3A). On average, we detected 1,090 to 1,361 genes and 1,810 to 2,372 UMIs per spot (Figure S12A; Table S3). We found a strong correlation between adjacent sections ($R = 0.98$, $p = 2.2 \times 10^{-16}$; Figure S12B) as well as between sections collected from different sites ($R = 0.98$, $p = 2.2 \times 10^{-16}$; Figure S12C), indicating a high similarity in tissue composition from the two sites at bulk level.

To conduct robust cell type deconvolution of spatial transcriptomics data using cell type mapping methods, a representative scRNA-seq dataset should cover the majority of cell types present in the tissue section. For tissue types with high intra- and inter-patient heterogeneity, ideally, the scRNA-seq data should be paired with spatial transcriptomics data, i.e., profiled from the same tissue. Given that HGSC tumors represent highly heterogeneous tissues with high interpatient variability, we reasoned that publicly available scRNA-seq data collected from other patients did not fulfill the requirements to infer the correct cell type proportions. In addition, generating scRNA-seq data from FFPE tissue in-house remained a challenge, as formalin-fixed cells are difficult to isolate and process. Thus, in the absence of representative scRNA-seq data, we decided to use non-negative matrix factorization (NNMF)[37] as an exploratory method to deconvolve our HGSC data in an unbiased manner. In short, this approach models the gene expression as a conical linear combination of a predefined number of factors, where each factor is a latent variable that reflects variability in the data associated with sets of correlated genes. In contrast to cell type proportions inferred with cell type mapping methods, a factor can represent any source of biological variability such as homeostatic processes, cell types, and sets of co-localized cell types. Factors can be directly associated with the expression of specific marker genes by

exploring the gene loading (or gene weight) vectors, which determine how much each gene contributes to the factor activity.

In this analysis, we decomposed the spatial transcriptomics gene expression data from 4 HGSC tissue sections into a total of 15 factors, which were explored as spatial factor activity maps (Figure S13; Table S5). The selection process of 15 factors was by no means exhaustive, but they were selected to recapitulate the major morphological patterns observed in the H&E image. The superposition of the NNMF factor activities onto the histological images showed that factors were indeed spatially distributed and associations to observable histological features could be easily established by a pathologist with expertise on gynecological cancers (Figures 3A and 3C). The spatial activity maps were also consistent across consecutive sections, indicating low technical variability (Figures S12 and S13).

Factors 1, 7, and 12 were linked to the areas enriched for sarcoma cells by association of high factor activity, with histological features defined by pathological assessment of the H&E images (Figures 3A and 3C). Functional enrichment analysis of the top contributing genes for all 3 factors identified a strong enrichment for the epithelial-to-mesenchymal transition (EMT) term (Figure S14; Table S6),[38] partly defined by the upregulation of *COL1A1*, *COL1A2*, *COL3A1*, *VIM*, and *MMP2* (Table S5; Figure S13).

Similarly, for factors 2, 6, and 11, we found the association with regions enriched for carcinoma and *KRT7* to be a major contributor to the 3 factors, consistent with previous pathological assessment of KRT7+ status of the tumor (Table S5).

Interestingly, factor 4 spatially overlapped with a subgroup of mixed malignant cells, including both carcinoma and sarcoma in the vicinity of adipose tissue. Among the top driving genes, we found myofibroblast-associated genes (e.g., *ACTC1*, *MYL1*, *TPM2*) (Figures 3B, S13, and S14; Table S5). Cancer-associated myofibroblasts have been previously described in the literature in multiple cancer types[39–42] and have been hypothesized to induce other epithelial cells into a proliferative state, a mechanism that would normally direct stromal remodeling for regenerative and healing purposes.[39] Myofibroblastic-epithelial interactions have already been described in endometrial cancer[40] where myofibroblasts have been found expressing numerous growth factors and cyclins in the vicinity of tumor cells, thereby promoting tumor growth aside from proliferating themselves.

Factor 3 was spatially aligned to the adipose tissue regions and was also enriched for the expression of adipose-associated genes (Figures 3B, 3C, and S13; Table S5), whereas factor 5 mapped more specifically to the edges of fat lobules resembling mesothelium (Figure S13). Complement components emerged as the top contributing genes of this factor (*C3*, *C1S*, *C1R*, *CFB*). Based on functional enrichment analysis using the cancer hallmark gene set collection from MsigDB, factor 5 displayed an enrichment of the terms complement and coagulation (Figure S14). Factor 10 co-localized with arterioles and endothelial smooth muscle, driven by the expression of *VWF*, *SERPINF1*, and *A2M.* The factor also appeared in adipose tissue, although with a slightly attenuated signal.

The remaining factors (8, 9, and 13) were enriched for immune-related terms (Table S5), although with some differences between the factors. Factor 8 was driven by MHC class I and II genes, indicating the presence of antigen-presenting cells. In factor 13, we found immunoglobulins *(IGKC*, *IGHA1)* and *MZB1* among the top contributing genes (Figure S13; Table S5), suggesting that this factor represents plasma cells. Judging by the spatial distribution of this factor, the plasma cells are mainly found outside sarcoma areas, concentrated in smaller aggregates in the stroma and adipose tissue (Figure 3C).

Immune components of a COVID-19-infected lung

In the light of the recent COVID-19 pandemic, we also performed spatial genome-wide mRNA analysis on a stained FFPE lung biopsy from a 30-year-old male SARS-CoV-2 PCR-positive patient with several days of fever at the time of lung sampling. 2 adjacent tissue sections were collected (Figure 4A), covering a total of 1,504 spots with an average of 1,860 unique genes and 3,487 UMIs, respectively. Only 1 UMI count could be detected with a SARS-CoV-2 origin. The biopsied tissue consisted of: (1) a small piece of bronchus tissue (634 spots covered; Figures 4 and S15A) with bronchus-associated lymphoid tissue (BALT), a tertiary lymphoid structure (TLS), in the form of a densely packed follicle and (2) a small alveolar region with a bronchiole covering 208 spots (Figures 4 and S15B), showing extensive lymphocytic infiltration around the bronchus area (a more loosely packed form of BALT). BALT is not a constituent of healthy adult human lungs, as it appears only as a response to infection or inflammation, while it is found as a constituent in the lungs of children and adolescents with normal health profiles.[43,44] Given his age (30 years old) and his clinical history, the BALT observed in our COVID-19 patient is hypothesized to be an indication of post-infection.[43,45]

Given the limited detection of SARS-CoV-2 transcripts, we asked whether this observation could be explained by a low viral load in the tissue or the limited ability of our method to detect viral transcripts. To address this question, we ran an immunofluorescence (IF) assay targeting the SARS-CoV-1 spike protein, which has been shown to cross-react with the SARS-CoV-2 spike protein (Figure S16).[46] We segmented the nuclei from a DAPI staining and estimated a total number of approximately 4,000 cells in the tissue (see STAR Methods). Based on the immunostaining of the SARS-CoV-2 spike protein, we estimated only 89 positive cells (Figure S16; see STAR Methods), indicating a low level of viral infection. We considered this a plausible result given that the sample came from a patient who presented moderate symptoms of COVID-19 and that the viral dissemination in the lung typically follows multifocal heterogeneous patterns, where regions in the lung can appear nearly normal and viral particles are absent.[47]

To further explore the lung tissue infected with SARS-CoV-2 data in an unbiased manner, we used the NNMF method described earlier to deconvolve the spatial transcriptomics data into 15 factors. Each factor was annotated based on the top contributing genes associated with each factor and by superposition of the factor activity maps with the H&E images (Figure S17). In addition, we used a publicly available scRNA-seq dataset of the human lung[48] to explore the enrichment of differentially expressed genes associated with 25 different cell types (Figure S18). The enrichment of cell type marker genes was assessed

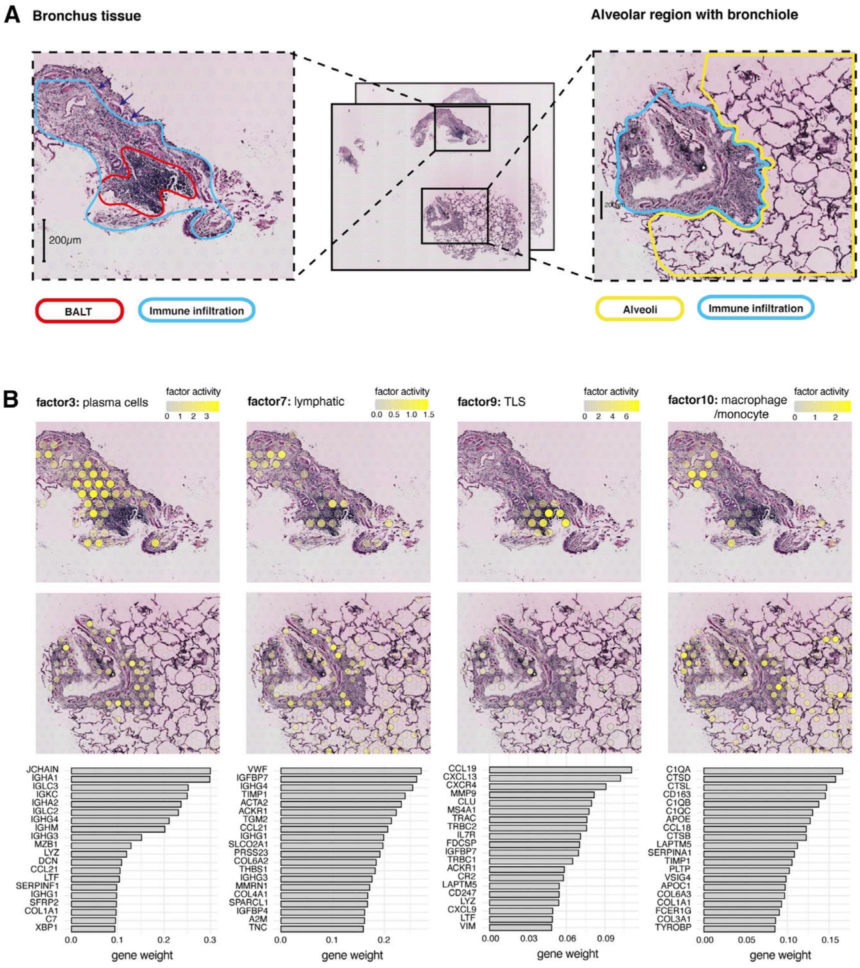

Figure 4. Analysis of COVID-19-infected lung tissue

(A) (Center) 2 tissue sections were collected from FFPE lung tissue infected with COVID-19 to obtain spatial transcriptomics data. 2 regions of the tissue sections (bronchus tissue and alveolar tissue with bronchiole) exemplify the association between morphological structures and molecular features. (Right and left) Zoomed-in view of selected regions annotated based on histological features of the tissue.

(B) Spatial factor activity maps visualized on H&E images for 4 selected factors related with BALT (top two rows). Top 20 driver genes per factor ranked by descending gene weights (bottom row).

for each spot in the spatial transcriptomics dataset by computing the area under the curve (AUC) score of cell-type-specific marker genes using a previously described method[49] (see STAR Methods). Thereafter, we computed the Pearson correlation between cell type enrichment and factor activity scores to find associations between cell type marker genes and factors (Figure S18). Although the scRNA-seq dataset represented cells from healthy tissue and not tissue infected with SARS-CoV-2, many of these associations were in line with the characteristics of the factors with regard to their top contributing genes and spatial location.

Factor 9 is colocalized with the TLS with multiple chemokines among the top driver genes, most notably *CCL19*, *CCL21*, and *CXCL13* (Figure 4C; Table S7), and also showed a high correlation with B- and T cell signatures (Figure S18). Follicular dendritic cells (FDCs) are main producers of *CXCL13* and *CCL21*, as they attract and organize immune cells within lymphoid tissues,[50] out of which *CXCL13* is required for the proper formation of densely packed B cell follicles.[51] As additional support for the presence of FDCs, we found *CR2* (CD21) among the top driver genes—one of the most commonly used FDC surface markers for IHC[52]—as well as the *FDCSP* marker gene (FDC secreted peptide precursor).[53] We also detected other lymphocytic markers including T cell receptor components *TRAC*, *TRBC1/2*, and the B cell marker *MS4A1* (CD20). Another interesting gene present in the BALT signature is lactoferrin (*LTF*), an innate immune system component known to suppress virus replication by affecting natural killer (NK) cells and macrophages, which has been found upregulated in association with SARS (SARS-CoV-1) in the past.[54]

The spots surrounding the edges of the follicle were assigned mainly to factor 3 (Figure 4B), driven predominantly by the expression of immunoglobulin (Ig) genes IgA (*JCHAIN*, *IGHA1*, and *IGHA2*), IgG (*IGHG3*, *IGHG1*), and IgM (*IGHM*) (Figure 4B; Table S7). These genes indicate an ongoing immune response by activated plasma cells, which was further supported by correlation with the plasma cell enrichment score (Figure S18). Consistent with this result, previous studies of BALT have found that plasma cells are often located around the edges of the follicles.[44,55,56]

Factor 7 showed a strong enrichment for the immune infiltrated regions, including the borders of the TLS (Figure 4B). It also expressed *CCL21,* a chemokine enriched in lymphoid tissue, which might indicate the presence of lymphatic vessels, as this chemokine is produced by lymphatic endothelial cells (LECs) lining the lymphatic vessels.[57] This result is consistent with previous reports that lymphatic vessels are typically found surrounding the follicle.[58]

Similarly, for factor 10, we found an association with immune infiltrated regions outside of the TLS (Figure 4B). The 3 building blocks of complement subcomponent C1q, synthesized and secreted by macrophages,[59,60] emerged among the top contributing genes of this factor (*C1QA*, *C1QB*, *C1QC*), along with other specific macrophage genes (*CD163*) and less specific ones (cathepsins: *CTSD*, *CTSL*, *CTSB*). In addition, we found a high correlation between factor 10 and the macrophage enrichment score (Figure S18) suggesting that the factor overall reflects the spatial distribution of macrophages in the tissue.

Investigation of archival samples

It has already been shown that RNA quality has a tendency to decline with the age of FFPE in bulk.[61,62] We expected to observe a similar effect when applying the spatial transcriptomics method to FFPE and asked ourselves if archival tissue blocks stored for longer periods of time (e.g., decades) could generate cDNA yields comparable to tissue blocks stored for a few years. We decided to test the RNA integrity using our TSO-based QC assay on 4 archival samples that had been stored for 37, 18, 10, and 5 years, respectively. We performed the TSO-based QC assay (Figure S19) prior to preparing sequencing libraries with our FFPE protocol (Table S2). Our results point toward a negative trend in the amount of polyadenylated RNA molecules as samples' storage age increases. We observed the highest cDNA yield in the 5-year-old FFPE block. However, the lowest cDNA was observed in the 18-year-old sample, which was slightly worse than the 37-year-old sample, exemplifying how age may not be the sole factor negatively affecting mRNA quality (Figure S20; Table S2). In conjunction with previously reported findings, our results suggest that the variability in RNA quality across FFPE samples is influenced by more factors than the time of storage.[62] In addition to storage time, we suggest that the sample handling and preservation conditions can have a severely negative effect on the RNA integrity and that both of these factors need to be investigated further to understand the limitations of the protocol with respect to sample types. We anticipate that some archival FFPE samples can have higher RNA integrity and encourage the use of our TSO-based QC assay to assess the potential of such samples.

DISCUSSION

In this study, we developed a new protocol for unbiased, spatially resolved transcriptomics of FFPE samples. For initial benchmarking, we applied this protocol on mouse tissues. Moreover, we showcase the applicability to recover genome-wide transcriptional profiles on a wide range of FFPE tissue types, including human lung and kidney organoids, and a small number of clinical samples.

Limitations of the study

Although we observed a lower RNA integrity in the oldest samples, we could also see a high variability in quality between samples of similar age. As tissue quality is imperative to generate high-quality data, we provide a TSO-based QC assay for tissue assessment prior to library preparation. However, an important limitation remains, as the assay only detects the captured mRNA, which has been successfully converted into double-stranded cDNA using the template switch reaction, but is unable to account for insert size. As a consequence, truncated mRNA molecules will still generate a signal by the assay but could be too short for subsequent size selection steps. However, to further evaluate the size distribution of captured mRNA after second strand synthesis, a PCR amplification of the cDNA followed by bioanalyzer can be used to complement the TSO-based QC assay.

Using the mouse brain as a model to evaluate quality aspects of data generated from FFPE tissues, we show that the recovery of mRNA is sparser compared with data generated from FF tissue sections. Although sparsity reduces the power of computational analyses, we show that the expression profiles in FFPE data are genome-wide and can be used to delineate sources of biological variability.

A third limitation relates to the exploration of archival samples with different storage ages. This analysis was limited to 4 FFPE blocks with long-term storage of more than 5 years from the same cancer type. A larger and more diverse set of samples would be required to confirm whether 5 years is the upper limit for this method to produce adequate data. However, stringency in pre-analytical steps (tissue handling before fixation) will be equally important in our ability to perform spatial high-quality RNA analysis with preserved morphology in archival samples.

Conclusions

Our spatial transcriptome analyses of FFPE data strongly correlate with data obtained from FF tissue at the genome-wide scale and cover enough complexity to delineate anatomical features in an unbiased manner. Cell type mapping by integration of spatially resolved transcriptomics and scRNA-seq presents the power to unravel key events in disease progression, immunological responses, and organ development. Strikingly, we found that cell type mapping produced similar results in our FFPE tissue section compared with FF tissue, indicating that our method is sensitive enough to recover cell-type-specific gene expression profiles. Nevertheless, we expect that the sparsity of the data presents a limitation when considering a more granular division of cell types.

The reproducibility aspect is of critical importance to generate high-quality data, especially when conducting studies on tissue collected from multiple individuals. We show that our protocol generates comparable quality metrics across consecutive tissue sections collected from the same tissue sample. Another quality-related aspect relates to the variability in the data observed across samples, individuals, and experiments, commonly referred to as batch effects. Such effects are often undesired as they obscure true biological variability. In our FFPE mouse brain tissue sections, we observed a high inter-sample variability in gene recovery but show that these data can be jointly analyzed using data integration methods developed for scRNA-seq. Following data integration, we applied unsupervised clustering to show that the data from 3 different experiments recapitulate a similar composition of clusters across the mouse brain tissue.

Motivated by the capability of our protocol to recover the spatially resolved transcriptomics data from FFPE mouse brain tissue and PFA-fixed lung and kidney organoids, we then moved on to apply the method to tissue types of a higher clinical value, starting with carcinosarcoma biospecimen. Our results suggest that the performance of the method is sufficient to conduct data-driven deconvolution of the molecular heterogeneity of cancers, identifying an intricate spatial composition of transcriptional programs tightly associated with known histopathological features in a carcinosarcoma tumor.

In light of the 2020 SARS-CoV-2 pandemic, we set out to test the performance of our method on FFPE tissue collected from the lungs of a patient with an ongoing infection. Following data-driven deconvolution of the lung tissue transcriptome, we

could localize stromal compartments of the lung tissue as well as lymphoid follicles in the form of a BALT, a type of TLS structure known to orchestrate lymphocyte maturation and activation in the lungs. Interestingly, pre-existing BALT in mice has been associated with reduced mortality in response to influenza, SARS-CoV-1, and mouse pneumovirus.[63] Thus, the low mortality rates observed in these age groups may come as a consequence of these individuals already hosting BALTs in their lungs, which could be playing a role, a hypothesis that prompts further investigation.

In conclusion, we have devised a simple approach which, to our knowledge, for the first time demonstrates unbiased genome-wide spatial gene expression profiling in FFPE biospecimens. For decades, FFPE has been the gold standard for the storage of cancer biopsies in clinical and research biobanks, and the prospect of spatially resolved expression profiling of these samples presents an exciting opportunity to study disease progression over both time and space.

STAR★METHODS

Detailed methods are provided in the online version of this paper and include the following:

- KEY RESOURCES TABLE
- RESOURCE AVAILABILITY
  - Lead contact
  - Materials availability
  - Data and code availability
- EXPERIMENTAL MODEL AND SUBJECT DETAILS
  - FFPE mouse brain sample
  - FFPE gynecological carcinosarcoma
  - FFPE archival clinical samples
  - PFA lung and kidney organoids
  - FFPE lung biopsy, SARS-CoV-2 infected
  - FFPE mouse brain sample replicate
- METHOD DETAILS
  - Genome-wide FFPE spatial expression profiling protocol
  - Immunofluorescence assay targeting coronavirus spike protein
  - TSO-based QC assay protocol
- QUANTIFICATION AND STATISTICAL ANALYSIS
  - Data pre-processing
  - Data processing
  - Selection of FFPE tissue section for comparison with fresh frozen data
  - Registration of H&E images to the Allen Mouse Brain Atlas anatomical reference with wholebrain
  - Analysis of a FFPE coronal tissue section of the mouse brain
  - Comparison of FFPE and FF mouse brain tissue sections
  - Visualization of marker gene expression by ISH
  - Cell type mapping with stereoscope
  - Analysis of multiple FFPE coronal sections from the mouse brain
  - Analysis of lung and kidney organoids
  - Filtering and normalization of carcinosarcoma (HGSC) datasets
  - Non-negative Matrix Factorization and pathway analysis of carcinosarcoma (HGSC) datasets
  - Analysis of lung tissue infected with Covid-19
  - Immunofluorescence assay targeting coronavirus spike protein image analysis
  - Archival FFPE tissue quality assessment

SUPPLEMENTAL INFORMATION

Supplemental information can be found online at https://doi.org/10.1016/j.xgen.2021.100065.

ACKNOWLEDGMENTS

This research has received funding from the European Union's Horizon 2020 research and innovation programme under grant agreement nos. 824110 EASI-Genomics, 874741 HUGODECA, and 874656 discovAIR; the Swedish Research Council; Swedish Cancer Society; the Swedish Foundation for Strategic Research; Knut and Alice Wallenberg Foundation (2018.0172); Erling-Persson Family Foundation (HDCA); Science for Life Laboratory; and the Swedish Childhood Cancer Fund. We would like to thank the National Genomics Infrastructure (NGI) Sweden for providing infrastructure support. We thank 10X Genomics, Margherita Zamboni, Sami Saarenpää, Ludvig Bergenstråhle, Mengxiao He, Zaneta Andrusivova, and Kim Thrane for help in the experimental work and for advice and helpful discussions.

AUTHOR CONTRIBUTIONS

E.G.V. led the experimental work and, together with R.M. and L.K., performed the experiments. L.L. analyzed the data, and L.L. and E.G.V. prepared the figures. A.A. performed the single-cell mapping analysis. A. Mollbrink contributed to experimental work in organoids, and G.K. contributed to experimental work in archival samples. K.S.A. prepared lung biopsy for the experiments. V.M. prepared organoids for the organoid experiments. N.S. provided a sample. N.M., H.Z., and J.M.P. contributed to develop organoids. W.M. provided lung biopsy. A. Mirazimi supervised organoids. J.C. designed the clinical experiments, provided clinical and archival samples, and helped interpretation of data. J.L. supervised the research. E.G.V., L.L., R.M., and J.L. wrote the manuscript.

DECLARATION OF INTERESTS

J.L., E.G.V., L.L., R.M., L.K., A.A., and A. Mollbrink are scientific consultants to 10x Genomics Inc, which holds IP rights to the ST technology.

Received: August 4, 2020
Revised: April 27, 2021
Accepted: August 30, 2021
Published: December 8, 2021

REFERENCES

1. Mathieson, W., and Thomas, G. (2019). Using FFPE Tissue in Genomic Analyses: Advantages, Disadvantages and the Role of Biospecimen Science. Curr. Pathobiol. Rep. *7*, 35–40.
2. Hoffman, E.A., Frey, B.L., Smith, L.M., and Auble, D.T. (2015). Formaldehyde crosslinking: a tool for the study of chromatin complexes. J. Biol. Chem. *290*, 26404–26411.
3. Asp, M., Bergenstråhle, J., and Lundeberg, J. (2020). Spatially Resolved Transcriptomes—Next Generation Tools for Tissue Exploration. Bioessays *42*, e1900221.
4. Ståhl, P.L., Salmén, F., Vickovic, S., Lundmark, A., Navarro, J.F., Magnusson, J., Giacomello, S., Asp, M., Westholm, J.O., Huss, M., et al. (2016).

Visualization and analysis of gene expression in tissue sections by spatial transcriptomics. Science *353*, 78–82.

5. Ji, A.L., Rubin, A.J., Thrane, K., Jiang, S., Reynolds, D.L., Meyers, R.M., Guo, M.G., George, B.M., Mollbrink, A., Bergenstråhle, J., et al. (2020). Multimodal Analysis of Composition and Spatial Architecture in Human Squamous Cell Carcinoma. Cell *182*, 497–514.e22.
6. Berglund, E., Maaskola, J., Schultz, N., Friedrich, S., Marklund, M., Bergenstråhle, J., Tarish, F., Tanoglidi, A., Vickovic, S., Larsson, L., et al. (2018). Spatial maps of prostate cancer transcriptomes reveal an unexplored landscape of heterogeneity. Nat. Commun. *9*, 2419.
7. Asp, M., Giacomello, S., Larsson, L., Wu, C., Fürth, D., Qian, X., Wärdell, E., Custodio, J., Reimegård, J., Salmén, F., et al. (2019). A Spatiotemporal Organ-Wide Gene Expression and Cell Atlas of the Developing Human Heart. Cell *179*, 1647–1660.e19.
8. Maniatis, S., Äijö, T., Vickovic, S., Braine, C., Kang, K., Mollbrink, A., Fagegaltier, D., Andrusivová, Ž., Saarenpää, S., Saiz-Castro, G., et al. (2019). Spatiotemporal dynamics of molecular pathology in amyotrophic lateral sclerosis. Science *364*, 89–93.
9. Civita, P., Franceschi, S., Aretini, P., Ortenzi, V., Menicagli, M., Lessi, F., Pasqualetti, F., Naccarato, A.G., and Mazzanti, C.M. (2019). Laser Capture Microdissection and RNA-Seq Analysis: High Sensitivity Approaches to Explain Histopathological Heterogeneity in Human Glioblastoma FFPE Archived Tissues. Front. Oncol. *9*, 482.
10. Zollinger, D.R., Lingle, S.E., Sorg, K., Beechem, J.M., and Merritt, C.R. (2020). GeoMx™ RNA Assay: High Multiplex, Digital, Spatial Analysis of RNA in FFPE Tissue. Methods Mol. Biol. *2148*, 331–345.
11. Cieslik, M., Chugh, R., Wu, Y.-M., Wu, M., Brennan, C., Lonigro, R., Su, F., Wang, R., Siddiqui, J., Mehra, R., et al. (2015). The use of exome capture RNA-seq for highly degraded RNA with application to clinical cancer sequencing. Genome Res. *25*, 1372–1381. http://genome.cshlp.org/content/25/9/1372.
12. Byron, S.A., Van Keuren-Jensen, K.R., Engelthaler, D.M., Carpten, J.D., and Craig, D.W. (2016). Translating RNA sequencing into clinical diagnostics: opportunities and challenges. Nat. Rev. Genet. *17*, 257–271.
13. 10x Genomics (2021). Visium Spatial Gene Expression Reagent Kits User Guide. https://support.10xgenomics.com/spatial-gene-expression/library-prep/doc/user-guide-visium-spatial-gene-expression-reagent-kits-user-guide.
14. Salmén, F., Ståhl, P.L., Mollbrink, A., Navarro, J.F., Vickovic, S., Frisén, J., and Lundeberg, J. (2018). Barcoded solid-phase RNA capture for Spatial Transcriptomics profiling in mammalian tissue sections. Nat. Protoc. *13*, 2501–2534.
15. Kennedy-Darling, J., and Smith, L.M. (2014). Measuring the formaldehyde Protein-DNA cross-link reversal rate. Anal. Chem. *86*, 5678–5681.
16. Evers, D.L., Fowler, C.B., Cunningham, B.R., Mason, J.T., and O'Leary, T.J. (2011). The effect of formaldehyde fixation on RNA: optimization of formaldehyde adduct removal. J. Mol. Diagn. *13*, 282–288. https://www.ncbi.nlm.nih.gov/pubmed/21497290.
17. Visium Spatial Tissue Optimization Reagents Kits User Guide Rev D CG000238 (2020). 10xGenomics.
18. Lein, E.S., Hawrylycz, M.J., Ao, N., Ayres, M., Bensinger, A., Bernard, A., Boe, A.F., Boguski, M.S., Brockway, K.S., Byrnes, E.J., et al. (2007). Genome-wide atlas of gene expression in the adult mouse brain. Nature *445*, 168–176.
19. Ortiz, C., Navarro, J.F., Jurek, A., Märtin, A., Lundeberg, J., and Meletis, K. (2020). Molecular atlas of the adult mouse brain. Sci. Adv. *6*, eabb3446.
20. Section, M.B. (2020). (Coronal), Spatial Gene Expression Dataset by Space Ranger 1.1.0, 10x. Genomics. https://www.10xgenomics.com/resources/datasets/mouse-brain-section-coronal-1-standard-1-1-0.
21. Fürth, D., Vaissière, T., Tzortzi, O., Xuan, Y., Märtin, A., Lazaridis, I., Spigolon, G., Fisone, G., Tomer, R., Deisseroth, K., et al. (2018). An interactive framework for whole-brain maps at cellular resolution. Nat. Neurosci. *21*, 139–149.
22. Zeisel, A., Hochgerner, H., Lönnerberg, P., Johnsson, A., Memic, F., van der Zwan, J., Häring, M., Braun, E., Borm, L.E., La Manno, G., et al. (2018). Molecular Architecture of the Mouse Nervous System. Cell *174*, 999–1014.e22.
23. Andersson, A., Bergenstråhle, J., Asp, M., Bergenstråhle, L., Jurek, A., Navarro, J.F., and Lundeberg, J. (2019). Spatial mapping of cell types by integration of transcriptomics data. bioRxiv. https://doi.org/10.1101/2019.12.13.874495.
24. Yao, Z., van Velthoven, C.T.J., Nguyen, T.N., Goldy, J., Sedeno-Cortes, A.E., Baftizadeh, F., Bertagnolli, D., Casper, T., Chiang, M., Crichton, K., et al. (2021). A taxonomy of transcriptomic cell types across the isocortex and hippocampal formation. Cell *184*, 3222–3241.e26.
25. Erö, C., Gewaltig, M.-O., Keller, D., and Markram, H. (2018). A Cell Atlas for the Mouse Brain. Front. Neuroinform. *12*, 84.
26. Tasic, B., Yao, Z., Graybuck, L.T., Smith, K.A., Nguyen, T.N., Bertagnolli, D., Goldy, J., Garren, E., Economo, M.N., Viswanathan, S., et al. (2018). Shared and distinct transcriptomic cell types across neocortical areas. Nature *563*, 72–78.
27. Korsunsky, I., Millard, N., Fan, J., Slowikowski, K., Zhang, F., Wei, K., Baglaenko, Y., Brenner, M., Loh, P.-R., and Raychaudhuri, S. (2019). Fast, sensitive and accurate integration of single-cell data with Harmony. Nat. Methods *16*, 1289–1296.
28. Dutta, D., Heo, I., and Clevers, H. (2017). Disease modeling in stem cell-derived 3D organoid systems. Trends Mol. Med. *23*, 393–410.
29. Kim, J., Koo, B.-K., and Knoblich, J.A. (2020). Human organoids: model systems for human biology and medicine. Nat. Rev. Mol. Cell Biol. *21*, 571–584.
30. Brandenberg, N., Hoehnel, S., Kuttler, F., Homicsko, K., Ceroni, C., Ringel, T., Gjorevski, N., Schwank, G., Coukos, G., Turcatti, G., and Lutolf, M.P. (2020). High-throughput automated organoid culture via stem-cell aggregation in microcavity arrays. Nat. Biomed. Eng. *4*, 863–874.
31. Monteil, V., Kwon, H., Prado, P., Hagelkrüys, A., Wimmer, R.A., Stahl, M., Leopoldi, A., Garreta, E., Hurtado Del Pozo, C., Prosper, F., et al. (2020). Inhibition of SARS-CoV-2 Infections in Engineered Human Tissues Using Clinical-Grade Soluble Human ACE2. Cell *181*, 905–913.e7.
32. Suzuki, T., Itoh, Y., Sakai, Y., Saito, A., Okuzaki, D., Motooka, D., Minami, S., Kobayashi, T., Yamamoto, T., Okamoto, T., et al. (2020). Generation of human bronchial organoids for SARS-CoV-2 research. bioRxiv. https://doi.org/10.1101/2020.05.25.115600.
33. Stebbing, J., Sánchez Nievas, G., Falcone, M., Youhanna, S., Richardson, P., Ottaviani, S., Shen, J.X., Sommerauer, C., Tiseo, G., Ghiadoni, L., et al. (2021). JAK inhibition reduces SARS-CoV-2 liver infectivity and modulates inflammatory responses to reduce morbidity and mortality. Sci. Adv. *7*, eabe4724.
34. Hor, P., Punj, V., Calvert, B.A., Castaldi, A., Miller, A.J., Carraro, G., Stripp, B.R., Brody, S.L., Spence, J.R., Ichida, J.K., et al. (2020). Efficient Generation and Transcriptomic Profiling of Human iPSC-Derived Pulmonary Neuroendocrine Cells. iScience *23*, 101083.
35. Richards, M., Tan, S.-P., Tan, J.-H., Chan, W.-K., and Bongso, A. (2004). The transcriptome profile of human embryonic stem cells as defined by SAGE. Stem Cells *22*, 51–64.
36. Harder, J.L., Menon, R., Otto, E.A., Zhou, J., Eddy, S., Wys, N.L., O'Connor, C., Luo, J., Nair, V., Cebrian, C., et al.; European Renal cDNA Bank (ERCB); Nephrotic Syndrome Study Network (NEPTUNE) (2019). Organoid single cell profiling identifies a transcriptional signature of glomerular disease. JCI Insight *4*, 122697.
37. Wu, Y., Tamayo, P., and Zhang, K. (2018). Visualizing and Interpreting Single-Cell Gene Expression Datasets with Similarity Weighted Nonnegative Embedding. Cell Syst. *7*, 656–666.e4.
38. Loret, N., Denys, H., Tummers, P., and Berx, G. (2019). The Role of Epithelial-to-Mesenchymal Plasticity in Ovarian Cancer Progression and Therapy Resistance. Cancers (Basel) *11*, 838. https://pubmed.ncbi.nlm.nih.gov/31213009.

39. Otranto, M., Sarrazy, V., Bonté, F., Hinz, B., Gabbiani, G., and Desmoulière, A. (2012). The role of the myofibroblast in tumor stroma remodeling. Cell Adhes. Migr. *6*, 203–219.

40. Orimo, A., Tomioka, Y., Shimizu, Y., Sato, M., Oigawa, S., Kamata, K., Nogi, Y., Inoue, S., Takahashi, M., Hata, T., and Muramatsu, M. (2001). Cancer-associated myofibroblasts possess various factors to promote endometrial tumor progression. Clin. Cancer Res. *7*, 3097–3105.

41. Karvonen, H.M., Lehtonen, S.T., Sormunen, R.T., Lappi-Blanco, E., Sköld, C.M., and Kaarteenaho, R.L. (2014). Lung cancer-associated myofibroblasts reveal distinctive ultrastructure and function. J. Thorac. Oncol. *9*, 664–674.

42. Ding, L., Zhang, Z., Shang, D., Cheng, J., Yuan, H., Wu, Y., Song, X., and Jiang, H. (2014). α-Smooth muscle actin-positive myofibroblasts, in association with epithelial-mesenchymal transition and lymphogenesis, is a critical prognostic parameter in patients with oral tongue squamous cell carcinoma. J. Oral Pathol. Med. *43*, 335–343.

43. Tschernig, T., and Pabst, R. (2000). Bronchus-associated lymphoid tissue (BALT) is not present in the normal adult lung but in different diseases. Pathobiology *68*, 1–8.

44. Randall, T.D. (2010). Bronchus-Associated Lymphoid Tissue (BALT): Structure and Function. In Advances in Immunology, S. Fagarasan and A. Cerutti, eds. (Academic Press), pp. 187–241.

45. Delventhal, S., Hensel, A., Petzoldt, K., and Pabst, R. (1992). Effects of microbial stimulation on the number, size and activity of bronchus-associated lymphoid tissue (BALT) structures in the pig. Int. J. Exp. Pathol. *73*, 351–357.

46. Zheng, Z., Monteil, V.M., Maurer-Stroh, S., Yew, C.W., Leong, C., Mohd-Ismail, N.K., Cheyyatraivendran Arularasu, S., Chow, V.T.K., Lin, R.T.P., Mirazimi, A., et al. (2020). Monoclonal antibodies for the S2 subunit of spike of SARS-CoV-1 cross-react with the newly-emerged SARS-CoV-2. Euro Surveill. *25*, 2000291.

47. Grant, R.A., Morales-Nebreda, L., Markov, N.S., Swaminathan, S., Querrey, M., Guzman, E.R., Abbott, D.A., Donnelly, H.K., Donayre, A., Goldberg, I.A., et al.; NU SCRIPT Study Investigators (2021). Circuits between infected macrophages and T cells in SARS-CoV-2 pneumonia. Nature *590*, 635–641.

48. Travaglini, K.J., Nabhan, A.N., Penland, L., Sinha, R., Gillich, A., Sit, R.V., Chang, S., Conley, S.D., Mori, Y., Seita, J., et al. (2020). A molecular cell atlas of the human lung from single-cell RNA sequencing. Nature *587*, 619–625.

49. Aibar, S., González-Blas, C.B., Moerman, T., Huynh-Thu, V.A., Imrichova, H., Hulselmans, G., Rambow, F., Marine, J.-C., Geurts, P., Aerts, J., et al. (2017). SCENIC: single-cell regulatory network inference and clustering. Nat. Methods *14*, 1083–1086.

50. Ansel, K.M., Ngo, V.N., Hyman, P.L., Luther, S.A., Förster, R., Sedgwick, J.D., Browning, J.L., Lipp, M., and Cyster, J.G. (2000). A chemokine-driven positive feedback loop organizes lymphoid follicles. Nature *406*, 309–314.

51. Rangel-Moreno, J., Moyron-Quiroz, J.E., Hartson, L., Kusser, K., and Randall, T.D. (2007). Pulmonary expression of CXC chemokine ligand 13, CC chemokine ligand 19, and CC chemokine ligand 21 is essential for local immunity to influenza. Proc. Natl. Acad. Sci. USA *104*, 10577–10582.

52. Kurshumliu, F., Sadiku-Zehri, F., Qerimi, A., Vela, Z., Jashari, F., Bytyci, S., Rashiti, V., and Sadiku, S. (2019). Divergent immunohistochemical expression of CD21 and CD23 by follicular dendritic cells with increasing grade of follicular lymphoma. World J. Surg. Oncol. *17*, 115.

53. Marshall, A.J., Du, Q., Draves, K.E., Shikishima, Y., HayGlass, K.T., and Clark, E.A. (2002). FDC-SP, a novel secreted protein expressed by follicular dendritic cells. J. Immunol. *169*, 2381–2389.

54. Reghunathan, R., Jayapal, M., Hsu, L.-Y., Chng, H.-H., Tai, D., Leung, B.P., and Melendez, A.J. (2005). Expression profile of immune response genes in patients with Severe Acute Respiratory Syndrome. BMC Immunol. *6*, 2.

55. GeurtsvanKessel, C.H., Willart, M.A., Bergen, I.M., van Rijt, L.S., Muskens, F., Elewaut, D., Osterhaus, A.D., Hendriks, R., Rimmelzwaan, G.F., and Lambrecht, B.N. (2009). Dendritic cells are crucial for maintenance of tertiary lymphoid structures in the lung of influenza virus-infected mice. J. Exp. Med. *206*, 2339–2349.

56. Rangel-Moreno, J., Hartson, L., Navarro, C., Gaxiola, M., Selman, M., and Randall, T.D. (2006). Inducible bronchus-associated lymphoid tissue (iBALT) in patients with pulmonary complications of rheumatoid arthritis. J. Clin. Invest. *116*, 3183–3194.

57. Johnson, L.A., and Jackson, D.G. (2010). Inflammation-induced secretion of CCL21 in lymphatic endothelium is a key regulator of integrin-mediated dendritic cell transmigration. Int. Immunol. *22*, 839–849.

58. Carragher, D.M., Rangel-Moreno, J., and Randall, T.D. (2008). Ectopic lymphoid tissues and local immunity. Semin. Immunol. *20*, 26–42.

59. Sellar, G.C., Blake, D.J., and Reid, K.B. (1991). Characterization and organization of the genes encoding the A-, B-and C-chains of human complement subcomponent C1q. The complete derived amino acid sequence of human C1q. Biochem. J. *274*, 481–490.

60. Zhou, A.Q., Herriott, M.J., and Leu, R.W. (1991). Kinetics of the biosynthesis of complement subcomponent C1q by murine macrophages: LPS, immune complexes, and zymosan alone and in combination with interferon-γ. J. Leukoc. Biol. *50*, 453–463.

61. Wehmas, L.C., Wood, C.E., Chorley, B.N., Yauk, C.L., Nelson, G.M., and Hester, S.D. (2019). Enhanced Quality Metrics for Assessing RNA Derived From Archival Formalin-Fixed Paraffin-Embedded Tissue Samples. Toxicol. Sci. *170*, 357–373.

62. von Ahlfen, S., Missel, A., Bendrat, K., and Schlumpberger, M. (2007). Determinants of RNA quality from FFPE samples. PLoS ONE *2*, e1261.

63. Wiley, J.A., Richert, L.E., Swain, S.D., Harmsen, A., Barnard, D.L., Randall, T.D., Jutila, M., Douglas, T., Broomell, C., Young, M., and Harmsen, A. (2009). Inducible bronchus-associated lymphoid tissue elicited by a protein cage nanoparticle enhances protection in mice against diverse respiratory viruses. PLoS ONE *4*, e7142.

64. Tissue Preparation Guide for Visium Demonstrated Protocol CG000240 RevC (2020). 10x Genomics.

65. Methanol Fixation, H. &E Staining & Imaging for Visium Spatial Protocols, Document Number CG000160 Rev A, 10x Genomics, (2020, June 23)

66. Martin, M. (2011). Cutadapt removes adapter sequences from high-throughput sequencing reads. EMBnet.journal *17*, 3.

67. /goldenPath/wuhCor1. https://hgdownload.soe.ucsc.edu/goldenPath/wuhCor1/.

68. Bergenstråhle, J., Larsson, L., and Lundeberg, J. (2020). Seamless integration of image and molecular analysis for spatial transcriptomics workflows. BMC Genomics *21*, 482.

69. Webber, W., Moffat, A., and Zobel, J. (2010). A similarity measure for indefinite rankings. ACM Trans. Inf. Syst. Secur. *28*, 1–38.

70. Andersson, A., Bergenstråhle, J., Asp, M., Bergenstråhle, L., Jurek, A., Fernández Navarro, J., and Lundeberg, J. (2020). Single-cell and spatial transcriptomics enables probabilistic inference of cell type topography. Commun. Biol. *3*, 565.

71. Liberzon, A., Subramanian, A., Pinchback, R., Thorvaldsdóttir, H., Tamayo, P., and Mesirov, J.P. (2011). Molecular signatures database (MSigDB) 3.0. Bioinformatics *27*, 1739–1740.

72. Pau, G., Fuchs, F., Sklyar, O., Boutros, M., and Huber, W. (2010). EBImage–an R package for image processing with applications to cellular phenotypes. Bioinformatics *26*, 979–981.

STAR★METHODS

KEY RESOURCES TABLE

REAGENT or RESOURCE	SOURCE	IDENTIFIER
**Antibodies**		
SARS-CoV-1 monoclonal antibody	Public Health Agency of Sweden, Ali Mirazimi	https://ki.se/en/labmed/research-group-ali-mirazimi
goat anti-Mouse IgG (H+L) Alexa Fluor 555 conjugate	Abcam	Cat#A32727, RRID: AB_2633276
**Biological samples**		
FFPE Mouse Brain, Male, 25 g, 8-12 weeks of age	Adlego Biomedical	C57BL6J
FFPE Gynecological Carcinosarcoma	Department of Oncology-Pathology, Karolinska Institute, Sweden. Joseph Carlson	https://ki.se/en/onkpat/joseph-carlsons-group
FFPE Archival Clinical samples (soft tissue sarcomas)	Department of Oncology-Pathology, Karolinska Institute, Sweden. Joseph Carlson	https://ki.se/en/onkpat/joseph-carlsons-group
FFPE Colon xenograft	Department of Oncology-Pathology, Karolinska Institute, Sweden. Niklas Schultz	https://staff.ki.se/people/nischu
PFA Lung and Kidney Organoids	Public Health Agency of Sweden, Ali Mirazimi	https://ki.se/en/labmed/research-group-ali-mirazimi
FFPE Lung biopsy, SARS-CoV-2 infected	Department of Haemostaseology and Haemophilia Center, Institute of Transfusion Medicine, Medical Clinic 2, University Hospital Frankfurt, Germany. Wolfgang Mieschbach	https://www.kgu.de/einrichtungen/kliniken/zentrum-der-inneren-medizin/medizinische-klinik-2-haematologie-onkologie-haemostaseologie-rheumatologie-infektiologiehiv/
**Chemicals, peptides, and recombinant proteins**		
Xylene	VWR	Cat#28975.291
EtOH 99%	VWR	Cat#84835.290
EtOH 96%	VWR	Cat#20823.290
Formaldehyde 37%	Sigma-Aldrich	Cat#F8775-25ML
Sucrose	Sigma-Aldrich	Cat#84097-1KG
HBSS buffer	Life Technologies	Cat#14025-050
TissueTek O.C.T. Compound	VWR	Cat#25608-930
Isopentane	Millipore Sigma	Cat#270342
BSA	Bionordika	Cat#B9000S
collagenase I	Life Technologies	Cat#17018-029
SSC buffer	Sigma-Aldrich	Cat#S6639-1L
TE buffer pH 8.0	ThermoFisher	Cat#AM9849
Pepsin	Sigma-Aldrich	Cat#P7000-25G
HCl	Sigma-Aldrich	Cat#318965-1000ML
Tris 1M, pH 7.0, RNase-free	ThermoFisher	Cat#AM9850G
KAPA SYBR FAST qPCR Master Mix (2X)	Millipore Sigma	Cat#KK4600
SPRIselect beads	Beckman Coulter	Cat#B23318
Buffer EB	QIAGEN	Cat#19086
**Critical commercial assays**		
Visium Spatial Gene Expression Slide and Reagent Kit 16 rxns	10x Genomics	PN-1000184

(*Continued on next page*)

Continued		
REAGENT or RESOURCE	SOURCE	IDENTIFIER
Dual Index Kit TT Set A, 96 rxns	10x Genomics	PN-1000215
Visium Accessory Kit	10x Genomics	PN-1000194
BioAnalyzer High Sensitivity chip	Agilent	Cat#5067-4626
Qubit dsDNA HS Assay Kit, 100 assays	ThermoFisher	Cat#Q32851
Visium Spatial Tissue Optimization Slide & Reagents Kit	10x Genomics	PN-1000193
Deposited data		
Raw sequence data	GEO	GSE185715
Count matrices, low res H&E images, coordinate files and immunofluorescence images	Mendeley Data	https://doi.org/10.17632/xjtv62ncwr.1
High resolution H&E images	Mendeley Data	https://doi.org/10.17632/xjtv62ncwr.1
Shiny application	GitHub	https://github.com/ludvigla/FFPE_mouse_brain_explorer
Oligonucleotides		
Fluorescent cy3-TSOprobe: Purification HPLC /5Cy3/AA GCA GTG GTA TCA ACG CAG AGT ACA TGG G	Integrated DNA Technologies	N/A
Custom primer for Read 2: Purification HPLC AAG CAG TGG TAT CAA CGC AGA GTA CAT GGG	Integrated DNA Technologies	N/A
Software and algorithms		
VSlide	MetaSystems	https://metasystems-international.com/en/products/solutions/tissue-imaging/
Spaceranger v1.0.0	10x Genomics	https://support.10xgenomics.com/spatial-gene-expression/software/overview/welcome
Loupe Browser	10x Genomics	https://www.10xgenomics.com/products/loupe-browser
wholebrain v0.1.1	Fürth et al., 2017	http://www.wholebrainsoftware.org/
STutility v0.1.0	Bergenstråhle et al., 2019	https://github.com/jbergenstrahle/STUtility
Seurat v3.1.5	Stuart et al., 2019	https://github.com/satijalab/seurat/releases/tag/v3.1.5
sctransform v0.2.1	Hafemeister et al., 2019	https://github.com/ChristophH/sctransform
ggplot2 v3.3.0	Hadley Wickham, 2016	https://ggplot2.tidyverse.org/
ggpubr v0.3.0	N/A	https://github.com/kassambara/ggpubr
gespeR v1.22.0	Schmich F., 2020	https://bioconductor.org/packages/release/bioc/html/gespeR.html
Stereoscope v3.0	Andersson et al., 2019	https://github.com/almaan/stereoscope
harmony v1.0	Korsunsky et al., 2019	https://github.com/immunogenomics/harmony
NNLM v0.4.4	Wu et al., 2018	https://github.com/linxihui/NNLM
gprofiler2 v0.1.9	Kolberg et al., 2020	https://cran.r-project.org/web/packages/gprofiler2/index.html
AUCell v1.10.0	Aibar et al., 2017	https://bioconductor.org/packages/release/bioc/html/AUCell.html
EBImage v4.30.0	Pau et al., 2010	https://bioconductor.org/packages/release/bioc/html/EBImage.html
ImageJ v2.1.0/1.53c	ImageJ	https://imagej.net
Adobe Photoshop	Adobe	https://www.adobe.com/products/photoshop.html

RESOURCE AVAILABILITY

Lead contact

Further information and requests should be directed to the Lead Contact, Joakim Lundeberg (joakim.lundeberg@scilifelab.se)

Materials availability

This study did not generate new unique reagents.

Data and code availability

RNA-seq data, output files from spaceranger and HE images have been deposited at GEO: GSE185715 and are publicly available. Access to raw sequencing data from the HGSC and archival samples is provided under an MTA from Joseph Carlson. Access to raw sequencing data from lung is provided under an MTA from Ali Mirazimi. Processed data have been deposited at Mendeley: https://doi.org/10.17632/xjtv62ncwr.1 and are publicly available. The Shiny application, a public data browser for viewing the data generated in this study, is available at Github: https://github.com/ludvigla/FFPE_mouse_brain_explorer, and listed in the Key resources table.

All original code has been deposited at Mendeley: https://doi.org/10.17632/xjtv62ncwr.1 and is publicly available.

Any additional information required to re-analyze the data reported in this paper is available from the lead contact upon request.

EXPERIMENTAL MODEL AND SUBJECT DETAILS

FFPE mouse brain sample

Healthy male, 25 g, 8-12 weeks of age. Sample number C57BL6J (Adlego Biomedical). Extracted under ethical permit number 4570-2019. RIN 2.9, DV200 65%.

FFPE gynecological carcinosarcoma

Female. High grade serous ovarian carcinosarcoma metastasis to the omentum. Untreated. Two regions: 1919-1 RIN 2.5, DV200 69% and 1919-2 RIN 2.30, DV200 64%.

FFPE archival clinical samples

De-identified control tissues consisting of soft tissue sarcomas collected in 1983, 2002, 2010, 2015.

PFA lung and kidney organoids

Human lung and kidney organoids were cultured as previously described[31] (Monteil., 2020).

FFPE lung biopsy, SARS-CoV-2 infected

Male, 30 years old, SARS-Cov-2-PCR- positive. Patient had suffered from fever for several days at the time of lung sampling.

FFPE mouse brain sample replicate

Healthy male, 25 g, 8-12 weeks of age. Sample number C57BL6J (Adlego Biomedical). Extracted under ethical permit number 4570-2019.

METHOD DETAILS

Genome-wide FFPE spatial expression profiling protocol

Preparation of FFPE samples - Part 1

Microtome sections (thickness specified on Table S2) were placed on Visium Spatial Gene Expression slides (10x Genomics) after floating on a water bath at 43°C. After sectioning, the slides were dried vertically at 40°C in a hybridization oven (HB-1000 Hybridizer, LabRepCo) for 1 hour and 45 min. The slides were then placed inside a slide mailer, sealed with parafilm, and left overnight in a refrigerator at 4°C. The slides were deparaffinized by immersion in the following reagents: Xylene (VWR) 15 min twice, EtOH 99% (VWR) 2 min twice, EtOH 96% (VWR) 2 min twice, EtOH 70% (diluted from EtOH96%) 2 min twice, H20 5 min.

Preparation of PFA samples - Part 1

Organoids were fixed for 24 h in 4% PFA, followed by immersion in sucrose gradients of 15%, then exchanged with 30% sucrose. Embedding and sectioning were performed according to the Tissue Preparation Guide for Visium Demonstrated Protocol CG000240 RevC, 10x Genomics[64] from Step 1.3 onward: an isopentane bath in a liquid nitrogen bath was prepared. The organoids were submerged into the isopentane until fully frozen, then they were transferred into a cryomold previously filled with pre-chilled O.C.T. for embedding avoiding bubbles. The cryomold containing the tissue and O.C.T. was immediately placed on dry ice until frozen. Next, the O.C.T.-embedded tissue block was mounted on a specimen stage inside a cryostat cooled at −20°C for blade and −15°C for specimen head. The stage was installed and sectioning was done until obtaining 10 μm thick sections that were flattened out and

placed within a capture area on a pre-equilibrated Visium Spatial Gene Expression array. Thickness specified on Table S2. The sections adhered to the capture area by gently placing a finger on the backside of the capture area for a few seconds.

H&E staining - Part 2

Staining was performed according to the Methanol Fixation, H&E staining & Imaging for Visium Demonstrated Protocol CG000160 RevA, 10x Genomics,[65] step 1.3. All steps took place at room temperature. 500 μl isopropanol were pipetted uniformly covering the capture areas for 1 min. Excess isopropanol was drained by holding the slide at an angle with the bottom edge on a folded paper wipe. The slides air-dried quickly and 500 μl hematoxylin were added to cover the capture areas for 4 min. The slide was dipped inside a falcon tube containing 50 mL of nuclease-free water to discard the majority of the hematoxylin and dipped 15x in an 800 mL nuclease-free water beaker immediately after. Next, excess liquid was wiped and on a flat surface 500 μl Dako bluing buffer (Agilent) were added covering the capture areas and incubated for 30 s. The slide was then dipped 15x in a second 800 mL nuclease-free water beaker, followed by addition of Eosin counterstain mix (100 μl Eosin Y Solution + 900 μl Tris-Acetic Acid Buffer (0.45 M, pH 6.0)) to uniformly cover the sections and incubated for 1 min. The Eosin mix was discarded by holding the slide at an angle with the bottom edge on a folded paper wipe and the slide was dipped 15x in a third beaker containing 800 mL nuclease-free water. After this step, slides were mounted for microscopic imaging with 200 μl glycerol 85% and a cover glass applied on the slide.

H&E imaging - Part 3

Slides were scanned under a high-resolution microscope Metafer Slide Scanning Platform (Metasystems) to obtain tissue tile images and software VSlide (Metasystems) to stitch the high-resolution images together. After imaging, the glycerol and cover glass were carefully removed by holding the slides in an 800 mL water beaker and letting the glycerol diffuse until the cover glass detached and density changes were no longer visible in the water. The slides were then dried at 37°C.

Analyte retrieval and permeabilization - Part 4

Slides were mounted in an ArrayIt metallic hybridization cassette (#AHC1X16 ArrayIt) or plastic slide cassettes from 10x Visium kit. For FFPE samples, a collagenase mix (986 μl HBSS buffer (Life Technologies), 10 μl BSA (Bionordika), 4 μl collagenase I (50 U/μl, Life Technologies)) was equilibrated to 37°C and then 75 μl were added to each of the wells in the cassette. The slides were sealed and incubated for 20 min at 37°C in a Thermoblock (ThermoMixer with Thermoblock, Eppendorf) with a heated lid (ThermoTop, Eppendorf). Once the incubation was complete, the collagenase mix was pipetted off and the slides were washed by with 100 μl of 0.1 x SSC buffer (Sigma-Aldrich, diluted in RNase DNase free MQ) in each well. Collagenase pre-permeabilization was skipped in the case of PFA samples.

Subsequently, 100 μl TE buffer pH 8.0 (ThermoFisher) was added, and the slides were sealed and incubated for 1 hour (20 min for PFA samples) at 70°C in a Thermoblock with heated lid. After the incubation, the slides were taken out of the Thermoblock and left to equilibrate at room temperature for 5 min. Meanwhile, 0.1% pepsin solution (P7000-25G Sigma-Aldrich) dissolved in 0.1M HCl (Sigma-Aldrich) was equilibrated to 37°C. After the incubation and one wash per well with 100 μl 0.1 x SSC buffer, permeabilization of the tissue was carried out by adding 75 μl pepsin solution, sealing the slides and incubating with heated lid at 37°C for 30 min (10 min for PFA samples). After this step, 0.1X SSC buffer was added to wash the pepsin solution. The following steps were performed according to the Visium Spatial Gene Expression User Guide CG000239 Rev C, 10x Genomics.[13]

Reverse transcription – Part 5

Reverse Transcription was performed as described in Step 1.2 of the User Guide: the slides were still masked in ArrayIt metallic hybridization cassettes or Visium Slide cassettes. First, 75 μl of RT Master Mix (37.8 μl Nuclease-free water + 18.8 μl RT reagent + 5.22 μl TSO + 1.5 μl Reducing Agent B + 11.7 μl RT Enzyme D) was added to each well. The reverse transcription step was conducted either for 45 min at 53°C or with a pre-step at lower temperature (60 min at 42°C), thus increasing the overall incubation times (see Table S2). All step names and numbers cited in the document correspond to the Visium Spatial Gene Expression User Guide CG000239 Rev C unless specified otherwise.

Second strand synthesis and denaturation - Part 6

Second strand synthesis and denaturation were carried out as described in the standard Visium Spatial Gene Expression User Guide Step 2 with the difference that the slides were masked in ArrayIt metallic incubation chambers instead of Visium Slide cassettes: the RT Master Mix was removed from the wells, and 75 μl 0.08 M KOH was added on each well to dehybridize template mRNA and incubated for 5 min at room temperature. Next KOH was discarded and the wells were washed once with 100 μl buffer EB per well. Then 75 μl Second Strand Mix (69.5 μl SS Reagent + 4.0 μl SS Primer + 1.5 μl SS Enzyme) were added to each well. Next, slides were incubated at 65°C for 15 min and cooled down to 4°C. After that, wells were washed with 100 μl EB buffer, which was removed and 35 μl 0.08 M KOH were added into each well, this time to denature the newly-synthesized second strands. After a 10 min incubation, the well contents were transferred to separate PCR tubes in an 8-tube strip previously containing 5 μl Tris (1M, pH 7.0) per well.

cDNA amplification, cleanup and quantification - Part 7

The denatured second strands were evaluated by qPCR as in Visium Spatial Gene Expression User Guide Step 3.1: 9 μl of qPCR mix (20.4 μl Nuclease-free water + 27.5 μl KAPA SYBR FAST qPCR Master Mix + 1.7 μl cDNA Primers) were added to each well in a qPCR plate, including a well for negative control. Then, 1 μl of sample was transferred to each well of the qPCR plate and 1 μl nuclease-free water to the negative control well. The following protocol was run in a qPCR system: Step 1; 98°C, 3 min. Step 2; 98°C, 5 s. Step 3; 63°C, 30 s and read fluorescent signal. Step 4; Go to step 2 for a total of 25 cycles. The number of PCR cycles for cDNA amplification was determined by the Cq value at a fourth of the peak fluorescence value per sample in the qPCR.

Samples were then amplified by PCR by addition of 65 μl cDNA Amplification Mix (220 μl Amp Mix + 66 μl cDNA primers) to the remaining sample volumes and run with settings specified in Visium Spatial Gene Expression User Guide Step 3.2 (Step 1; 98°C, 3 min. Step 2; 98°C, 15 s. Step 3; 63°C, 20 s. Step 4; 72°C, 1 min. Step 5; Go to Step 2 for the total number of cycles. Step 6; 72°C, 1 min. Step 7; 4°C hold. Heated lid temperature: 105°C, reaction volume: 100 μl) The number of PCR cycles performed is specified in Table S2.

Sample cleanup was performed using 0.8X SPRIselect beads, meaning that 80 μl of SPRIselect beads were added to 100 μl sample and incubated at 5 min at room temperature. The 8-tube strip was then placed on the high magnet position until the solution cleared. The the supernatant was discarded and the beads were carefully washed twice with 200 μl 80% EtOH. Then the beads were resuspended in 15 μl Buffer EB for eluting was instead of the 40.5 μl specified in Step 3.3.j of the protocol. They were let incubate for 2 min at room temperature and then placed in the low magnet position until the solution cleared, moment in which the supernatant was transferred to a new 8-tube strip. The cDNA yield was checked by using 1μl sample for quality control on a BioAnalyzer High Sensitivity chip (Agilent) as in Step 3.4.

Library preparation - Part 8

Sequencing libraries were generated according to the manufacturer's protocol with some modifications due to the shorter size of the mRNA molecules that is expected after formaldehyde treatment with respect to its fragmentation effect. For instance, double-sided selection was kept only after indexing PCR, 0.6x ratios were changed to 0.8x ratios and one additional bead selection was incorporated in the end. Fragmentation time was reduced as well to avoid fragment size to become too short.

Fragmentation, end repair and A-tailing - Part 8.1

A total of 10 μl of each sample were fragmented in a thermal cycler as described in step 4.1 with the alteration that fragmentation run time was reduced to 1 min instead of 5 min. 10 μl of purified cDNA samples were diluted with 25 μl Buffer EB per well and then 15 μl Fragmentation Mix (22 μl Fragmentation Buffer + 44 μl Fragmentation enzyme) were added to each well. The program in the thermal cycler was the following: Step 1; Pre-cool to 4°C hold. Step 2; Fragmentation 32°C 1 min. Step 3; End-repair and A-tailing 65°C 30 min. Step 4; 4°C, hold. Heated lid temperature: 65°C, reaction volume: 50 μl).

Samples were cleaned up post-fragmentation using 0.8x SPRIselect beads. 40 μl SPRIselect beads were added to 50 μl sample and incubated at room temperature for 5 min. The tubes were then placed on the high magnet position until the solution cleared. Supernatant was discarded and the beads were washed with 125 μl 80% EtOH for 30 s two times. After removing the 80% EtOH, beads were allowed to dry on the magnet for less than 2 min (avoiding over-drying) followed by addition of 50.5 μl Buffer EB to each sample. The samples were removed from the magnet, vortexed, and centrifuged briefly to mix the beads with Buffer EB and were then incubated for 2 min at room temperature. After incubation the samples were placed on the low magnet position until the solution cleared. 50 μl of each sample was transferred to a new tube strip.

Adaptor ligation SPRIselect post-ligation clean-up - Part 8.2

These steps were performed as described in Visium Spatial Gene Expression Reagent Kits User Guide CG000239 Rev C (10x Genomics),[13] Steps 4.3 and 4.4: 50 μl of Adaptor Ligation Mix (88 μl Ligation Buffer + 44 μl DNA Ligase + 88 μl Adaptor Oligos) was added to each sample and then incubated for 15 min at 20°C.

Each sample was cleaned-up by addition of 80 μl SPRIselect beads, incubated for 5 min at room temperature. The tubes were then placed on the high magnet position until the solution cleared. Supernatant was discarded and the beads were washed with 125 μl 80% EtOH for 30 s two times. After removing the 80% EtOH, beads were allowed to dry on the magnet for less than 2 min (avoiding over-drying) followed by addition of 30.5 μl Buffer EB to each sample. The samples were removed from the magnet, vortexed, and centrifuged briefly to mix the beads with Buffer EB and were then incubated for 2 min at room temperature. After incubation the samples were placed on the low magnet position until the solution cleared. 30 μl of each sample was transferred to a new tube strip.

Sample index PCR and clean-up - Part 8.3

Samples were indexed. 50 μl Amp Mix and 20 μl of each individual sample index were added to each sample respectively and incubated in the thermocycler as in Visium Spatial Gene Expression Reagent Kits User Guide, Step 4.5, with 8 cycles of PCR: (Step 1; 98°C, 45 s. Step 2; 98°C, 20 s. Step 3; 67°C, 30 s. Step 4; 72°C, 20 s. Step 5; Go to Step 2 for the total of 8 cycles. Step 6; 72°C, 1 min. Step 7; 4°C hold. Heated lid temperature: 105°C, reaction volume: 100 μl).

Subsequently, samples were cleaned up in a double-sided size selection. 60 μl SPRIselect beads were added to each sample and incubated at room temperature for 5 min. Then the tubes were placed on the high magnet position until the solution cleared. Supernatant was transferred to a new tube strip. 20 μl SPRIselect beads were added and incubated at room temperature for 5 min. Then the tubes were placed on the high magnet position until the solution cleared. Supernatant was discarded and the beads were washed with 200 μl 80% EtOH for 30 s twice. After removing the 80% EtOH, beads were allowed to dry on the magnet for about 2 min (avoiding over-drying) followed by addition of 40.5 μl Buffer EB to each sample. The samples were removed from the magnet, vortexed, and centrifuged briefly so the beads were mixed with Buffer EB, then incubated for 2 min at room temperature. After incubation the samples were placed on the low magnet position until the solution cleared. Eluted samples were transferred to a new tube strip.

An additional 0.8x SPRIselect bead clean-up step was performed. 32 μl SPRIselect beads were added to 40 μl sample and incubated at room temperature for 5 min. Then the tubes were placed on the high magnet position until the solution cleared. Supernatant was discarded and the beads were washed with 200 μl 80% EtOH for 30 s twice. After removing the 80% EtOH, beads were allowed to dry on the magnet for about 2 min (avoiding over-drying) followed by addition of 15 μl Buffer EB to each sample. The samples were removed from the magnet, vortexed, and centrifuged briefly so the beads were mixed with Buffer EB, then incubated for 2 min at

room temperature. After incubation the samples were placed on the low magnet position until the solution cleared. Eluted samples were transferred to a new tube strip.

Post-library QC and dilution - Part 8.4

The final libraries were checked by using 1μl sample for quality control on a BioAnalyzer High Sensitivity chip (Agilent). In addition, 2 μl of each sample were used for dsDNA HS Qubit assay (Thermo Fisher Scientific) to determine sample concentration.

Sequencing - Part 9

Libraries were sequenced using Illumina's Nextseq 500. Libraries loading concentration was 1.8 pM with a 1- 5% PhiX spike-in (See Table S2). A total of 4 samples per run, pair-end, dual index sequencing with either; a) 75 cycles High Output and custom primer for Read 2 (Integrated DNA Technologies, sequence: AAG CAG TGG TAT CAA CGC AGA GTA CAT GGG, Purification HPLC) 2 mL of 0.3 μM custom primer for Read 2 loaded into well number 8 of the sequencing cartridge. Read 1: 28 cycles, i7 index: 10 cycles, i5 index: 10 cycles, Read 2: 44 cycles, or b) 150 cycles High Output kits (standard recommended conditions). Read 1: 28 cycles, i7 index: 10 cycles, i5 index: 10 cycles, Read 2: 120 cycles.

We explored the strategy of using a custom primer complementary to the TSO sequence with the aim to read directly into the mRNA insert and thereby avoid reading the 30 bp TSO adaptor sequence and achieve a more cost-effective run. The gained complexity of these sequencing libraries facilitates using a cheaper sequencing kit, e.g., reading 75 cycles instead of 150. It should be noted that since we have compared the data produced by each approach, we would recommend standard sequencing approach b. However, under certain circumstances like dealing with very fragmented mRNA, approach a) could be tried.

Immunofluorescence assay targeting coronavirus spike protein

For immunostaining, we used a SARS-CoV-1 monoclonal antibody, previously shown to cross-react with SARS-CoV-2 in mammalian cell lines.[46] Immunostaining was performed using the aforementioned primary antibody diluted 1:2000 in blocking buffer (3%BSA in 1x PBS) and a goat anti-Mouse IgG (H+L) Alexa Fluor 555 conjugate (Abcam, catalog number A32727) secondary antibody diluted 1:2,000 in blocking buffer. We acquired fluorescence images at 20x magnification using Metafer Slide Scanning platform (MetaSystems). Raw images were stitched with VSlide software (MetaSystems).

TSO-based QC assay protocol

We designed this assay to be performed on a Visium Spatial Tissue Optimization Slide[17], starting by performing **parts 1-5** of our Genome-Wide FFPE Spatial Expression Profiling Protocol, until the reverse transcription reaction is complete. At this point the cassette with the slide was sealed and kept in the fridge overnight.

Tissue removal - Part 6

After reverse transcription we performed tissue removal as described in Step 2.0 in the Tissue Optimization User Guide Rev D CG000238:[17] it should be noted that in step 2.d RT Master Mix is removed instead of Fluorescent RT Master Mix, in step 2.n we left the slide in the pre-warmed 2xSSC - 0.1% SDS buffer for 10 min. In brief, after removing the RT Master Mix, the wells were washed with 100 μl 0.1X SSC, 70 μl Tissue Removal Mix (539 μl Tissue Removal Buffer + 77 μl Tissue Removal Enzyme) was added to each well and incubated at 56°C for 2 h. When the incubation is finished and the permeabilization mix has been pipetted off, the slide is removed from its slide cassette. Next, slides are left for 10 min in pre-warmed 2xSSC - 0.1% SDS buffer (50°C). Then they are immersed 15x in 0.2X SSC and 15x in 0.1X SSC. Finally, they are centrifuged in a slide spinner until dry.

Background imaging - Part 7

Our slides were scanned under an Innoscan 910 (Inopsys) equipment. We selected a laser excitation wavelength of 532 nm, resolution of 5 μm per pixel and Gain 20 and 50 in two successive imaging steps in order to generate two background images at two different gains. It is possible to image slides according to any of the valid imaging guidelines given by the Tissue Optimization User Guide Rev D CG000238.[17]

cy3-TSO hybridization - Part 8

A denaturing step with KOH was introduced to remove the mRNA template, as in Steps 2.1d-g of the Visium Spatial Gene Expression Reagent Kits User Guide CG000239 Rev C (10x Genomics):[13] 75 μl 0.08 M KOH was added on each well to dehybridize template mRNA and incubated for 5 min at room temperature. Next KOH was discarded and the wells were washed once with 100 μl buffer EB per well. Next, we pipetted off the EB buffer and added 75 μl TSO-cy3 probe Mix (162 μl nuclease-free water + 169 μl (20 mM Tris-HCl 2 mM EDTA, 100 mM NaCl buffer) + 6.75 μl 100 μM cy3-TSO probe (Integrated DNA Technologies, Sequence /5Cy3/AA GCA GTG GTA TCA ACG CAG AGT ACA TGG G, Purification HPLC)). The slides were sealed and incubated in a Thermocycler with the following program: Step 1; 75°C, 1 s. Step 2; 63°C, 15 s. Step 3; 23°C, 15 min. Step 4; 4°C, ∞. Heated lid temperature: 80°C, reaction volume: 75 μl. After the incubation we pipetted off the TSO-cy3 probe mix and washed and dried the slide as in the Tissue Optimization User Guide Rev D CG00023817 step 2.1.j onward: (the slide was left for 10 min in pre-warmed 2xSSC - 0.1% SDS buffer (50°C). Then it was immersed 15x in 0.2X SSC and 15x in 0.1X SSC. Finally, it was centrifuged in a slide spinner until dry.

Signal imaging - Part 9

The slides were scanned repeating the same exact settings as in step 7. For background and signal images to be comparable it must be ensured that the scanning settings are the same.

QUANTIFICATION AND STATISTICAL ANALYSIS

Data pre-processing

The raw fastq files containing the cDNA sequences (R2) were pre-processed to remove TSO primer sequences and poly(A) homopolymers using *cutadapt* (v2.8).[66] TSO sequences were trimmed by defining the TSO sequence as a non-internal 5′ adaptor (removes partial or full TSO sequences from the 5′ end) with a minimum overlap of 5 bp and an error tolerance of 0.1. Poly(A) homopolymers were trimmed using a string of 10 A's as a regular 3′ adaptor (removes stretches of poly(A) found anywhere in the sequence as well as the trailing base pairs with a minimum overlap of 5 bp). To search for and trim both adaptor types from the same read sequences we set the–times option to 2.

Data processing

All paired fastq files (after TSO and poly(A) trimming) were processed using spaceranger v1.0.0 together with the corresponding Hematoxylin and Eosin (H&E) stained images in jpeg format. For mapping of the data, we used the mm10-3.0.0 *Mus musculus* reference genome for mouse samples and the GRCh38-3.0.0 *Homo sapiens* reference genome for human samples (both included in the Space Ranger distribution v1.0.0). For the SARS-CoV-2 infected lung tissue samples, we constructed a multiple species reference using the spaceranger mkref command line tool. A GTF formatted annotation file and genome sequence in fasta format were downloaded from NCBI,[67] accession date: may 29, 2020) for the SARS-CoV-2 (wuhCor1) genome (NCBI reference sequence: NC_045512.2). Human annotation file (GTF) and genome sequence (fasta) were downloaded from NCBI, genome build GRCh38.p12 (genome build accession: NCBI:GCA_000001405.27).

Selection of FFPE tissue section for comparison with fresh frozen data

The Fresh Frozen (FF) Visium gene expression dataset (coronal section of one hemisphere of the mouse brain) was downloaded from 10x Genomics website.[20] Using the Allen Brain Atlas as reference, the publicly available FF section was determined to have been collected approximately –2.2 mm from Bregma along the anterior-posterior axis. The FFPE section (coronal section of one hemisphere) was collected at approximately the same coordinate from Bregma along the anterior-posterior axis to obtain the same tissue morphology. This was done by checking the morphology of tissue sections under a bright field microscope every, collected ~100 μm apart and matching the morphology with the Allen Brain Atlas.

Registration of H&E images to the Allen Mouse Brain Atlas anatomical reference with wholebrain

The H&E images of the FFPE and FF tissue sections were registered to the Allen Brain Atlas anatomical reference using the *wholebrain* (v0.1.1) framework in R. Briefly, the H&E images were first converted into grayscale and the intensity values inverted to give the Hematoxylin-stained nuclei higher intensity values than the background (i.e., background is dark and nuclei are bright). These inverted H&E images were then used as input for cell segmentation and registration to the anatomical reference (−2.2 mm from Bregma) by manual addition of correspondence points, i.e., points matching anatomical features of the H&E image and the anatomical reference. A total of 107 correspondence points were defined for the FF H&E image and 116 correspondence points for the FFPE section. The spatial transcriptomics spot coordinates for the FFPE and FF tissue sections were annotated into 11 anatomical regions (fiber tracts, Striatum:STR, Lateral Ventricles:VL, Hypothalamus:HY, Thalamus:TH, Dentate Gyrus:DG-sg, Field CA1:CA1sp, Field CA2:CA2sp, Olfactory areas: OLF, Cortical subplate:CTXsp, Isocortex) using the registered H&E images. First, each Visium spot was artificially represented as a contour (circle) with a pixel radius corresponding to 27.5 μm in the H&E images. The contours (spots) were then annotated based on their overlap with the registered anatomical regions using the get.cell.ids function from the *wholebrain* R package. The annotations returned by the get.cell.ids function represent the most granular division of the brain anatomy according to the brain atlas. To associate a query annotation with a target annotation, we used the ontology provided in the *wholebrain* R package to check whether the target annotation was an ancestor to the query. By repeating this procedure for all anatomical regions returned by the get.cell.ids function, we translated the spot annotations into 11 selected anatomical regions.

Analysis of a FFPE coronal tissue section of the mouse brain

The FFPE Visium data was filtered by (1) removal of spots with fewer than 100 unique genes, (2) removal of mitochondrial protein coding genes and (3) removal of genes annotated to non-coding RNA biotypes ("antisense," "lincRNA," "pseudogenes"). All three filtering steps were computed within an R programming environment using the *STUtility* package (v1.0)[68] and the filtered expression matrix was converted to a Seurat object. Normalization, dimensionality reduction, clustering, UMAP embedding and DE analysis was conducted using the *Seurat* R package (v3.1.5): normalization – SCTransform with variable.features.rv.th = 1.1, variable.features.n = NULL and return.only.var.genes = FALSE; dimensionality reduction – RunPCA; UMAP embedding – RunUMAP with dims = 1:25, n.epochs = 1,000 and n.neighbors = 30; clustering – FindNeighbors with dims = 1:25 and FindClusters with the resolution set to 1.4; DE analysis - FindAllMarkers. The DE analysis was computed using a pairwise Wilcoxon Rank Sum test between the spots in each cluster contrasted to all other spots in the dataset. Only genes with a positive avg_logFC value and an adjusted p value lower than 0.01 were kept in the analysis. For spatial visualizations of the FFPE data, the H&E image was first masked to remove the background using the MaskImages function and then rotated 60° clockwise using the WarpImages from *STUtility*.

Comparison of FFPE and FF mouse brain tissue sections

The FFPE and FF coronal section datasets were first merged and converted into a Seurat object using the InputFromTable function (*STUtility*) followed by filtering, normalization with SCTransform and dimensionality reduction with RunPCA as described in the previous section for the FFPE dataset. Again, for spatial visualizations on the FFPE tissue section, the H&E image was first masked to remove the background using the MaskImages function and then rotated 60° clockwise using the WarpImages from *STUtility*. Biotype annotations were extracted from the GENCODE reference GTF file (GRCh38 genome assembly), shipped with the spaceranger command line tool. These biotype annotations were then used to group genes into biotype groups. In addition, mitochondrial genes (prefixed mt- in MGI nomenclature) were defined as "protein coding mitochondrial" and ribosomal protein genes (prefixed Rpl or Rps in MGI nomenclature) were defined as "protein coding ribosomal." Within each condition (FFPE and FF) the relative amounts of molecules found within each biotype were computed by aggregating all UMI counts within each biotype group and dividing by the total number of UMI counts.

Gene attributes were computed within each condition (FFPE and FF) by summation of UMI counts for each gene (union of genes between FFPE and FF datasets) across all spots (bulk level) followed by log10-transformation (pseudocount 1). The log-transformed values were then used as input for the calculation of a Pearson correlation score using the stat_cor function from the *ggpubr* R package.

Next, we grouped the spots based on the 11 selected anatomical regions (defined using the *wholebrain* registration workflow). An enrichment score was estimated for each gene within a target group (n spots) compared to a background group (m spots). The enrichment score was calculated by multiplying the ratio of averaged gene expression between the target group and the background by the ratio of detection rates between the target group and the background:

$$\textit{enrichment score} = \frac{\frac{1}{n}\sum_{i=1}^{n} x_i + 1}{\frac{1}{m}\sum_{i=1}^{m} x_i + 1} \times \frac{\frac{1}{n}\sum_{i=1}^{n} d(x_i) + 1}{\frac{1}{m}\sum_{i=1}^{m} d(x_i) + 1} \qquad \text{(Equation 1)}$$

Where the function d defines whether or not the gene is present in a spot:

$$d(x) = \begin{cases} \text{i}, & x > 0 \\ 0, & x = 0 \end{cases} \ddot{x} \qquad \text{(Equation 2)}$$

The enrichment scores were estimated for sets of Visium spots covering each of the 11 anatomical regions across the two datasets (FFPE and FF). Then, in order to measure the agreement of gene enrichment scores between anatomical regions across the two datasets, we used a rank-biased overlap estimate (rbo[69]) on the top 1000 genes with the highest enrichment scores. Briefly, the rbo estimate is applied to two ranked lists, where a high value indicates that the lists are similar and a low value indicates that the lists are dissimilar (values range from 0 to 1). The rbo estimate was calculated using an implementation from the *gespeR* R package (v1.22.0). The rank-biased overlap was calculated for each pair of anatomical regions across the two datasets (FFPE and FF) to produce a similarity matrix shown in Figure 2D.

Visualization of marker gene expression by ISH

One candidate marker gene was selected from each cluster defined in the FFPE dataset and queried in the ISH data explorer of the Allen Mouse Brain Atlas. The selection of marker genes was done manually based on adjusted p value, high avg_logFC values and also based on presence in the ISH atlas. ISH data (microscopy images) of coronal tissue sections were selected from the data explorer at a distance from Bregma along the anterior-posterior axis matching approximately that of the FFPE and FF tissue sections (−2.2mm) and downloaded in full resolution from the High-Resolution Image Viewer. Gene expression patterns (normalized expression) from the FFPE Visium data were visualized side by side with the ISH images.

Cell type mapping with stereoscope

To assess how certain cell types were distributed in the tissue sections (both FF and FFPE) we used the tool *stereoscope* (v.3.0)[70] to integrate scRNA-seq data obtained with the SMART-seq protocol.[24] The area of a capture location (spot) in the spatial assay is large enough to host several cells; a common estimate is 1-10 cells per spot. Hence, the observed gene expression at each spot can be considered a mixture of contributions from multiple cells. Furthermore, the cell population associated with a spot is not necessarily homogeneous, meaning different cell types may be represented and a one-to-one relationship between spot and cell type is not guaranteed. Thus, to make an informed statement regarding certain cell types' arrangement within the tissue, based on the spatial transcriptomics data, one must first *deconvolve* the gene expression profiles. The method we used, *stereoscope,* addresses the issue of mixed contributions by leveraging "pure" single cell data (one datapoint represents one cell and type) to first characterize the expression profile of each cell type and then estimate the composition of cell types that best explain the observed gene expression at each spot using these profiles. It is a probabilistic approach, where both single cell and spatial data are modeled as negative binomial distributed. The output from *stereoscope* is a matrix in the format of [n_spots]x[n_types] where each element represents a proportion estimate (not a score) of a given cell type at a specific spot. Any cell type with less than 25 members was excluded from the

analysis, all cells from types with more than 25 but less than 500 members were used, 500 cells were randomly sampled from types with more than 500 members. A batch size of 2,048 and 7,5000 epochs was used in both steps of the procedure. We used the 5,000 most variable genes (in the single cell data) in the analysis.

Analysis of multiple FFPE coronal sections from the mouse brain

The 7 FFPE coronal section datasets (shown in Figure S9) were first merged and converted into a Seurat object using the InputFromTable function from the *STUtility* R package followed by normalization with SCTransform. The normalization step was computed with default settings. For data integration with *harmony*, the datasets were grouped by experimental condition and the input assay set to "SCT." Next, the harmony embedding matrix was used as input for UMAP to embed the spots into an integrated 2D representation (Figure S9B) using the RunUMAP function from the *Seurat* R package (reduction = "harmony"). Cluster compositions were computed by calculating the frequency of each cluster within each of the three experimental batches and represented as a bar chart (Figure S9C). Lastly, the spot expression profiles were color coded to highlight similarities in gene expression across the three experimental batches (Figure S9D). The color encoding was done by computing a 3D embedding of the spot expression profiles using the harmony embedding matrix as input for UMAP (RunUMAP function from *Seurat*, reduction = "harmony," n.components = 3). The 3D embedding was then rescaled to the unit cube, i.e., each of the UMAP vectors were rescaled to range between 0 and 1. The rescaled vectors were further leveraged as color intensities using the rgb function from the *grDevices* R package, resulting in a color defined in sRGB color space for each spot.

Analysis of lung and kidney organoids

Four lung organoid Visium datasets and three kidney organoid Visium datasets were first merged, respectively, into two expression matrices. These datasets were subsequently filtered by (1) removal of spots with fewer than 500 unique genes, (2) removal genes with a total UMI count lower than 10 and (3) removal of genes annotated by a non-coding RNA biotype together with mitochondrial protein coding genes and ribosomal protein coding genes. All three filtering steps were computed within an R programming environment using the InputFromTable function from the *STUtility* package (v1.0)[68] and the filtered expression matrices were converted into Seurat objects. Since each dataset covered multiple organoid tissue sections, each organoid was first annotated by manually selecting and labeling spots (ManualAnnotation, *STUtility*). Next using the spot coordinates defined for each organoid, a square crop window was created to cover the tissue of each organoid, specified as the offset of the square along the x and y axes along with the width and height of the square. The crop windows were then used to subset the gene expression data from each organoid with the cropped H&E images as background using the CropImages function from *STUtility*. This process was repeated for both the lung and kidney organoid datasets. Normalization was conducted using SCTransform with default parameter settings, followed by dimensionality reduction by PCA and UMAP (RunUMAP, dims = 1:20). Unsupervised clustering was computed by constructing a shared nearest neighbor graph (FindNeighbors, reduction = "pca," dims = 1:20) followed by modularity optimization (FindClusters) with the resolution parameter set to 0.3. DE analysis was performed on the clusters using FindAllMarkers and the top 10 genes with lowest adjusted p values and a positive average log fold-change were selected for visualization.

Filtering and normalization of carcinosarcoma (HGSC) datasets

Raw expression matrices from the four gynecological carcinosarcoma tissue samples were first merged and then filtered to (1) remove spots with fewer than 150 unique genes, (2) remove genes with fewer than 10 counts across the whole dataset and (3) remove mitochondrial protein coding genes as well as all non-coding RNA biotypes ("antisense," "lincRNA," "pseudogenes"). All three filtering steps were computed within an R programming environment using the *STUtility* package and the filtered expression matrix was converted to a Seurat object. The raw counts were normalized using the Variance Stabilizing Transformation (VST) method implemented in the SCTransform function in the *Seurat* package (v3.1.5) (settings: return.only.var.genes = FALSE, variable.features.n = NULL, variable.features.rv.th = 1.1).

Non-negative Matrix Factorization and pathway analysis of carcinosarcoma (HGSC) datasets

The VST normalized and scaled expression matrix was decomposed into 15 factors using a Non-negative Matrix Factorization method from the *NNLM* package modified as described by Wu et al.[37] and implemented in the RunNMF function in *STUtility*. In summary, the normalized and scaled gene expression matrix (A) is first transformed to contain strictly non-negative values and is then decomposed into two matrices W*H, where W is the (genes x samples) gene loadings matrix and H is the (factors x samples) spot embeddings matrix. From the gene loadings matrix, we selected a set of top contributing genes for each factor to use as input for pathway analysis using a simple mean and standard deviation threshold. First, each factor gene loading vector (gene weights) was log-transformed, and from this vector, the mean and standard deviation was estimated. The threshold used to define top contributing genes was then set to keep genes with a log-transformed gene loading value higher than 1.645 standard deviations from the mean. Each set of "top contributor" genes were used as input for pathway analysis using the gost function from the *gprofiler2* R package with two different sources; *GO:BP* and the "cancer hallmark collection" available from MSigDB.[71] To summarize, gprofiler2 provides an interface for functional enrichment analysis of query gene lists, with the option to include multiple different sources. The function gost returns a set of pathways associated with the query gene list defined by an overrepresentation statistic (adjusted p value threshold set to 0.05).

Analysis of lung tissue infected with Covid-19

Two lung tissue FFPE Visium datasets were filtered by (1) removal of spots with fewer than 100 unique genes, (2) removal of mitochondrial protein coding genes and (3) removal of genes annotated to non-coding RNA biotypes (“antisense,” “lincRNA,” “pseudogenes”). All three filtering steps were computed within an R programming environment using the *STUtility* package and the filtered expression matrix was converted to a Seurat object. Normalization was conducted using the SCTransform function from the *Seurat* R package. The normalized expression data was decomposed into 15 factors using the RunNMF function from *STUtility.* Next, the Seurat object was reduced into two subsets of covering the bronchus tissue and the alveolar region. The selection was done by manually defining cropping windows of the two regions followed by subsetting using the CropImages function from the *STUtility* R package.

Differentially expressed marker genes were downloaded in tabular format from a molecular cell atlas of the human lung.[48] Cell type markers were selected for a subset of 25 cell types (Figure S18) and further filtered to include genes with an average log-fold change of at least 0.8 and an adjusted p value smaller than 0.01. The filtered sets of cell type markers were then used to compute Area Under the Curve (AUC) scores to quantify the activity of each cell type in the spatial transcriptomics data. First, a gene-expression rankings matrix was constructed using the AUCell_buildRanking function from the *AUCell* R package,[49] using the vst normalized gene expression matrix as input. Each cell type marker gene set was then used to compute the AUC scores using the AUCell_calcAUC function (*AUCell* R package) using the top 5% of the genes in the ranking matrix, thus providing a score for each cell type and spot. To summarize, the AUC score estimates the enrichment of a gene set among the top 5% most highly expressed genes in a spot. In order to correlate the cell type AUC scores with the factor activities, we first applied a log-transformation with a pseudocount of 1 (log1p) to the AUC scores and factor activity values to achieve normality. Next, we computed the Pearson correlation coefficient for all pairs of log-transformed cell type AUC scores and log-transformed factor activity vectors (Figure S18).

Immunofluorescence assay targeting coronavirus spike protein image analysis

For image analysis, nuclei were segmented and counted using fluorescence images of the lung tissue stained with DAPI. The cell segmentation process was conducted using the *EBImage* R package (v4.30). Briefly, the DAPI image (TIFF formatted) was converted into grayscale and the intensity values were normalized by linear scaling to fit a range between 0 and 1. The normalized image was then thresholded using a moving square window with a side length of 5 pixels and with the offset set to 0.05. Thereafter, the thresholded image was transformed using the opening function from *EBImage* which applies a sequence of morphological operations to remove background noise (kern was specified by a disc shaped brush with a size of 5). Next, fillHull (EBImage) was applied to fill holes within sets of connected pixels followed by labeling of connected pixel sets. The total number of connected pixel sets were then used as an estimate of the total number of cells (excluding erythrocytes) in the tissue. A more detailed introduction to the cell segmentation workflow can be found in the introductory vignette provided in the EBImage R package.[72]

Quantification of Covid-19 positive cells was conducted as follows. First, raw images were processed in ImageJ (version 2.1.0/1.53c) where nuclei (counterstained with DAPI) and SARS1 signal channels were merged together. We used a manual approach for SARS-CoV-1 signal quantification and visualization in Adobe Photoshop (version 19.0.0), as automated signal detection was difficult due to background noises. For visualization, the color balance was adjusted (red: −30, green: +100, blue: −30) to reduce the background noises to the level that made it possible to detect those presumably true bright signals surrounding the nuclei. A 5x7 guide layout was set to aid counting per square area.

Archival FFPE tissue quality assessment

A total of 24 Visium datasets collected mouse brain, HGSC, PFA organoids, lung tissue infected with Covid-19 and four archival cancer specimens were loaded and converted into Seurat objects using InputFromTable (STutility) with default settings. The number of unique genes per spot and UMIs per spot were selected as quality metrics to assess degradation of tissues with respect to storage time. Distributions of these quality metrics were visualized as violin plots grouped by storage time in ascending order.

Cell Genomics, Volume *1*

Supplemental information

Genome-wide spatial expression profiling in formalin-fixed tissues

Eva Gracia Villacampa, Ludvig Larsson, Reza Mirzazadeh, Linda Kvastad, Alma Andersson, Annelie Mollbrink, Georgia Kokaraki, Vanessa Monteil, Niklas Schultz, Karin Sofia Appelberg, Nuria Montserrat, Haibo Zhang, Josef M. Penninger, Wolfgang Miesbach, Ali Mirazimi, Joseph Carlson, and Joakim Lundeberg

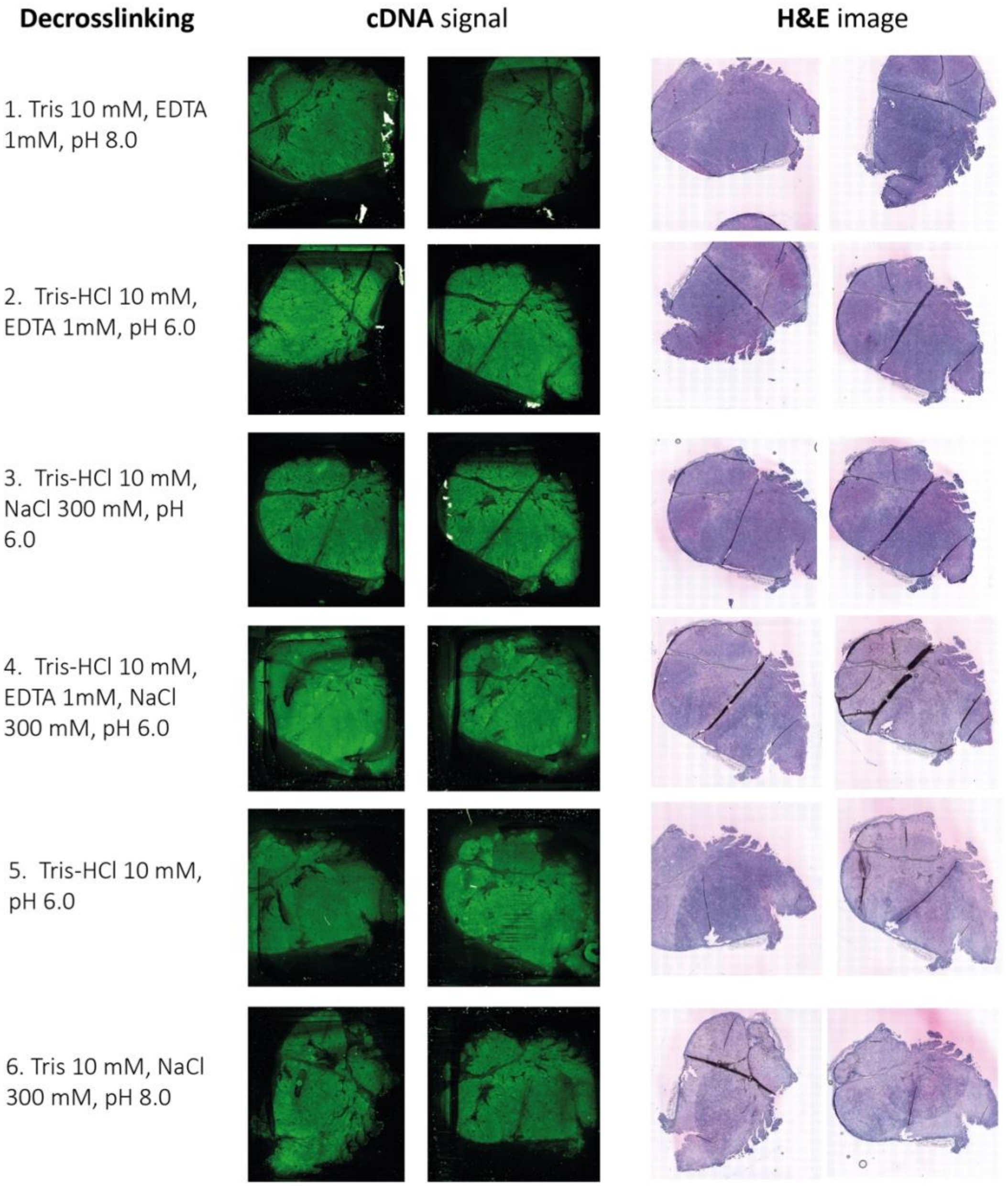

Figure S1. Evaluation of decrosslinking buffer conditions, related to STAR Methods.

Six different buffers with variable pH, EDTA and NaCl concentrations were evaluated using a tissue optimization (TO) assay on FFPE tissue sections. In summary, cDNA footprints are generated through incorporation of Cy3 fluorescently labelled nucleotides by second strand synthesis, where signal intensity is approximately proportional to cDNA concentration on the surface (middle column). No apparent difference was observed in the cDNA signal generated using the six different buffers. H&E images of the tissue sections are shown in the right column.

Occasional variation in the Cy3 signal, for example the darker bands visible in condition 4, are likely produced in the array printing process of the TO slides.

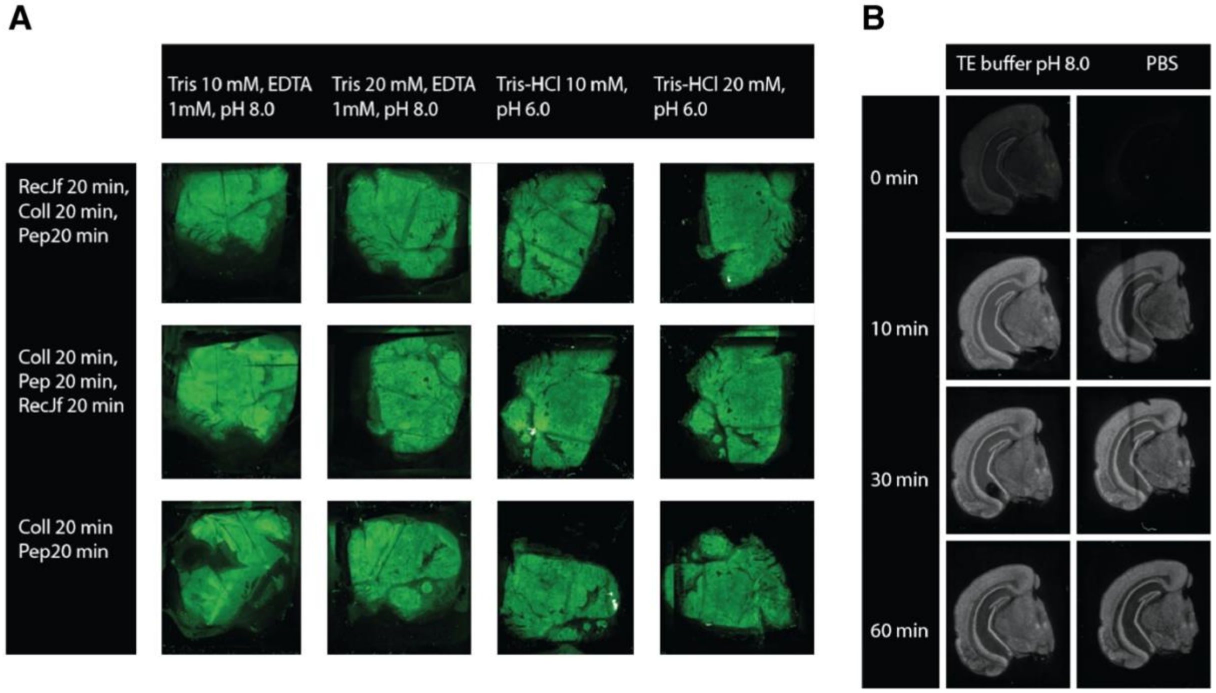

Figure S2. Evaluation of decrosslinking buffers and permeabilization conditions, related to STAR Methods.

cDNA footprints are generated through incorporation of Cy3 fluorescently labelled nucleotides by second strand synthesis. Signal intensity is approximately proportional to cDNA concentration on the surface.

(A) Evaluation of crosslink buffers for cDNA synthesis on FFPE tissue sections. Four crosslink buffers were tested (columns) in combination with three permeabilization conditions (rows). No apparent difference in cDNA signal was observed between the conditions.

(B) Pepsin treatment optimization on Mouse Brain tissue sections. TE buffer pH 8.0 (left column) was compared with PBS buffer (right column) at four different permeabilization times. Variation in the Cy3 signal observed in PBS 10 min and PBS 30 min (dark band), is likely an artefact produced in the array printing process of the TO slides.

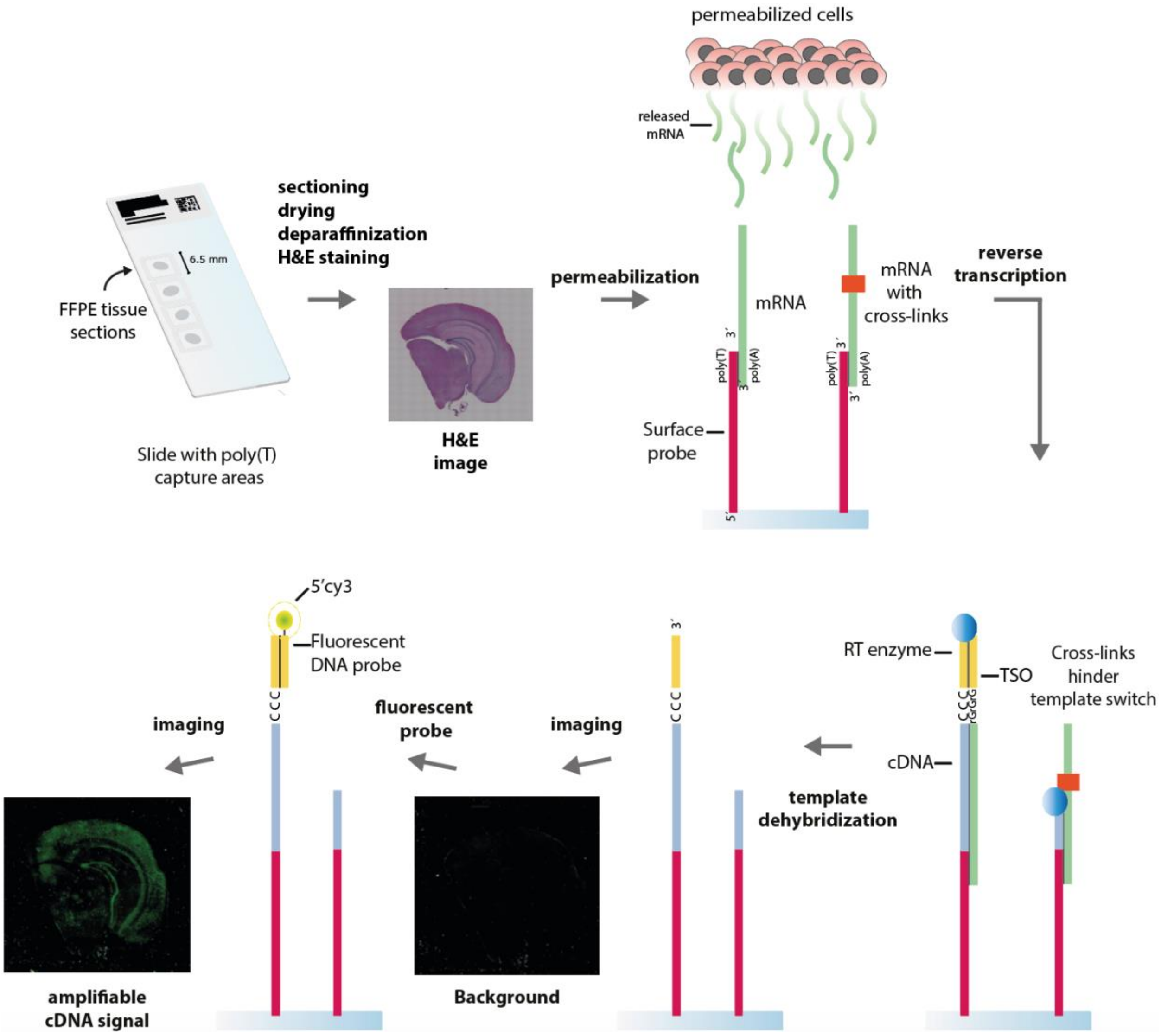

Figure S3. TSO-based QC assay for FFPE samples, related to STAR Methods.

A slide with polyT probes is used, sections of tissue are placed on the capture areas. The FFPE sections are dried, deparaffinized, stained with H&E and imaged under a high-resolution microscope. During permeabilization, the mRNA molecules diffuse outside the cell and their poly(A) tails hybridize the poly(T) of the surface probes. During reverse transcription a cDNA strand is synthesized and a complementary to TSO sequence added in the 3´end. Then the tissue is removed and the mRNA templates are de-hybridized. Slides are imaged to ensure there is no

tissue left over the slide. Finally, a fluorescent cy3-TSOprobe is hybridized and the cDNA signal image is taken.

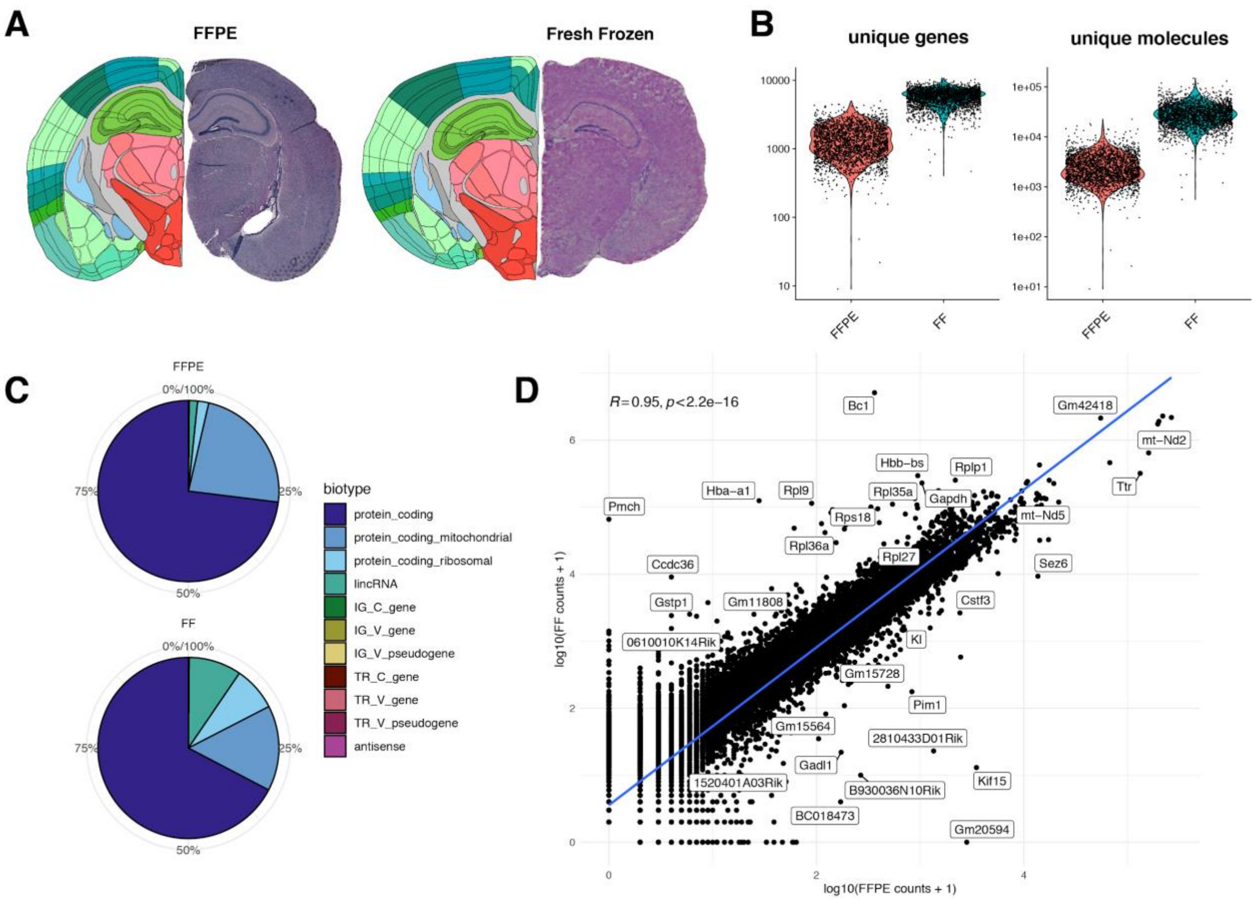

Figure S4. Image registration, quality control and correlation of FFPE and FF tissue sections, related to Figure 2 and STAR Methods.

(A) H&E images of the FFPE and Fresh Frozen (FF) tissue sections (right hemisphere) next to the registered anatomical reference from the Allen Mouse Brain Atlas (left hemisphere). The registration was done using the *wholebrain* framework with the stereotactic coordinate set to -2.2 from bregma along the anterior-posterior axis for both tissue sections.

(B) Quality metrics shown as violin plots for the FFPE and FF sections (y-axis in log-scale). The median number of unique genes per spot was ~1200 and ~6000 respectively. The median number of unique molecules (UMIs) per spot was ~2200 for the FFPE section and ~27200 for the FF section.

(C) Composition of RNA biotypes in the two tissue sections. Notably, the FFPE section contained a higher fraction of mitochondrial protein coding genes and a lower fraction of both ribosomal protein coding genes as well as lncRNA.

(D) Scatter plot showing the correlation between log10-transformed UMI counts per gene for two tissue sections with a Pearson correlation coefficient of 0.95. Highlighted outlier genes indicate that hemoglobin genes *Hba-a1* and *Hbb-bs* and certain ribosomal protein genes are more highly expressed in the fresh frozen tissue section.

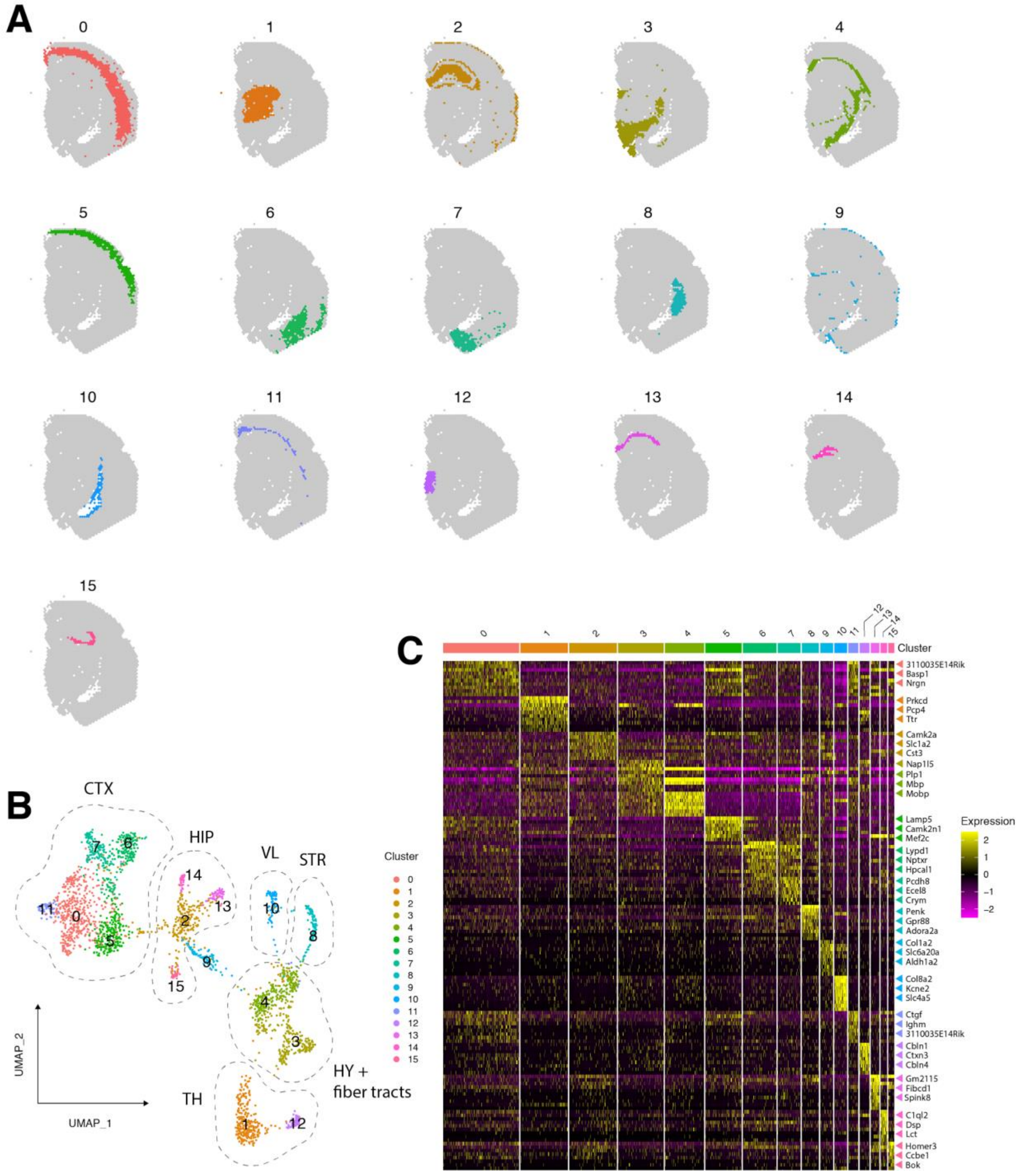

Figure S5. Clustering and marker detection in a coronal section from FFPE mouse brain tissue, related to Figure 2 and STAR Methods.

(A) Spatial mapping of 16 clusters on tissue section spot coordinates.

(B) 2D UMAP embedding of spatial gene expression data colored by cluster. Clusters 2, 13, 14 and 15 form the hippocampal region (HIP) where cluster 13 maps to field 1 of the pyramidal layer (CA1sp), cluster 14 maps to the dentate gyrus (DG-sg) region and cluster 15 maps to field 3 of

the pyramidal layer (CA3sp) region. Cluster 2 shows a less specific pattern in **A** where it makes up the majority of the HIP and also part of the outermost layer of the cerebral cortex (CTX). This can also be observed in the UMAP where cluster 2 connects the HIP to CTX. Clusters 0, 5, 6, 7 and 11 form the CTX which can be further divided into cortical subplate and olfactory areas (6, 7) and the isocortex (0, 5, 11). Other examples include: hypothalamus (HY) – cluster 3; fiber tracts – cluster 4; thalamus (TH) – clusters 1 and 12; striatum (STR) – cluster 8; lateral ventricle (VL) – cluster 10. Cluster 9 maps mostly to the edges of the CTX and is enriched for genes expressed primarily in vascular tissue.

(C) Heatmap of differentially expressed genes within each cluster, represented by normalized and scaled expression values. Three selected genes per cluster with a high significance (adjusted p-value < 0.01) are highlighted for each cluster on the right side of the heatmap.

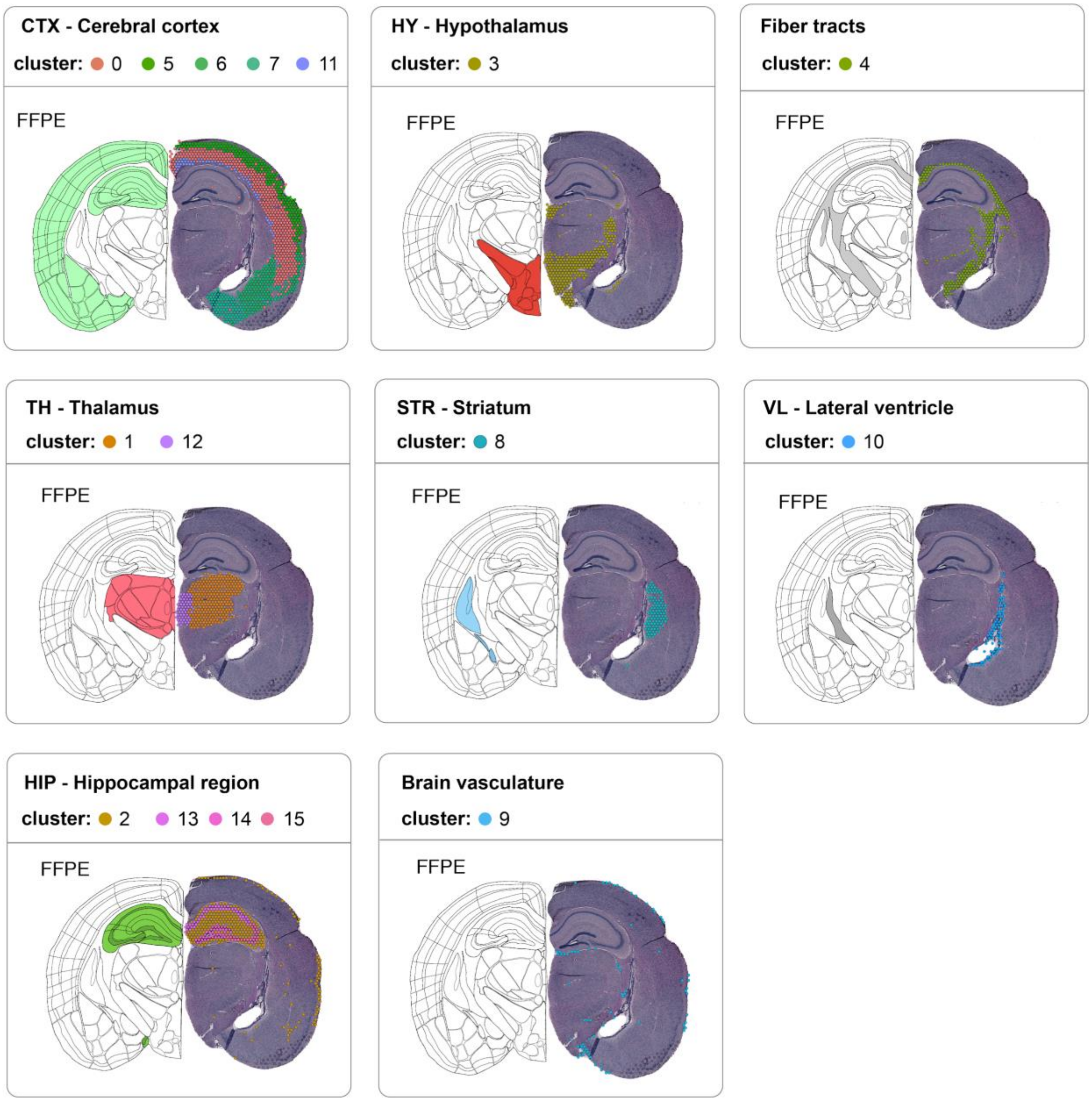

Figure S6. FFPE coronal section clusters agreement with anatomical regions, related to Figure 2 and STAR Methods.

Clusters have been grouped together based on their co-localization with anatomical landmarks from the Allen Mouse Brain Atlas.

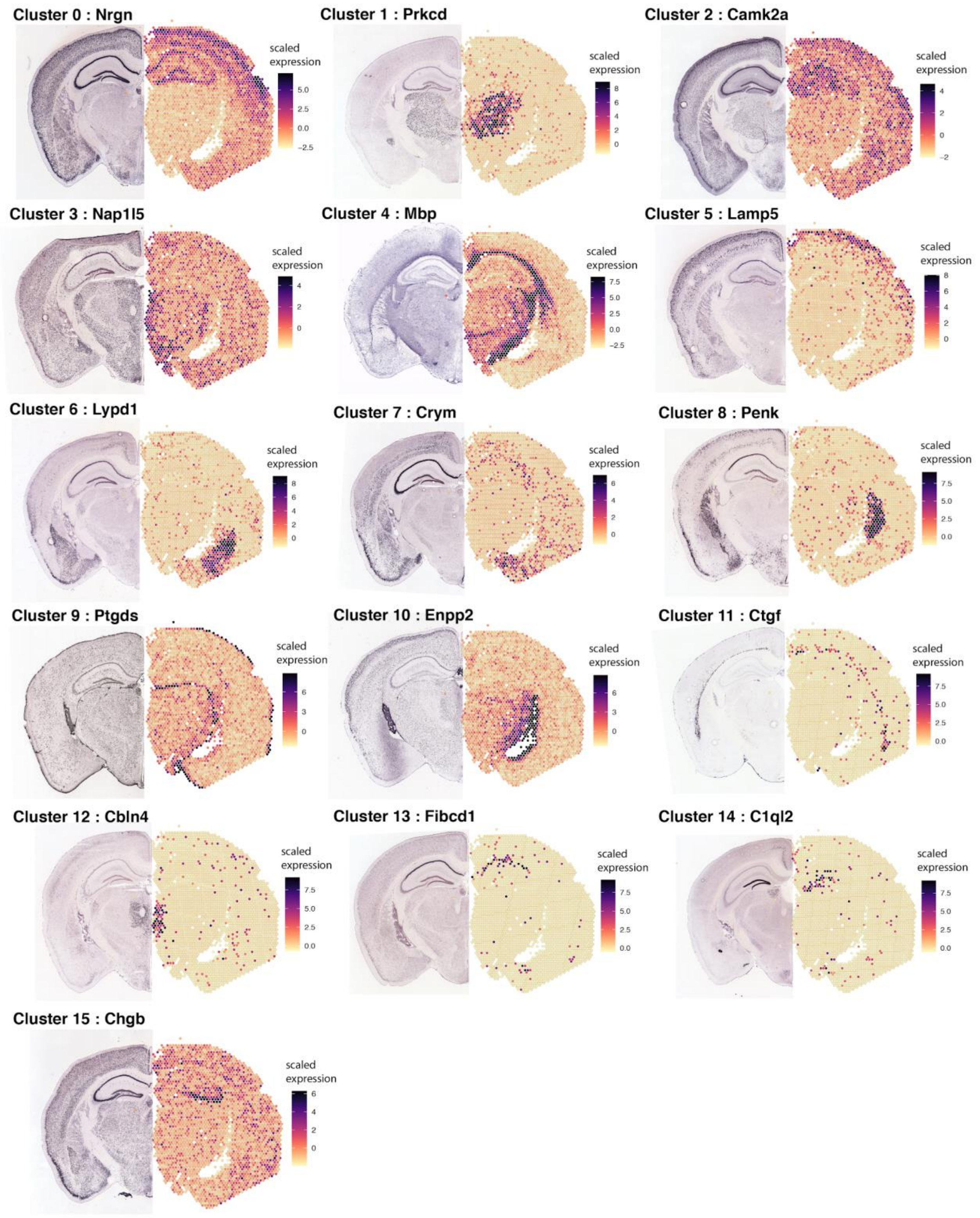
Cluster 0 : Nrgn
scaled expression
5.0
2.5
0.0
−2.5
Cluster 1 : Prkcd
scaled expression
8
6
4
2
0
Cluster 2 : Camk2a
scaled expression
4
2
0
−2
Cluster 3 : Nap1l5
scaled expression
4
2
0
Cluster 4 : Mbp
scaled expression
7.5
5.0
2.5
0.0
−2.5
Cluster 5 : Lamp5
scaled expression
8
6
4
2
0
Cluster 6 : Lypd1
scaled expression
8
6
4
2
0
Cluster 7 : Crym
scaled expression
6
4
2
0
Cluster 8 : Penk
scaled expression
7.5
5.0
2.5
0.0
Cluster 9 : Ptgds
scaled expression
6
3
0
Cluster 10 : Enpp2
scaled expression
6
3
0
Cluster 11 : Ctgf
scaled expression
7.5
5.0
2.5
0.0
Cluster 12 : Cbln4
scaled expression
7.5
5.0
2.5
0.0
Cluster 13 : Fibcd1
scaled expression
7.5
5.0
2.5
0.0
Cluster 14 : C1ql2
scaled expression
7.5
5.0
2.5
0.0
Cluster 15 : Chgb
scaled expression
6
4
2
0

Figure S7. Validation of marker genes by ISH, related to Figure 2 and STAR Methods. Selected marker genes visualized by *in situ* hybridization (ISH) from the Allen Mouse Brain Atlas (left hemisphere) versus scaled expression values (scaled pearson residuals) from our FFPE data (right hemisphere). Marker genes were selected based on significance (adjusted p-values) and average log-fold change from the differential expression analysis.

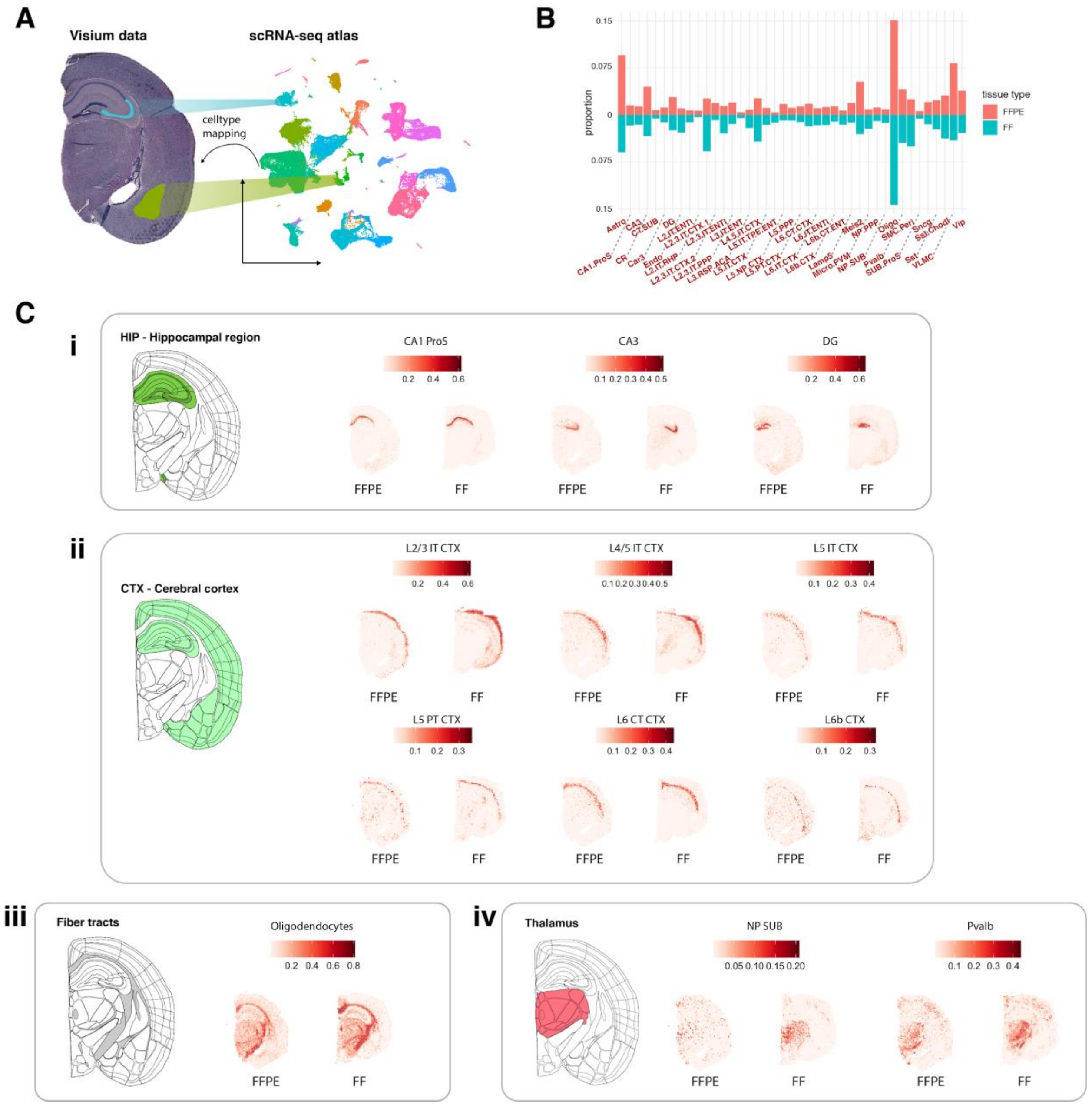

Figure S8. Cell type subclass mapping with stereoscope, related to Figure 2 and STAR Methods.

(**A**) Illustration of cell type mapping using spatially resolved transcriptomics data from a FFPE and FF coronal mouse brain section (left) and a SMART-seq scRNA-seq data from the Allen Brain Atlas (right), with a total of 41 subclasses. Cell type proportions are estimated from the mixed gene expression profiles of each spot in the spatially resolved transcriptomics dataset and can be visualized on the H&E image to define the spatial location of each cell type. **(B)** Summed proportion estimates for the FFPE and FF datasets. Red bars correspond to the summed cell type

proportions in the FFPE data and blue bars correspond to the summed cell type proportions in the FF data. Cell type subclass abbreviations are listed on the x axis. **(C)** A selected subset of cell types visualized by their relative proportions which co-localize with the hippocampal region (**i**), cerebral cortex (**ii**), fiber tracts (**iii**) and the Thalamus (**iv**). Color bars represent cell type proportions. **i)** Three distinct cell types co-localized with the HIP; CA1 ProS mapped to field 1 of the pyramidal layer (CA1-sp); CA3 mapped to field 3 of the pyramidal layer (CA3-sp) and DG mapped to the dentate gyrus (DG-sg). **ii)** Six cell types mapped to layers of the CTX. **iii)** Oligodendrocytes mapped to the fiber tracts. **iv)** Pvalv (parvalbumin) subclass and near-projecting subiculum (NP-SUB) subclass mapped to the thalamus.

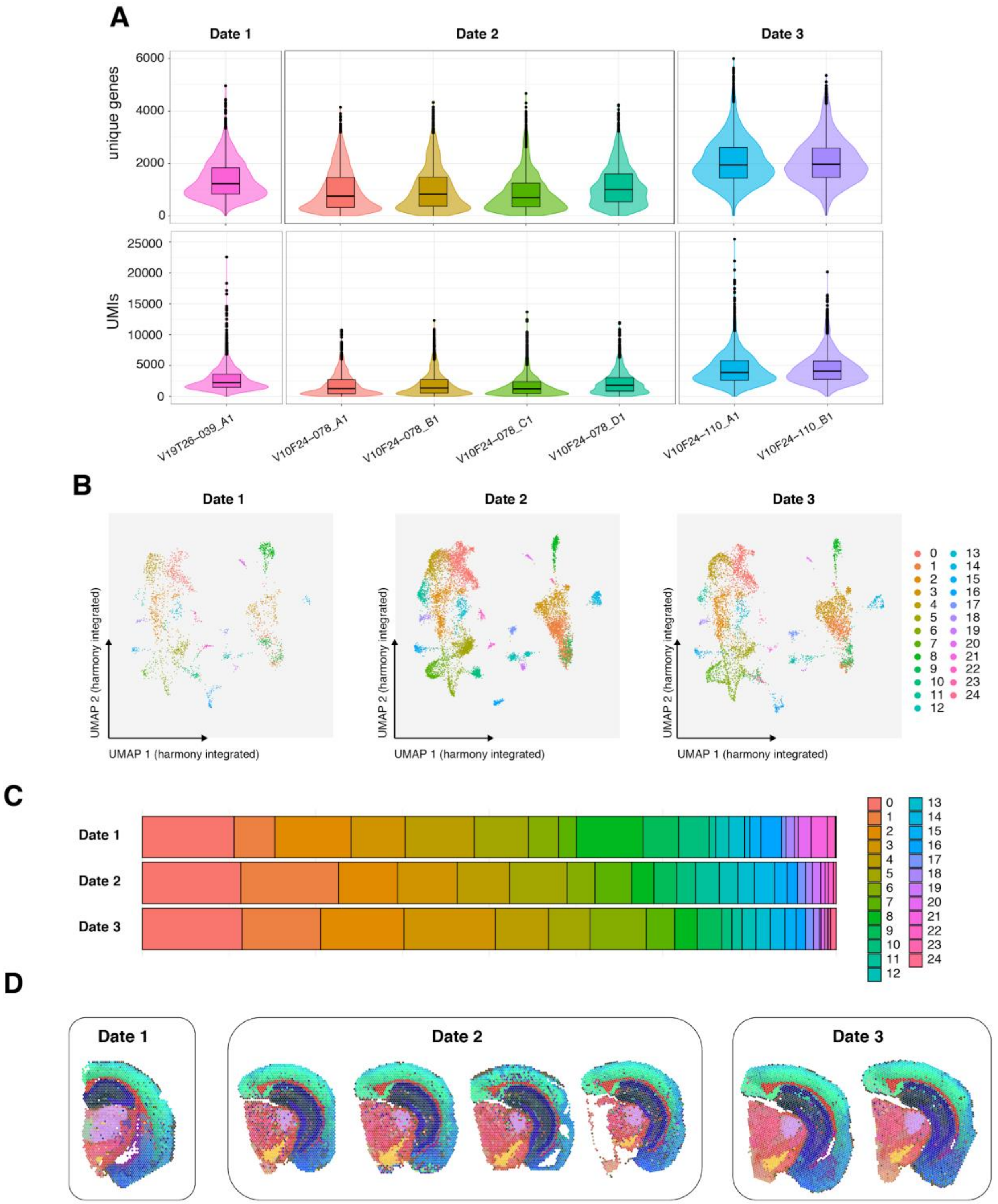

Figure S9. Spatial transcriptomics across multiple coronal mouse brain tissue sections, related to Figure 2 and STAR Methods.

(A) Quality metrics summarized for 7 coronal mouse brain datasets obtained at three different time points; unique genes (top panel) per spot and number of UMIs (bottom panel) per spot.

(B) UMAP embedding and clustering results (datasets integrated with harmony) of spot expression profiles split by time point.

(C) relative composition of clusters in each time point.

(D) Spatial visualization of expression profiles encoded as RGB colors.

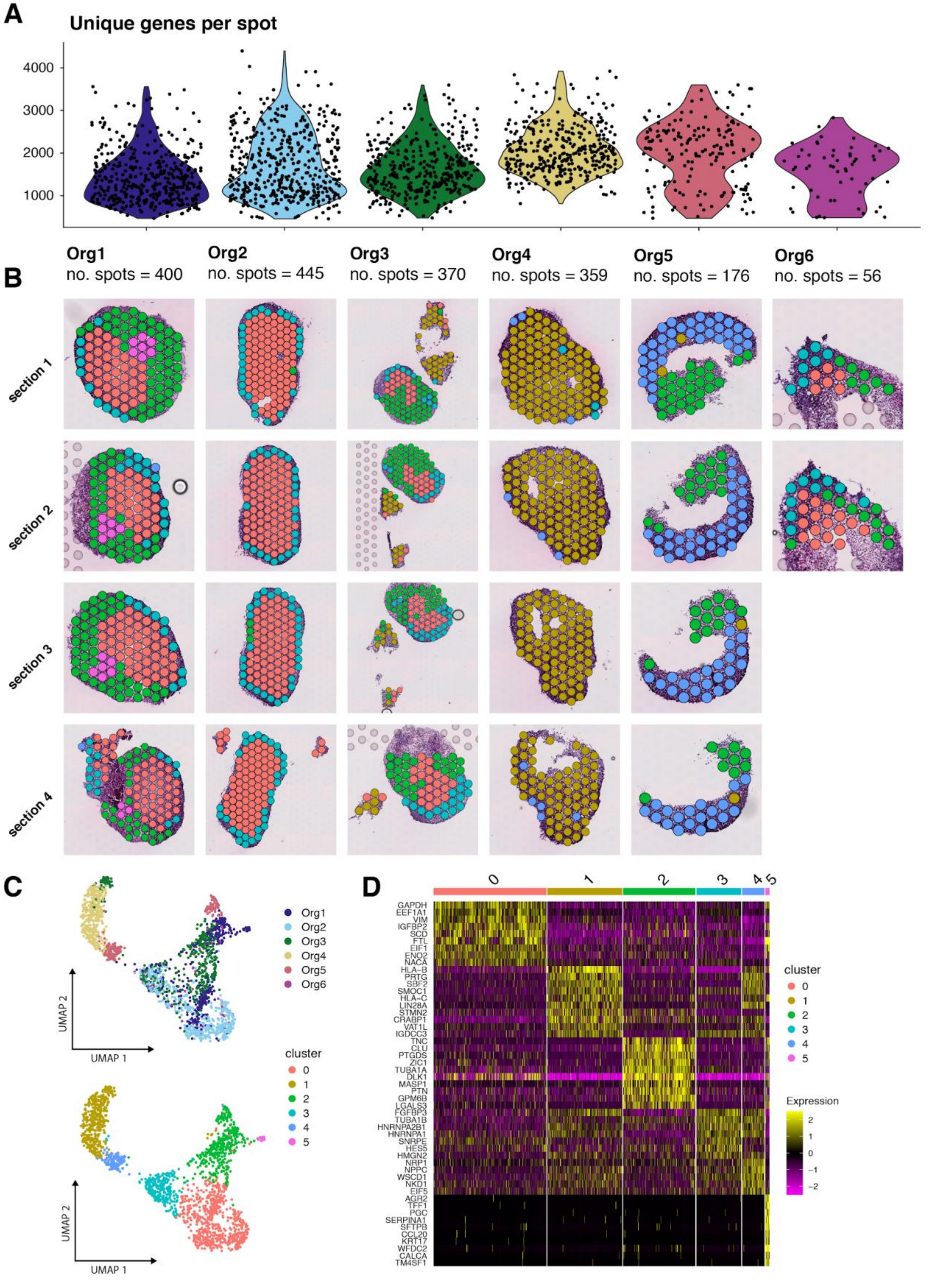

A
Unique genes per spot
Org1 no. spots = 400
Org2 no. spots = 445
Org3 no. spots = 370
Org4 no. spots = 359
Org5 no. spots = 176
Org6 no. spots = 56
B
section 1
section 2
section 3
section 4
C
UMAP 1
UMAP 2
cluster
D
Expression

Figure S10. Lung organoids overview, related to STAR Methods.

(A) Violin plot of unique genes per spot distributions across 6 lung organoids.

(B) Clusters overlaid on H&E images of replicate tissue sections for each of the 6 organoids (4 replicate sections for organoids 1-5 and 2 replicate sections for organoid 6).

(C) UMAP embedding of spot transcriptomic data colored by organoid (top) and clusters (bottom).

(D) DE heatmap showing the top 10 markers per cluster.

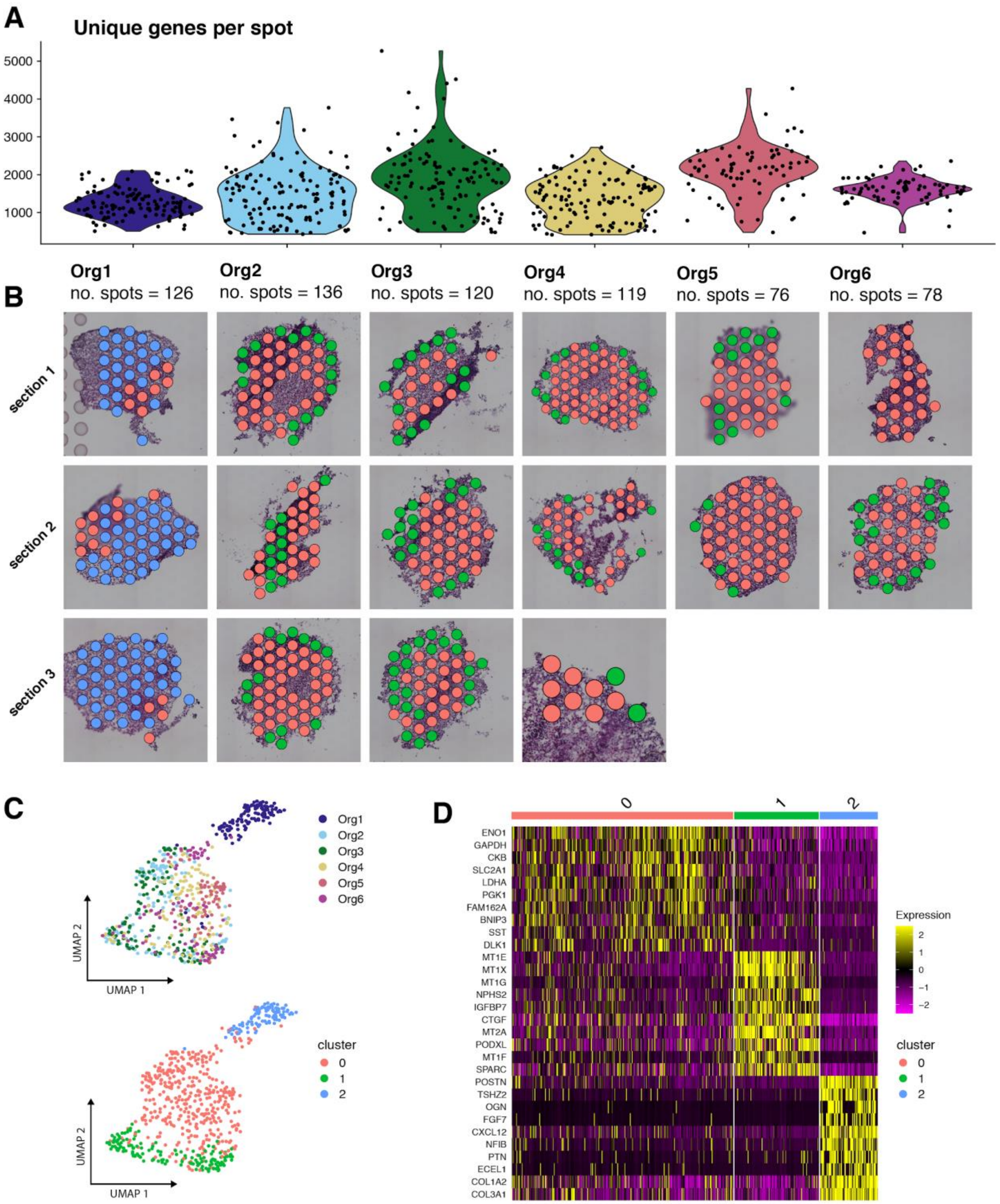

Figure S11. Kidney organoids overview, related to STAR Methods.

(A) Unique genes per spot distributions across kidney 6 organoids.

(**B)** Clusters overlaid on H&E images of replicate tissue sections for each of the 6 organoids (3 replicate directions for organoids 1-4 and 2 replicate sections for organoids 5-6).

(**C)** UMAP embedding of spot transcriptomic data colored by organoid (top) and clusters (bottom).

(D) DE heatmap showing the top 10 markers per cluster.

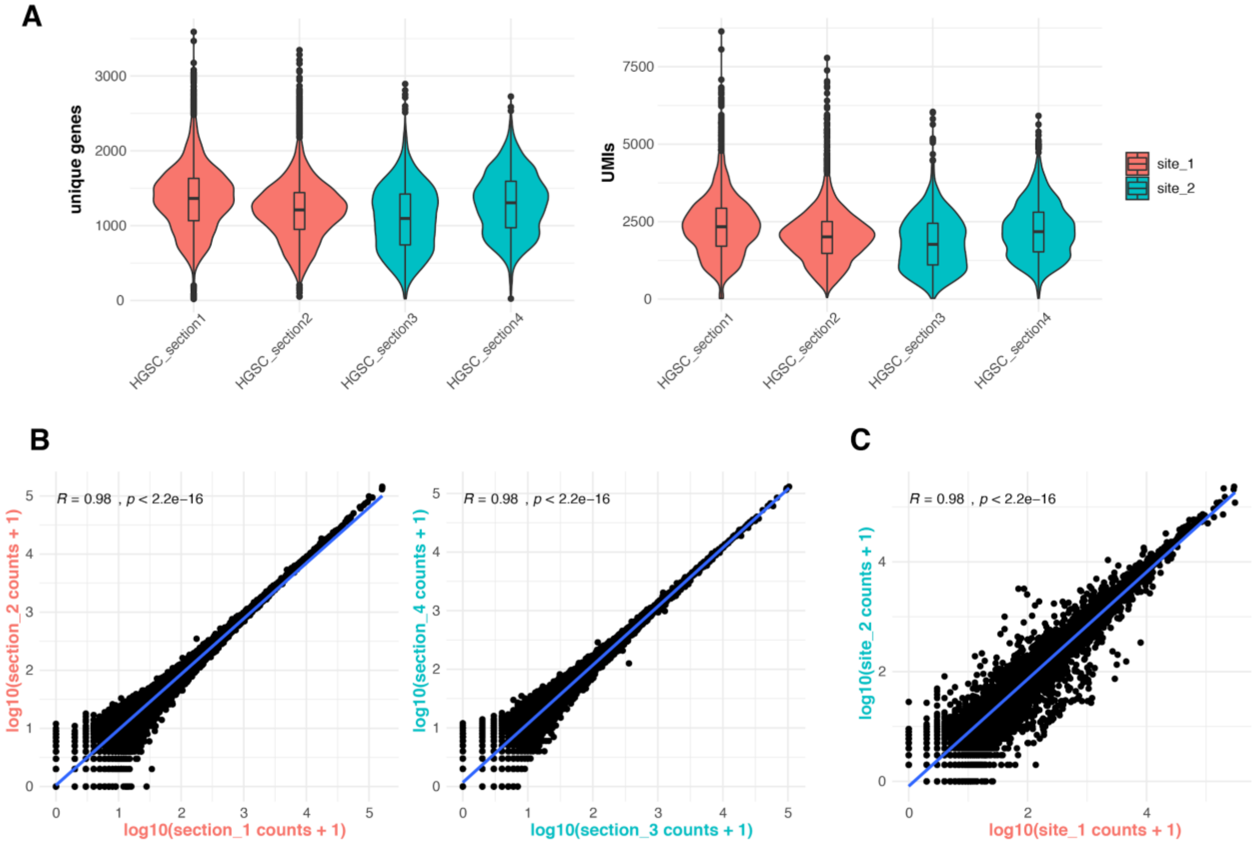

Figure S12. Ovarian carcinosarcoma data, related to Figure 3 and STAR Methods.

(A) Violin plots representing the number of genes per tissue-covered spot and the number of UMIs per tissue-covered spot respectively. Plots with similar color are technical replicates from the same FFPE block

(B) Gene-gene correlation plots between adjacent sections corresponding to each FFPE block showing R = 0.98 in both, indicating low technical variance.

(C) Gene-gene correlation plot between the two FFPE blocks.

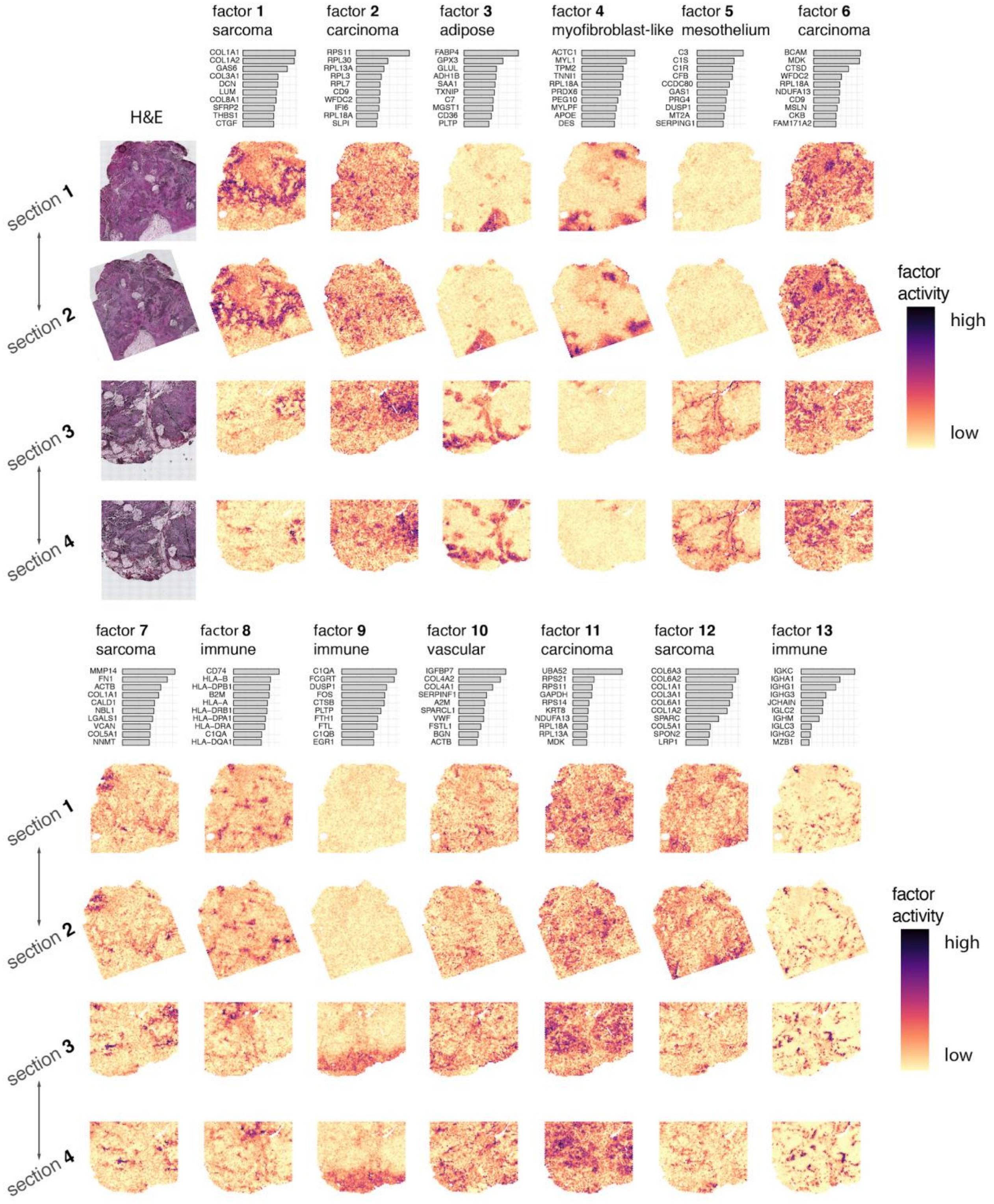

Figure S13. Spatial factor activity maps of four carcinosarcoma tissue sections, related to Figure 3 and STAR Methods.

The first column shows the H&E images linked to each spatial activity map. The remaining columns represent factor activity maps and consist of four rows, with one row for each tissue section. Adjacent sections are indicated by double headed arrows. The bar charts on top of each activity map shows the top 10 contributing genes for each factor. Factors 14 and 15 were driven mainly by ribosomal protein coding genes and were therefore omitted.

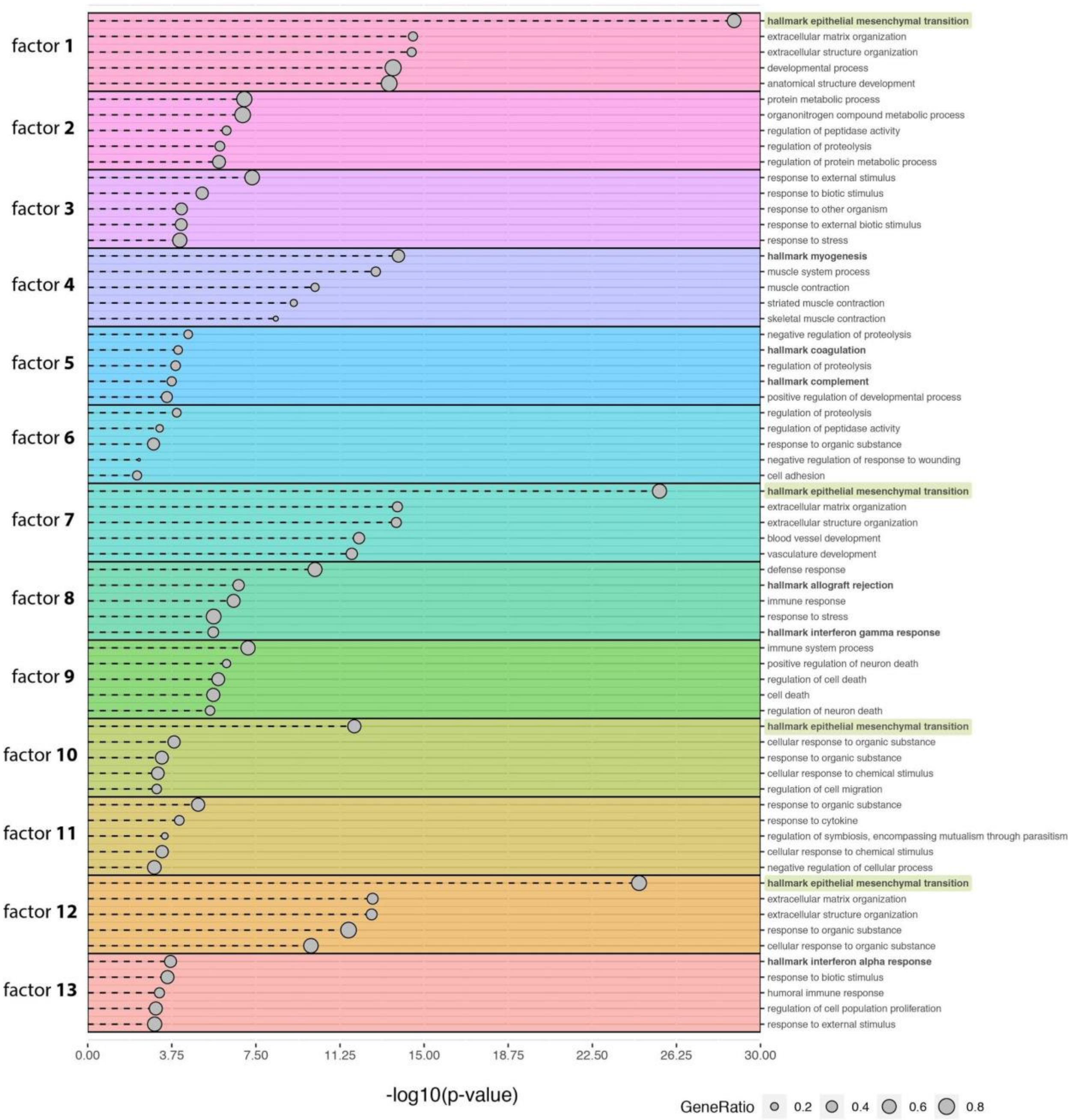

Figure S14. Pathway analysis of factors in carcinosarcoma tissue, related to Figure 3 and STAR Methods.

Top 5 most significant pathways per factor based on gene ontology (biological processes) and the cancer hallmark gene set collection from MsigDB. Terms from the hallmark gene set collection are highlighted with bold text with the epithelial to mesenchymal transition (EMT) term highlighted with green boxes. The GeneRatio is defined as the number of genes shared between the top

contributing genes per factor and the term gene set divided by the total number of top contributing genes.

A

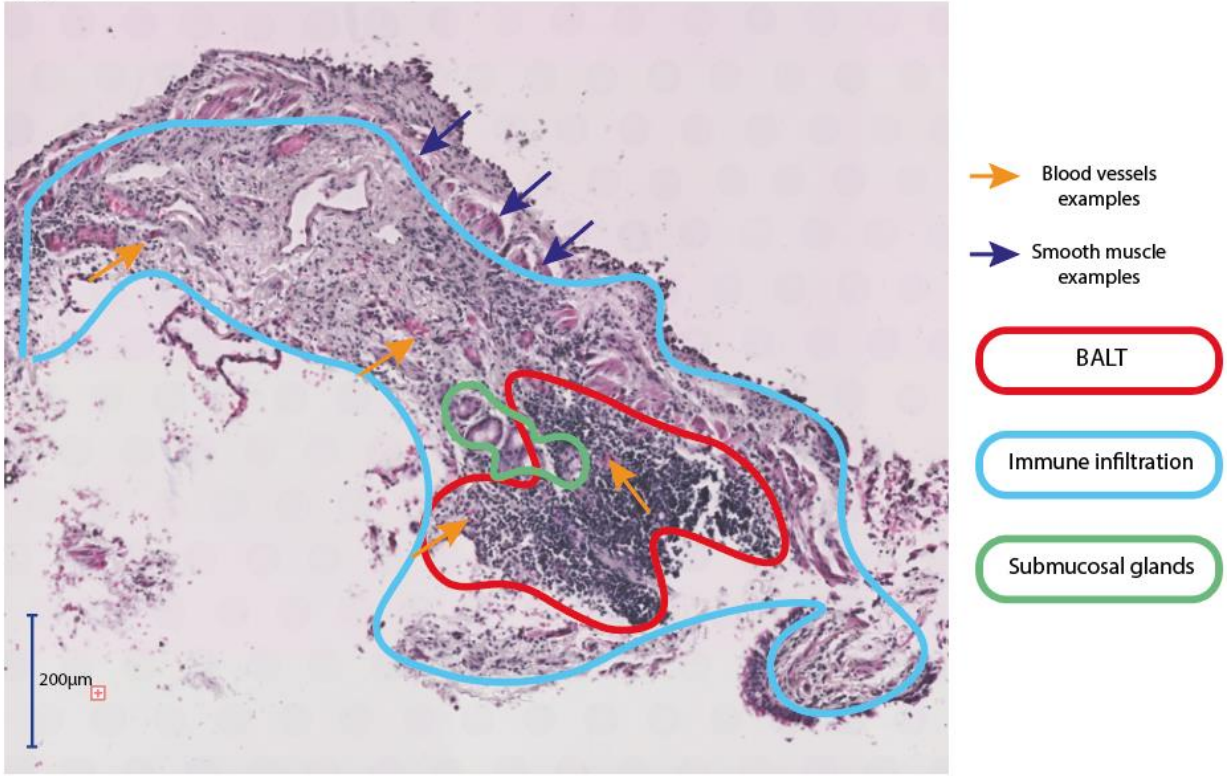
200μm
Blood vessels examples
Smooth muscle examples
BALT
Immune infiltration
Submucosal glands

B

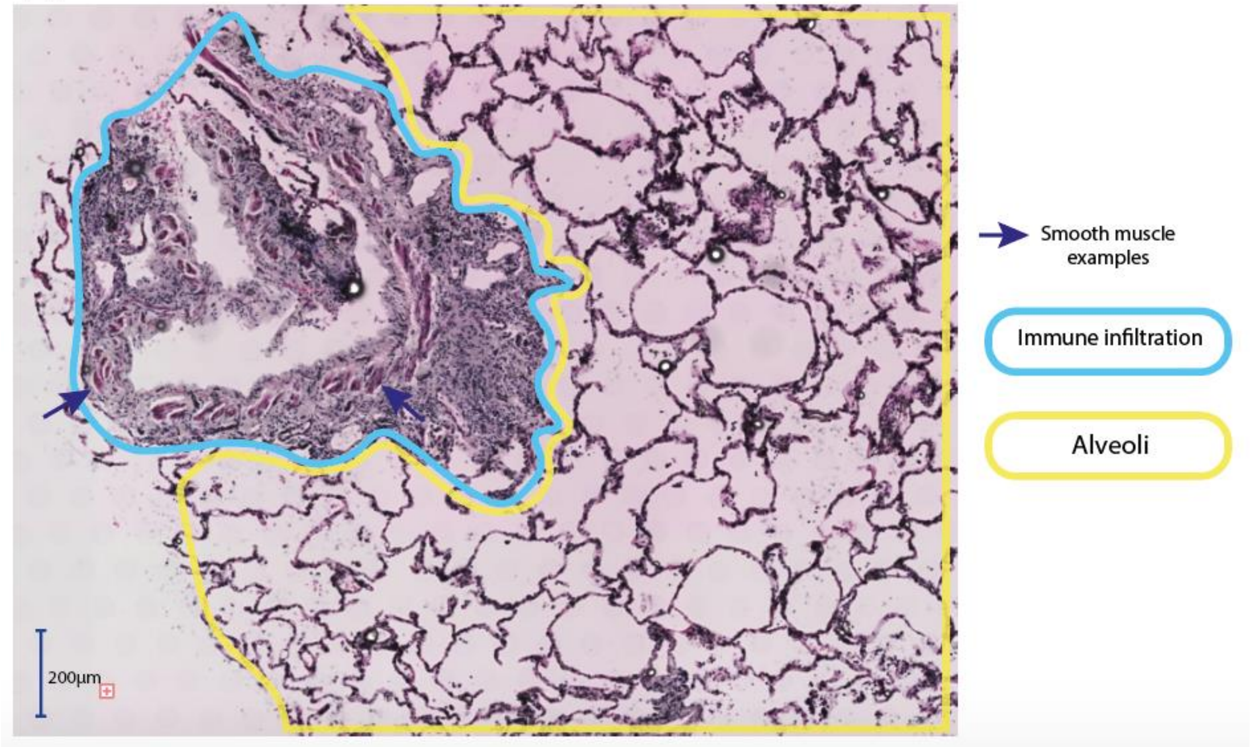
200μm
Smooth muscle examples
Immune infiltration
Alveoli

Figure S15. Annotation of H&E stained Lung from Covid-19 patient biopsy, related to Figure 4 and STAR Methods.

(A) A fragment from bronchus with morphological damage (most likely a technical artifact not associated with the disease). Bronchus Associated Lymphoid Tissue (BALT) is observed, a few submucosal glands, vessels and a densely packed lymphoid follicle are found in the BALT area.

(**B**) Alveolar region and partial bronchioles with damaged morphology presenting extensive lymphocytic infiltration around the area.

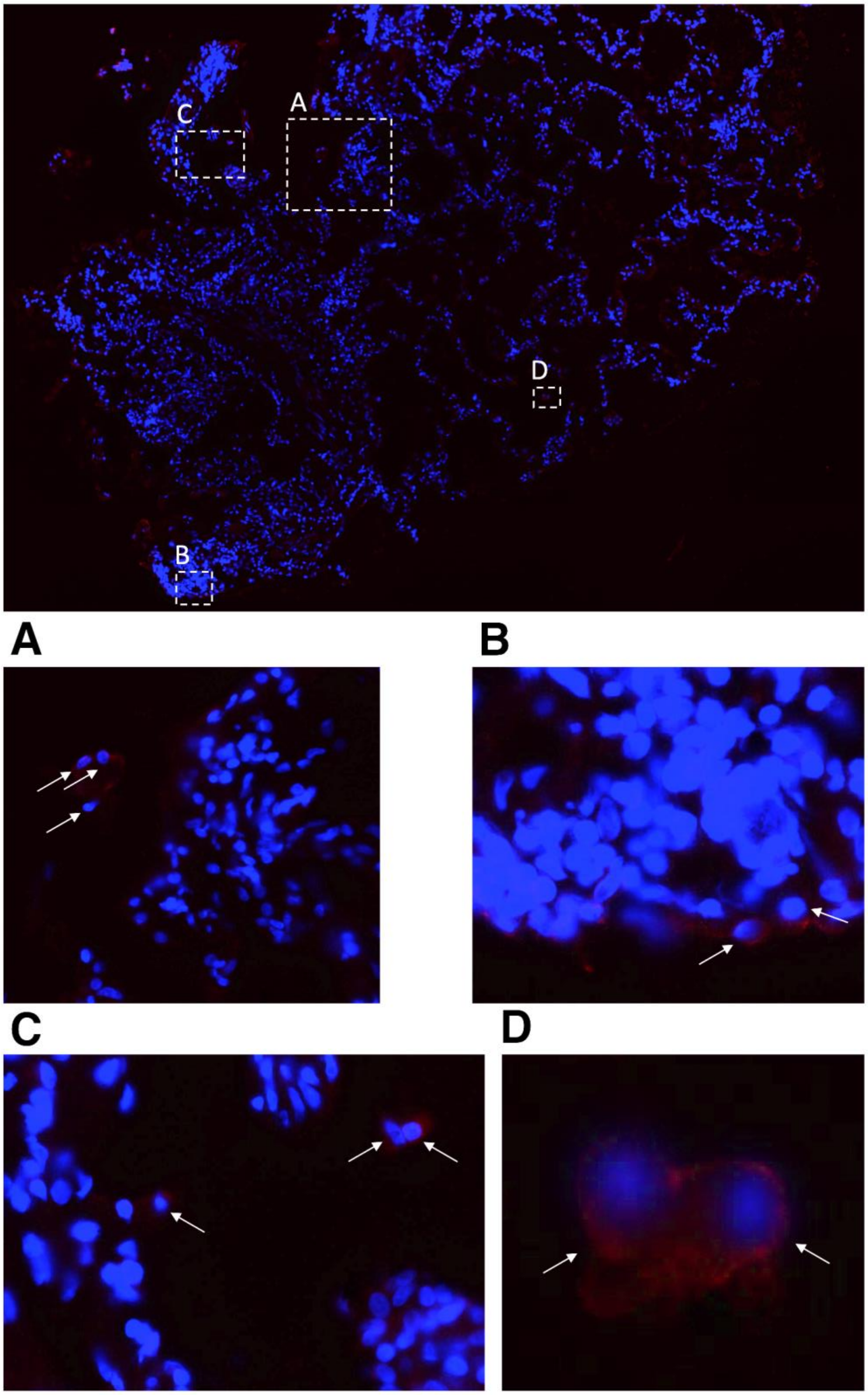
C
A
D
B
A
B
C
D

Figure S16. Immunofluorescent staining of SARS-CoV-1 spike protein, related to Figure 4 and STAR Methods.

Background has been removed by altering color balance (see methods).

Zoomed in areas (A)-(D), Examples of positive cells (red) marked by white arrows.

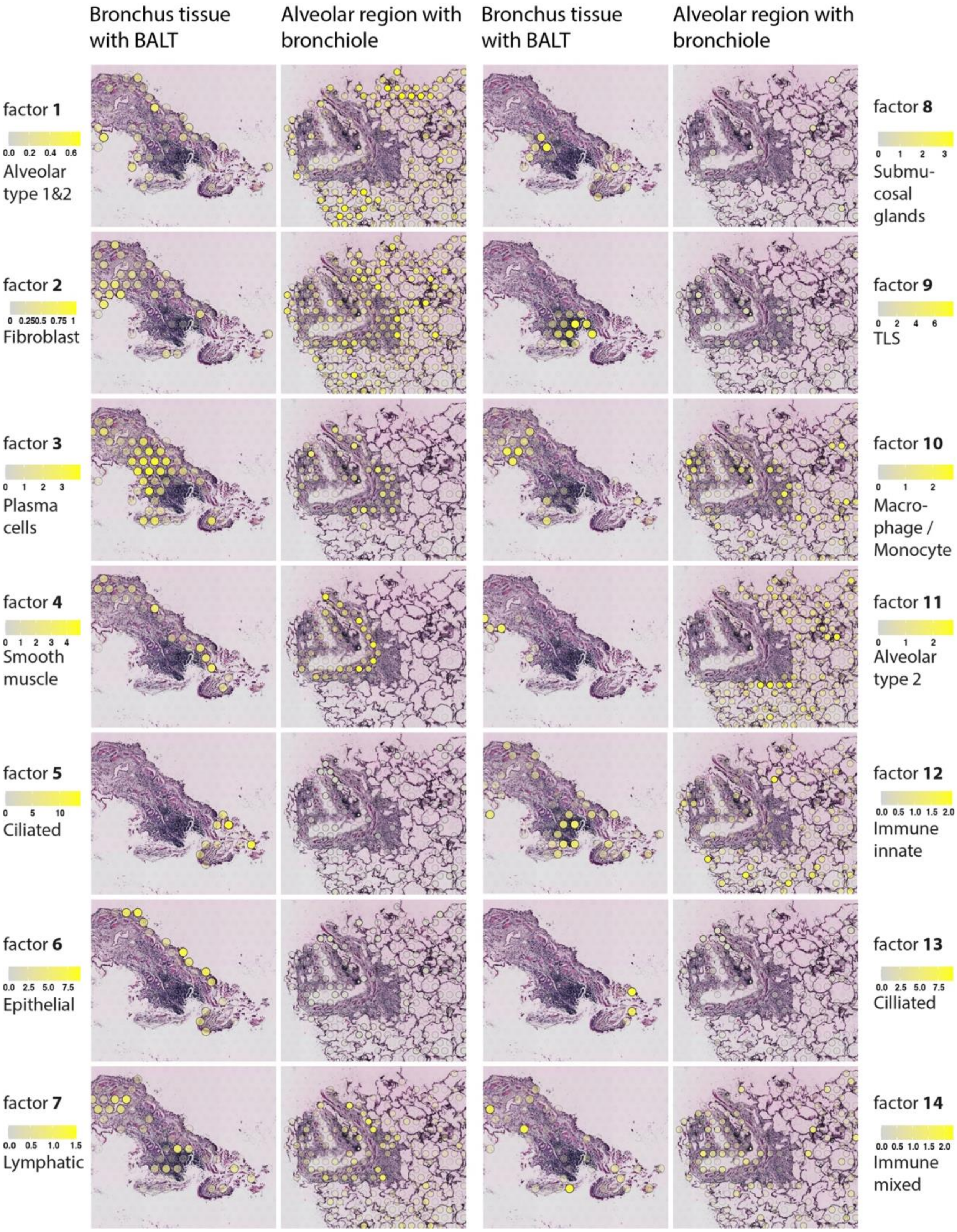

Figure S17. Spatial factor activity maps overlaid on H&E images of lung from COVID-19 PCR-positive patient, related to Figure 4 and STAR Methods.

Two main tissue regions are shown; bronchus tissue with a BALT structure and an alveolar region with a bronchiole. Each factor is visualized as a spatial activity map (yellow dots) and has been scaled to make the spots gradually opaque, i.e. spots with a value of 0 are 100% transparent while spots with a maximum factor activity value are opaque.

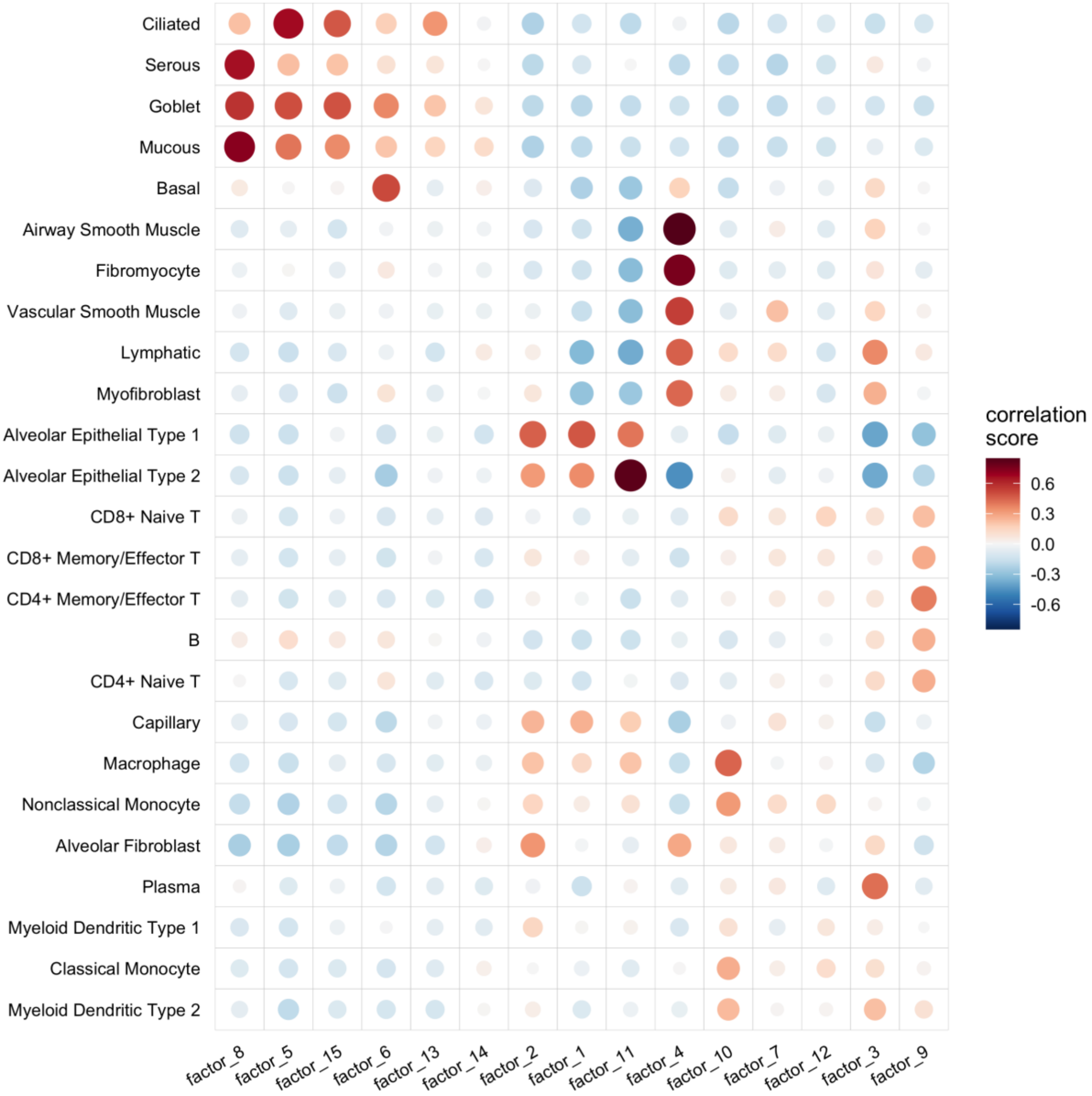

Figure S18. Correlation between cell type enrichment scores and factors in lung tissue infected with Covid-19, related to Figure 4 and STAR Methods.

The x axis represents 25 cell types from a molecular cell atlas of the human lung. The y axis represents factors computed using Non-negative Matrix Factorization of the spatially resolved gene expression data. The color and size of each dot represents the strength of the Pearson correlation coefficient between log-transformed cell type enrichment scores (AUC) and log-transformed factor activity scores.

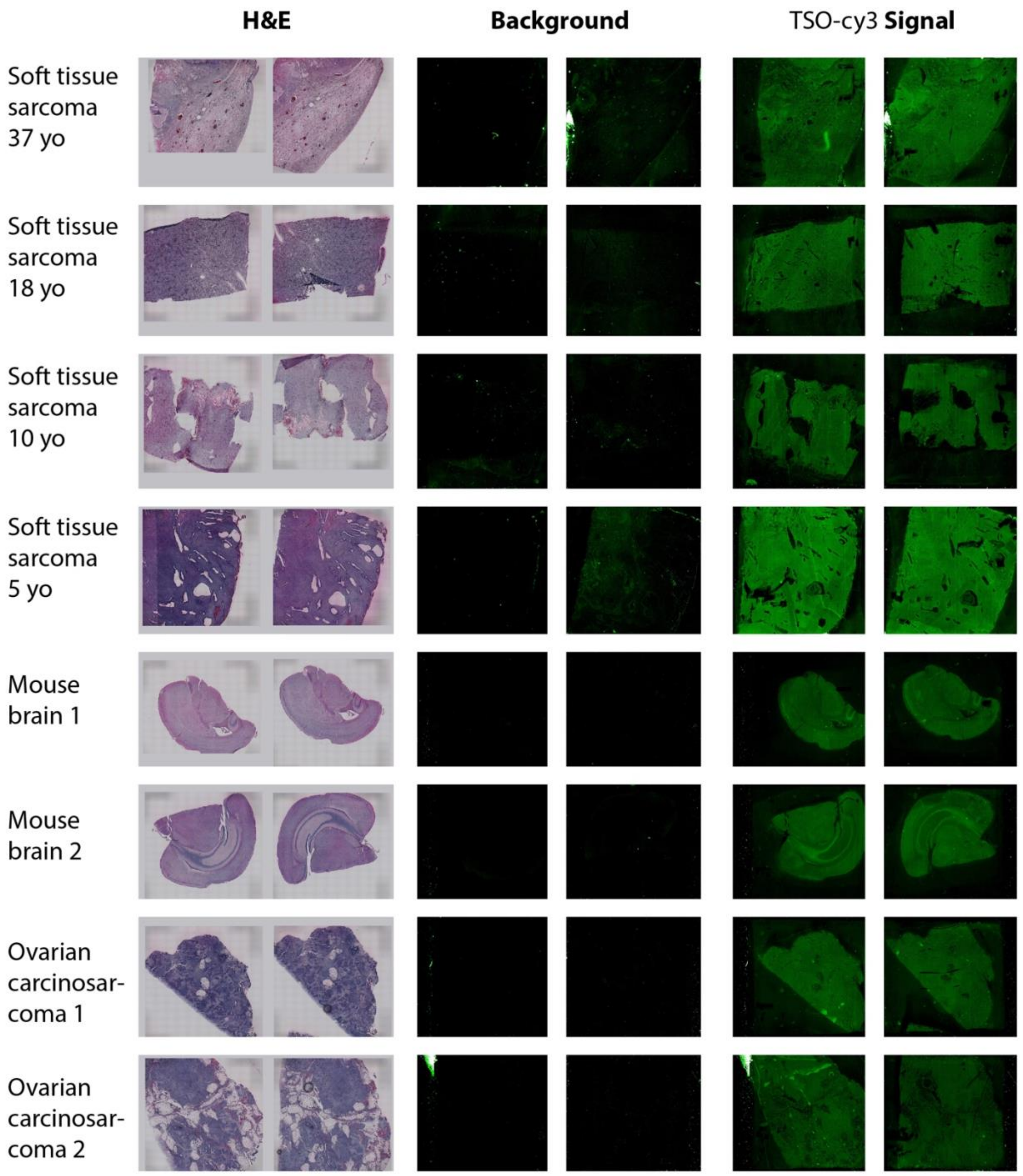

Supplemental Figure 19 | cy3-TSO QC assay applied on different FFPE tissue types, related to STAR Methods.

Each row shows duplicate sections collected from a different tissue block. H&E images of the duplicate tissue section are shown in the left column. During reverse transcription of mRNA, a

cDNA strand is synthesized and a complementary to TSO sequence added in the 3´end. Then, the tissue is removed and the mRNA templates are de-hybridized. Slides are imaged to ensure there is no tissue left over the slide. Fluorescence scans of the negative control/autofluorescence after enzymatic tissue removal, i.e. without cy3-TSO added, are shown in the middle column (background). Finally, a fluorescent cy3-TSOprobe is hybridized and the cDNA signal image is taken. Fluorescence scans of the cy3-TSO cDNA footprints are shown in the right column (TSO-Cy3-signal).

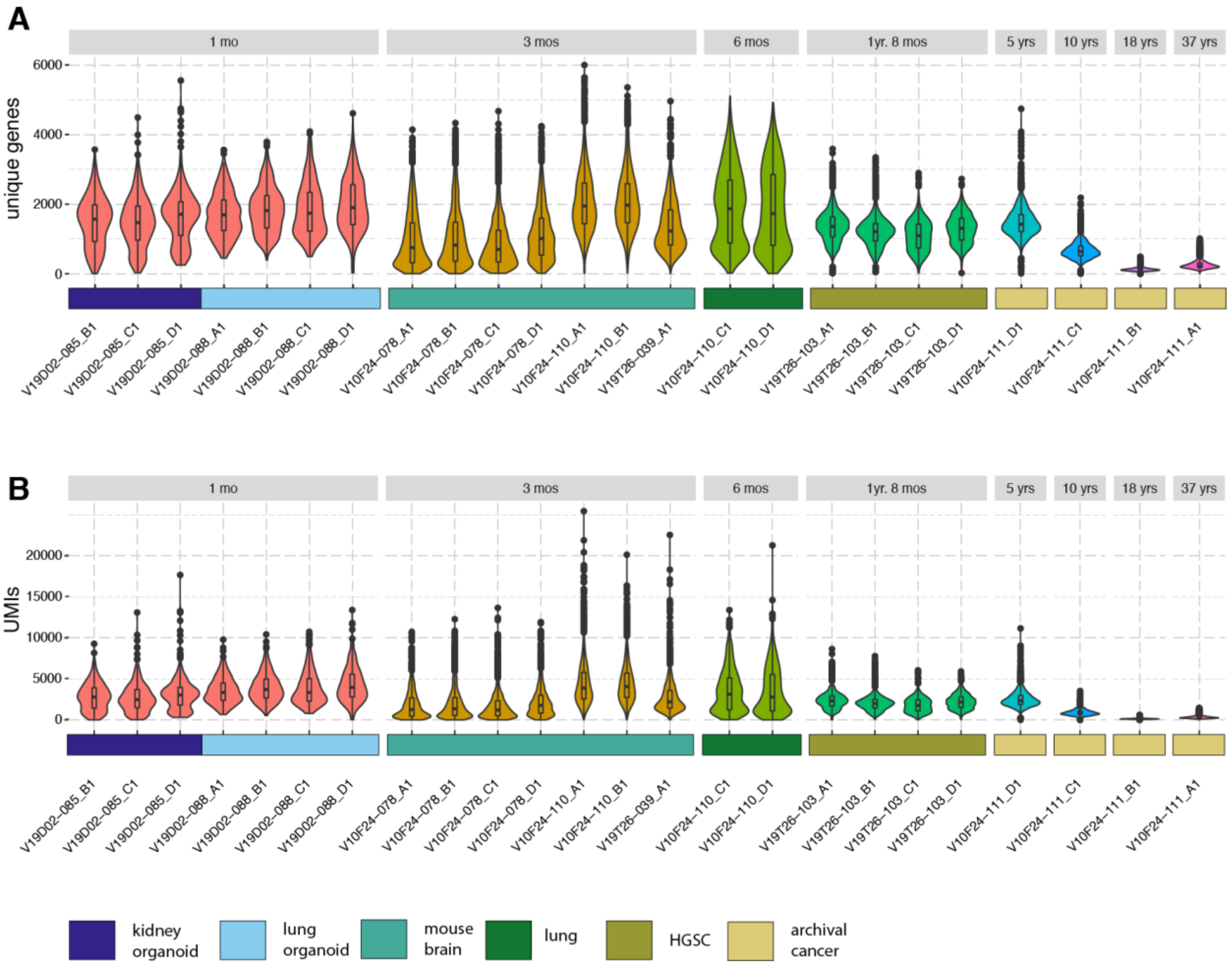

Figure S20 | Quality metrics by storage time, related to STAR Methods.

Quality metrics for 6 tissue types stored between 1 month and 37 years

(A) Unique genes per spot distributions.

(B) UMIs per spot distributions.
